# Ligand‐Promoted [Pd]‐Catalyzed α‐Alkylation of Ketones through a Borrowing‐Hydrogen Approach

**DOI:** 10.1002/open.202200245

**Published:** 2023-01-02

**Authors:** Seetharaman Manojveer, Nitish K. Garg, Zarif Gul, Ayesha Kanwal, Yogesh Goriya, Magnus T. Johnson

**Affiliations:** ^1^ Centre for Analysis and Synthesis Department of Chemistry Lund University P. O. Box 124 221 00 Lund Sweden; ^2^ Perstorp AB Perstorp Industrial Park 284 80 Perstorp Sweden

**Keywords:** α-alkylation, borrowing-hydrogen, catalysis, 2-hydroxypyridine, MLC

## Abstract

A new class of palladium complexes bearing bidentate 2‐hydroxypyridine based ligands have been prepared and fully characterized. The applications of these new complexes towards ketone alkylation reactions with alcohols through a metal‐ligand cooperative borrowing‐hydrogen (BH) process were demonstrated.

## Graphical abstract

**A new class of palladium complexes** bearing 2‐hydroxypyridine‐based ligands has been prepared and fully characterized. The palladium complex **1‐[Pd]** (top) has been shown to be an active catalyst for the direct α‐alkylation of ketones with alcohols through the borrowing‐hydrogen approach. The involvement of a metal‐ligand cooperativity pathway was supported by comparison between complexes **1‐[Pd]** and **4‐[Pd]** (bottom), where the absence of the hydroxyl moiety in **4‐[Pd]** led to a decreased activity.

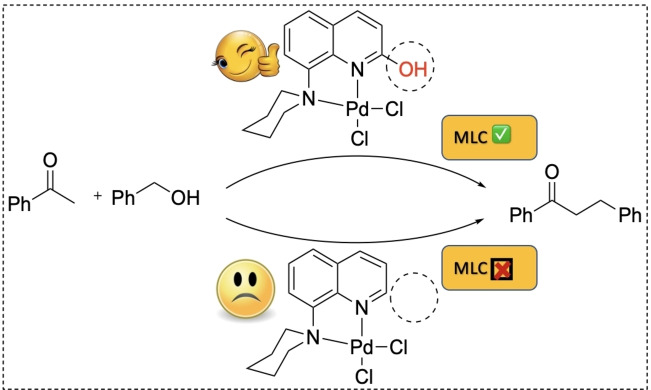

## Introduction

**Scheme 1:**
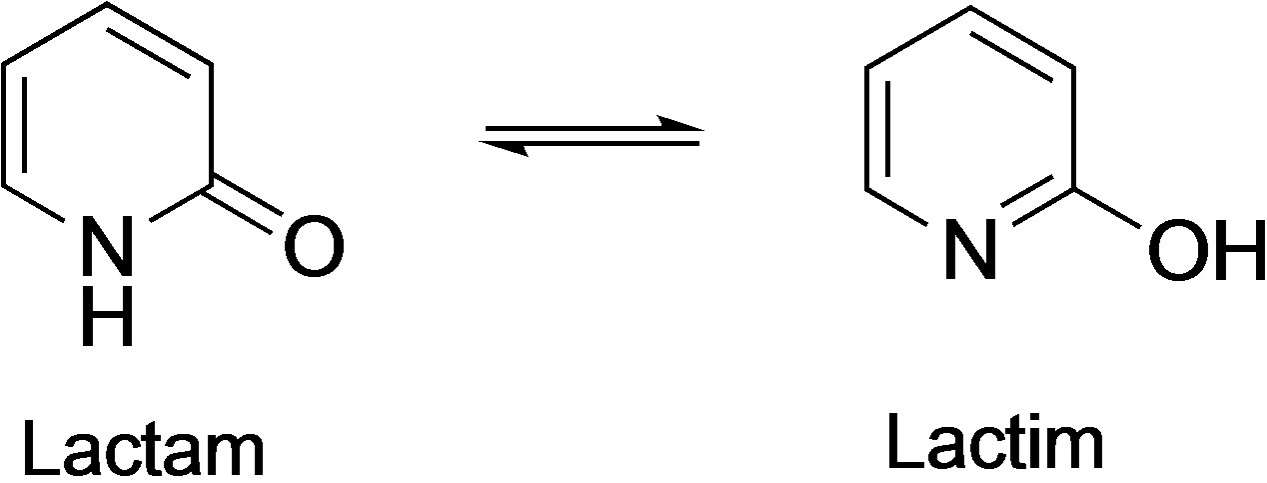
Lactam and lactim tautomerization.

Metal‐ligand cooperation (MLC), where both the metal and ligand are directly involved in the bond activation process, has become increasingly important in transition metal catalysis for hydrogen transfer reactions. Different metal/ligand systems have been reported for the activation of small molecules like hydrogen, carbon dioxide and others.^[1]^ Among these, 2‐hydroxypyridine‐based ligands have received significant attention in the last decade as they can undergo tautomerization between the lactam and lactim forms to promote the catalysis (Scheme 1). When coordinated by a metal center, the resulting complex is active in hydrogenation and dehydrogenation processes through metal‐ligand cooperation.^[2]^

In comparison to other transition metals, iridium and ruthenium complexes with the 2‐hydroxypyridine backbone have been studied extensively.[^1^, ^2^] For example, Yamaguchi and Fujita have reported iridium complexes bearing 2‐hydroxypyridine‐based ligands that promoted the dehydrogenation of alcohols to ketones under neutral conditions with high turnover numbers. Szymczak and co‐workers have developed a ruthenium‐based catalyst for selective transfer hydrogenation the in situ formed pyridone from 2‐hydroxypyridine could promote the hydrogenation of ketone via coordination with alkali metals.^[2]^ Recently, the groups of Achard^[3]^ and Kundu^[4]^ have prepared different ruthenium complexes with the 2‐hydroxypridine motif and demonstrated that those are effective catalysts for the *C*‐alkylation of ketones and *N*‐alkylation of amines with alcohols through the borrowing‐hydrogen (BH) process.

**Figure 1:**
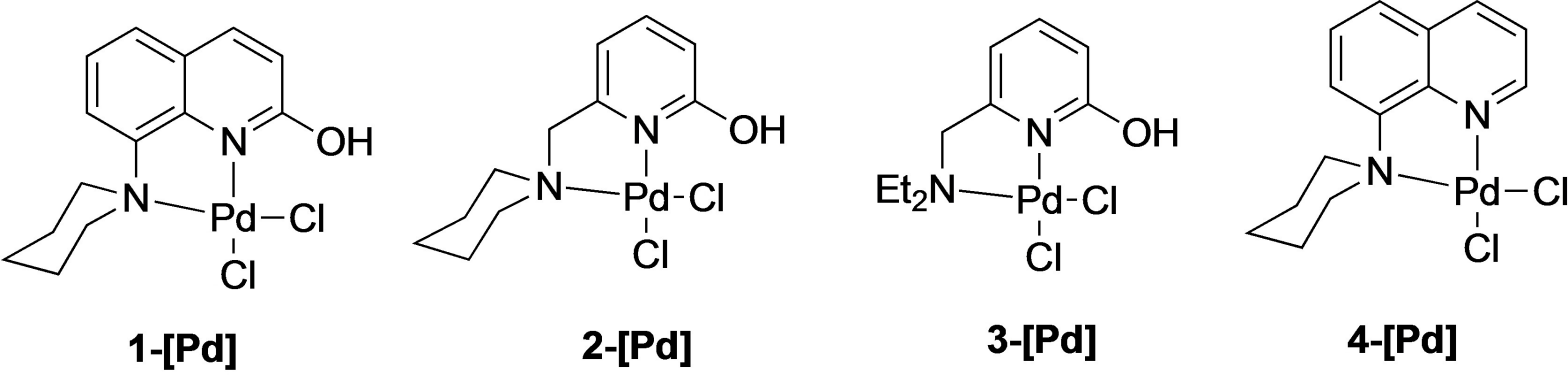
Novel palladium complexes synthesized for this study.

Notably, the utilization of palladium complex with 2‐hydroxypyridine‐based ligand backbones has never been reported for the MLC strategy. Since the BH process involves dehydrogenation and hydrogenation steps, we were interested to explore whether palladium complexes bearing the 2‐hydroxypyrine moiety would be active in hydrogen transfer reactions. More specifically, direct α‐alkylation of ketones using alcohols as the coupling partner was of interest. Traditionally, either a ketone or a hydroxyl group of an alkyl partner is pre‐activated to achieve the alkylation of ketones. However, this is an environmentally unfriendly method generating significant amounts of waste.[^5^, ^6^] To overcome these drawbacks, alkylation of ketones and amines with alcohols have been developed using various transition metal catalysts.[^7^, ^8^, ^9^, ^10^, ^11^, ^12^, ^13^] For example, Beller and coworkers reported cyclometalated ruthenium pincer complexes for the α‐alkylation of ketones^[7a]^ and Milstein and coworkers have reported a manganese pincer catalyst for the α‐alkylation of ketones using primary alcohols.^[10b]^ Recently, Gülcemal and coworkers reported the use of [PdCl_2_(NHC)(py)] complexes as catalyst precursors for the α‐alkylation of ketones with primary alcohols.^[9a]^ In spite of their broader application for the oxidation of alcohols using an external oxidant, palladium complexes have rarely been explored for the alkylation of ketones with alcohols.[^14^, ^15^] Herein, we report the preparation of novel palladium complexes bearing the 2‐hydroxypyridine scaffold operating via MLC for the alkylation of ketones (Figure 1). The Pd complexes were fully characterized using SCXRD, HRMS and NMR spectroscopy.

## Results and Discussion

**Scheme 2:**
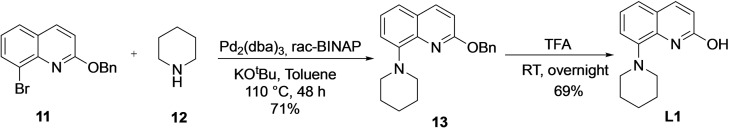
Preparation of ligand **L1**.

The synthetic steps for the preparation of ligand **L1** are depicted in Scheme 2. Following literature procedures, compound **11** was obtained in 82 % yield starting from 2‐bromoaniline and *trans*‐cinnamoyl chloride.[^16^, ^17^] Furthermore, the Pd‐catalyzed C−N coupling was carried out between compound **11** and piperidine (**12**) to afford product **13** in 71 % yield. Finally, the compound **13** underwent debenzylation in TFA at room temperature to furnish ligand **L1** in 69 % yield.^[18]^

**Scheme 3:**
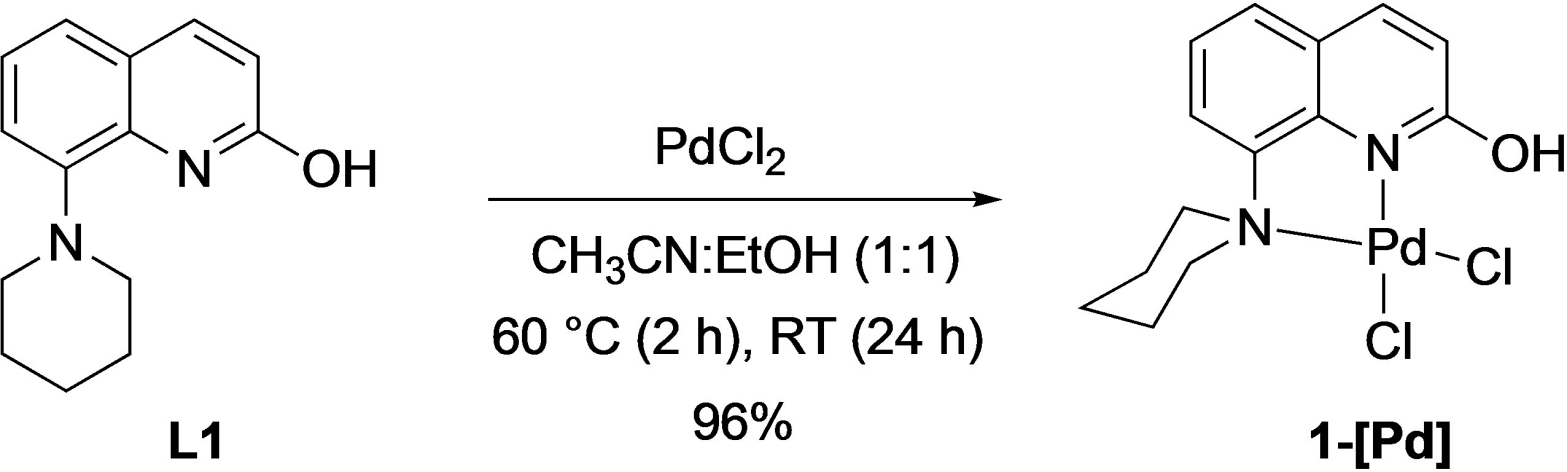
Synthesis of complex **1‐[Pd]**.

**Figure 2:**
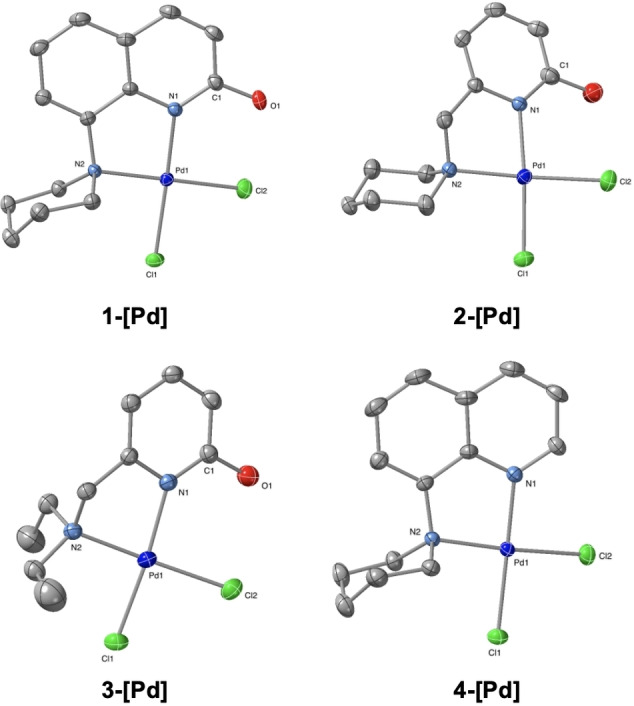
Crystal structure of the Pd complexes. Displacement ellipsoids correspond to 30 % probability. Hydrogen atoms are omitted for clarity.

The **1‐[Pd]** complex was obtained in 96 % yield by treating **L1** with PdCl_2_ in a CH_3_CN:EtOH (1 : 1) mixture for 2 h at 60 °C and 24 h at room temperature (Scheme 3). A similar synthetic approach was followed to synthesize **2‐[Pd]** to **4‐[Pd]** as described in the Supporting Information. Crystal structures of all four complexes have been determined from suitable single crystals and are shown in Figure 2.

Having the desired complexes in hand, the next goal was to test the catalytic activity of our novel palladium complexes for the α‐alkylation of ketones with alcohols. Preliminary studies conducted with acetophenone and benzyl alcohol as substrates in toluene showed promising results as the formation of the alkylated product **3 a** was observed by GC‐MS. The reaction was further optimized as shown in Table 1. At the outset, the reactions were studied with a sub‐stoichiometric amount of base and product **3 a** was obtained in low yield (Table 1, entries 1–5). Based on these outcomes, KO^t^Bu was chosen as the base for further optimization studies. The yield of the alkylated product increased to 86 % when the reaction was performed in the presence of stoichiometric amounts of base (entry 6). Blank reactions conducted in the absence of catalyst or with PdCl_2_ as catalyst resulted in significantly lower yield or selectivity (entries 8 and 9). Using **2‐[Pd]** as catalyst with the optimized conditions resulted in 84 % yield, whereas using **3‐[Pd]** and **4‐[Pd]** resulted in a mixture of products **3 a** and **4** and, hence, these complexes were deemed less selective (entries 10–12). From these results, the conclusion was drawn that the OH group in the **1‐[Pd]** complex is capable of promoting both dehydrogenation and hydrogenation for the selective formation of product **3 a** in good yields. The reaction was further conducted in different solvents, and dioxane and *t*‐amyl alcohol were found inferior to toluene (entries 14 and 15). Finally, product **3 a** was isolated in 94 % yield when performing the reaction in the presence of molecular sieves using 2 mol % of complex **1‐[Pd]** and 1.0 equiv. KO^*t*^Bu after 48 h reflux at 120 °C in toluene (entry 16).

**Table 1:** Optimization studies for the α‐alkylation of ketones with primary alcohols.^[a]^

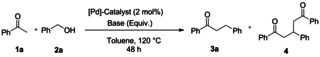				
S. No.	Catalyst	Base (equiv.)	Product [%]^[b]^	
			**3 a**	**4**
1	1‐[Pd]	KO^t^Bu (0.25)	22	—
2	1‐[Pd]	NaO^t^Bu (0.25)	20	
3	1‐[Pd]	KOH (0.25)	16	—
4	1‐[Pd]	Cs_2_CO_3_ (0.25)	24	28^[c]^
5	1‐[Pd]	KO^t^Bu (0.50)	39	—
6	1‐[Pd]	KO^t^Bu (1.0)	88	—
7	1‐[Pd]	KOH(1.0)	47	48
8	—	KO^t^Bu (1.0)	25	25
9	PdCl_2_	KO^t^Bu (1.0)	62	34
10	2‐[Pd]	KO^t^Bu (1.0)	84	—
11	3‐[Pd]	KO^t^Bu (1.0)	13	20
12	4‐[Pd]	KO^t^Bu (1.0)	59	12
13^[d]^	1‐[Pd]	KO^t^Bu (1.0)	43	47
14^[e]^	1‐[Pd]	KO^t^Bu (1.0)	24	—
15^[f]^	1‐[Pd]	KO^t^Bu (1.0))	45	—
**16^[g]^**	**1‐[Pd]**	**KO^t^Bu (1.0)**	**99 (94)^[h]^**	—

[a] All the reactions were carried out using 2 mol % catalyst in toluene at 120 °C for 48 h. [b] NMR yield using ferrocene as an internal standard. [c] Yield of benzyl benzoate. [d] In situ generation of catalyst. [e] In dioxane. [f] In *t*‐amyl alcohol. [g] In the presence of molecular sieves. [h] Isolated yield.

**Figure 3:**
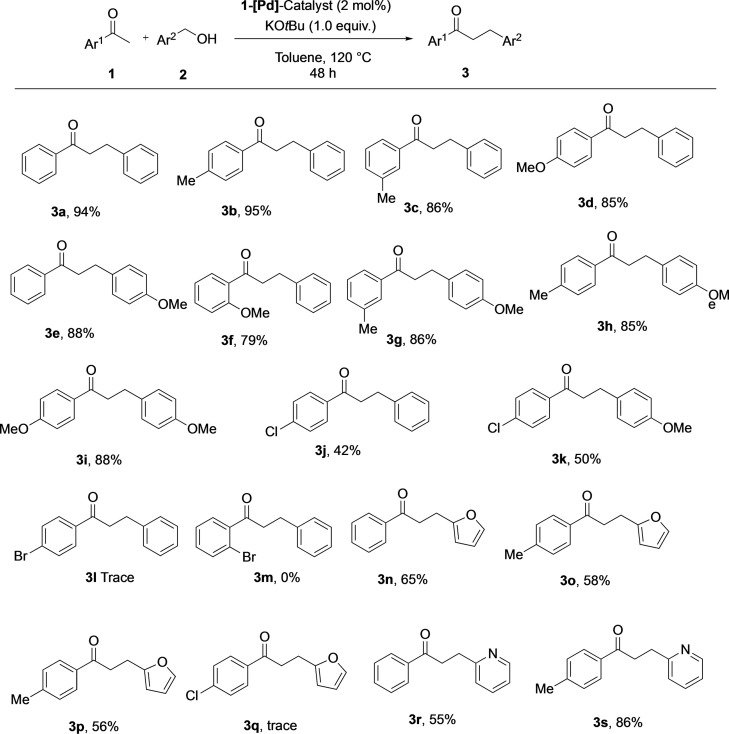
Substrate scope of the Pd‐catalyzed α‐alkylation of alcohols with ketones.

Next, the optimized reaction conditions were applied to different substrates to explore the scope of the catalytic system in the alkylation of acetophenones using benzyl alcohols as the alkylating partner. As shown in Figure 3, methyl‐ and methoxy‐substituted acetophenone and benzyl alcohol derivatives tolerated the present reaction conditions and gave the corresponding alkylated products in high yields (**3 a**–**3 i**). The reactions of 4‐chloro‐acetophenone with benzyl alcohol and 4‐methoxylbenzyl alcohol furnished the respective alkylated products (**3 j** and **3 k**) in 42 % and 50 % yield, respectively. Unfortunately, only trace amounts of the 4‐bromo‐substituted product **3 l** were observed. 2‐Bromoacetophenone did not undergo any transformation with benzyl alcohol under the optimized reaction conditions. It is worth noting that furfuryl alcohol could effectively be utilized for alkylation of acetophenone derivatives to get the products (**3 n**–**3 p**) in moderate yields. Similarly, 2‐pyridinemethanol was used as an alkylating partner for acetophenones to form products **3 r** and **3 s** in 55 % and 86 % yield, respectively.

**Scheme 4:**
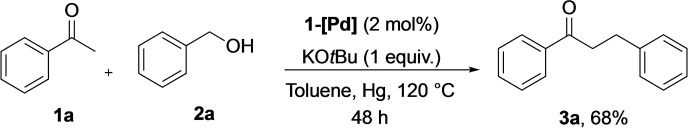
Optimized standard reaction that was subjected to the Hg‐test.

To check the heterogeneous nature of the present catalytic transformation, a Hg test was performed (Scheme 4). The reaction of acetophenone and benzyl alcohol furnished the product **3 a** in 68 % yield under the optimized reaction conditions in the presence of Hg. While this represents a reduction in yield, it is not a complete shutdown of reactivity and we therefore conclude that, while a heterogeneous nature of the active catalyst species cannot be fully excluded, it is likely that the catalyst is primarily homogeneous in nature.^[19]^

**Scheme 5:**
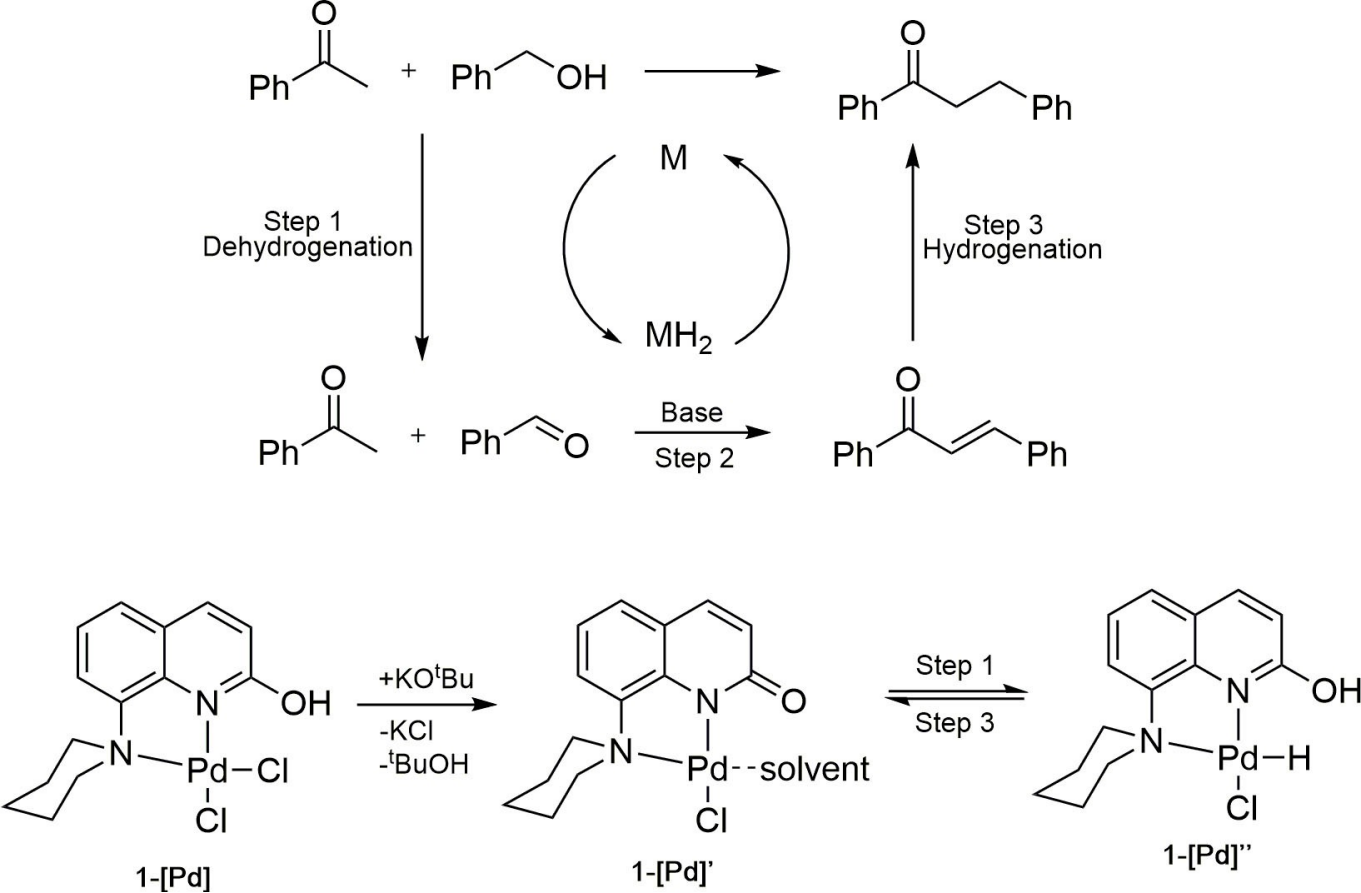
Proposed reaction mechanism for the palladium‐catalyzed α‐alkylation of ketones with alcohols.

Based on previous literature reports on similar reactions, a plausible mechanism for the palladium catalyzed α‐alkylation of ketones with alcohols has been proposed as shown in Scheme 5.[^9a^, ^20^] Step 1 is the dehydrogenation of the alcohol to form the respective aldehyde followed by the aldol condensation and the last step is the hydrogenation to form the product. The catalyst **1‐[Pd**] is possibly converted into **1‐[Pd]’** in the presence of base and it probably coordinates solvent as shown in Scheme 5. **1‐[Pd]’** now has a basic site and can further deprotonate the alcohol to form the palladium alkoxide intermediate which then undergoes a β‐hydride elimination to form **1‐[Pd]’’**.

## Conclusion

In summary, we have synthesized a new class of palladium complexes bearing the 2‐hydroxypyridine motif which allows for metal‐ligand cooperativity (MLC). The palladium complex **1‐[Pd]** has been shown to be an active catalyst for the direct α‐alkylation of acetophenones using benzyl alcohols as coupling partners through the borrowing‐hydrogen approach. The involvement of the MLC pathway was supported by comparison between complexes **1‐[Pd]** and **4‐[Pd]**, where the absence of the hydroxyl moiety in **4‐[Pd]** led to decreased activity.

## Supporting Information Summary

The Supporting Information contains detailed experimental procedures, details on the X‐ray crystallography and copies of all relevant ^1^H and ^13^C NMR spectra.

Deposition Numbers 2104033 (for **2‐[Pd]**), 2104035 (for **3‐[Pd]**), 2108795 (for **4‐[Pd]**), and 2209156 (for **1‐[Pd]**) contain the supplementary crystallographic data for this paper. These data are provided free of charge by the joint Cambridge Crystallographic Data Centre and Fachinformationszentrum Karlsruhe Access Structures service.

## Conflict of interest

The authors declare no conflict of interest.

1

## Supporting information

As a service to our authors and readers, this journal provides supporting information supplied by the authors. Such materials are peer reviewed and may be re‐organized for online delivery, but are not copy‐edited or typeset. Technical support issues arising from supporting information (other than missing files) should be addressed to the authors.

Supporting Information

## Data Availability

The data that support the findings of this study are available in the supplementary material of this article.
