# Supplementary material for: Ligand‐Promoted [Pd]‐Catalyzed α‐Alkylation of Ketones through a Borrowing‐Hydrogen Approach
Source: ChemistryOpen. 2023 Jan 2;12(1):e202200245. doi: 10.1002/open.202200245 (PMC9807026; doi:10.1002/open.202200245)
Supplement: Supplementary file 1 — Supporting Information [file OPEN-12-e202200245-s001.pdf]

ChemistryOpen

Supporting Information

Ligand-Promoted [Pd]-Catalyzed α-Alkylation of Ketones through a Borrowing-Hydrogen Approach

Seetharaman Manojveer, Nitish K. Garg, Zarif Gul, Ayesha Kanwal, Yogesh Goriya, and Magnus T. Johnson*

Table of contents

1. General considerations

2. General procedure for [Pd]-Catalysis

3. Analytical data of compound **3**

4. Preparation of Palladium-complexes

5. X-ray crystal structures

6. References

7. Copy of ^{1}H- and ^{13}C-NMR spectra

1. General considerations

Unless otherwise noted, all solvents were freshly distilled under vacuum from sodium/benzophenone ketyl radical, except acetonitrile which was dried over calcium hydride. All experiments were carried out under an atmosphere of argon or nitrogen using standard Schlenk or high vacuum line techniques unless otherwise noted. Unless stated otherwise, commercially available reagents were purchased from Sigma Aldrich or Acros Organics and used as received. NMR-spectra were recorded on Bruker Ascend 500 MHz, Varian Unity INOVA 500 MHz or Bruker Advance 400 MHz spectrometers. Multiplicities are abbreviated as follows: (s) singlet, (d) doublet, (t) triplet, (q) quartet, (m) multiplet, (b) broad. IR spectra were recorded on a Bruker Alpha spectrometer, with diamond ATR–FT IR detection. Gas chromatographic analyses (GC) were made using a Hewlett- Packard 5890 II instrument with a flame ionization detector (FID) and a capillary column (CP-Sil 19CB 14% cyanopropyl-phenyl/86% dimethylpolysiloxane, 0.2 μm, 0.2 mm, 25 m) with decane as an internal standard. The retention times of different compounds in the gas chromatogram were identified using commercially available and synthesized pure compounds. X-ray data was collected with an Oxford Diffraction Excalibur 3 system, using ω scans and Mo Kα (λ = 0.71073 Å) radiation. The data were extracted and integrated using Crysalis RED. The structures were solved by direct methods and refined by full-matrix least-squares calculations on F^2 using SHELXT, SHELXL and OLEX2.

2. General procedure for [Pd]-Catalysis

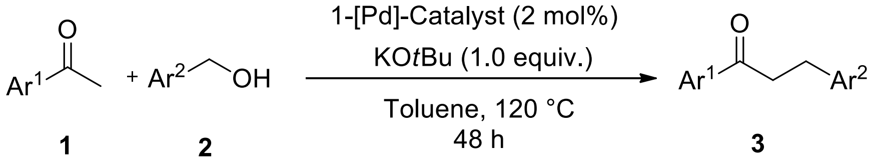

To a solution of catalyst 1-[Pd] (2 mol%), KO*t*Bu (1.0 equiv.) and molecular sieves in toluene, acetophenone **1** (1.0 equiv.) and benzyl alcohol **2** (2.0 equiv.) were added at room temperature. Then, reaction mixture was refluxed at 120 °C for 48 h under nitrogen atmosphere. After 48 h, water was added to reaction mixture and the compound was extracted using EtOAc. Then, the crude was purified by column chromatography (silica gel, hexanes/EtOAc) to furnish the pure compound **3**.

3. Analytical data of compound 3

1,3-Diphenylpropan-1-one 3a:[1]

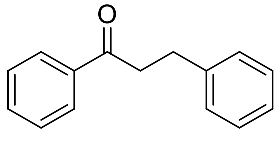

It was obtained as a colourless solid in 94% yield (79 mg). ^{1}H NMR (400 MHz, $CDCl_3$): δ 7.97 (d, J = 7.2 Hz, 2H), 7.58-7.54 (m, 1H), 7.46 (t, J = 7.2 Hz, 2H), 7.33-7.25 (m, 4H), 7.23-7.19 (m ,1H), 3.31 (t, J = 6.8 Hz, 2H), 3.08 (t, J = 7.2 Hz, 2H); ^{13}C NMR (100 MHz, $CDCl_3$): δ 199.2, 141.3, 136.8, 133.0, 128.6, 128.5, 128.4, 128.0, 126.1. 40.4, 30.1.

3-Phenyl-1-(p-tolyl)propan-1-one 3b:[1]

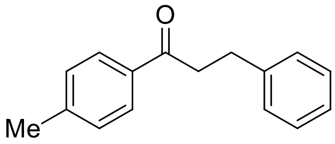

It was obtained as a colourless solid in 95% yield (79 mg). ^{1}H NMR (400 MHz, $CDCl_3$): δ 7.86 (d, J = 8.4 Hz, 2H), 7.33-7.20 (m, 7H), 3.28 (t, J = 6.8 Hz, 2H), 3.06 (t, J = 7.2 Hz, 2H), 2.41 (s, 3H); ^{13}C NMR (100 MHz, $CDCl_3$): δ 198.9, 143.8, 141.4, 134.4, 129.5, 128.5, 128.4, 128.1, 126.1, 40.3, 30.2, 21.6.

3-Phenyl-1-(m-tolyl)propan-1-one 3c:[2]

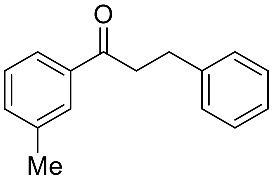

It was obtained as a colourless solid in 86% yield (72 mg). ^{1}H NMR (400 MHz, $CDCl_3$): δ 7.78-7.75 (m, 2H), 7.39-7.20 (m, 7H), 3.30 (t, J = 8.4 Hz, 2H), 3.07 (t, J = 8.0 Hz, 2H), 2.41 (s, 3H); ^{13}C NMR (100 MHz, $CDCl_3$): δ 199.4, 141.3, 138.3, 136.9, 133.8, 128.55, 128.48, 128.44, 128.40, 126.1, 125.2, 40.5, 30.1, 21.3.

1-(4-Methoxyphenyl)-3-phenylpropan-1-one 3d:[1]

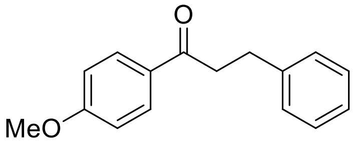

It was obtained as a colourless solid in 85% yield (68 mg). ^{1}H NMR (400 MHz, $CDCl_3$): δ 7.95 (d, J = 8.8 Hz, 2H), 7.32-7.21 (m, 5H), 6.93 (d, J = 9.2 Hz, 2H), 3.87 (s, 3H), 3.26 (t, J = 7.2 Hz, 2H), 3.06 (t, J = 7.6 Hz, 2H); ^{13}C NMR (100 MHz, $CDCl_3$): δ 197.8, 163.4, 141.4, 130.3, 129.9, 128.5, 128.4, 126.0, 113.7, 55.4, 40.1, 30.3.

3-(4-Methoxyphenyl)-1-phenylpropan-1-one 3e:[1]

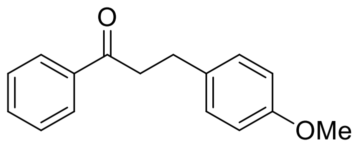

It was obtained as a colourless solid in 85% yield (87 mg). ^{1}H NMR (400 MHz, $CDCl_3$): δ 7.96 (d, J = 6.8 Hz, 2H), 7.58-7.53 (m, 1H), 7.45 (t, J = 8.0 Hz, 2H), 7.17 (d, J = 8.8 Hz, 2H), 6.84 (d, J = 8.8 Hz, 2H), 3.79 (s, 3H), 3.27 (t, J = 6.8 Hz, 2H), 3.02 (t, J = 7.2 Hz, 2H); ^{13}C NMR (100 MHz, $CDCl_3$): δ 199.4, 158.0, 136.9, 133.3, 133.0, 129.3, 128.6, 128.0, 113.9, 55.3, 40.7, 29.3.

1-(2-Methoxyphenyl)-3-phenylpropan-1-one 3f:[3]

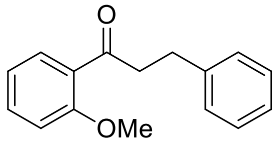

It was obtained as a colourless solid in 85% yield (63 mg). ^{1}H NMR (400 MHz, $CDCl_3$): δ 7.69 (dd, J = 6.0, 1.2 Hz, 1H), 7.47-7.44 (m, 1H), 7.29 (t, J = 6.0 Hz, 2H), 7.24 (d, J = 5.2 Hz, 2H), 7.19 (t, J = 5.6 Hz, 2H), 7.02 (td, J = 6.4, 0.8 Hz, 1H), 6.96 (d, J = 6.8 Hz, 1H), 3.88 (s, 3H), 3.31 (t, J = 6.4 Hz, 2H), 3.02 (t, J = 6.4 Hz, 2H); ^{13}C NMR (100 MHz, $CDCl_3$): δ 201.7, 158.5, 141.7, 133.4, 130.3, 128.44, 128.37, 128.3, 125.9, 120.6, 111.5, 55.5, 45.4, 30.5.

3-(4-Methoxyphenyl)-1-(m-tolyl)propan-1-one 3g:[4]

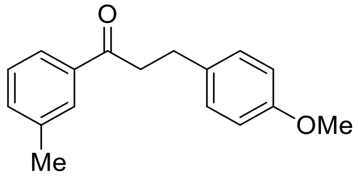

It was obtained as a yellow oil in 85% yield (80 mg). ^{1}H NMR (400 MHz, $CDCl_3$): δ 7.75 (d, J = 9.2 Hz, 2H), 7.38-7.31 (m, 2H), 7.17 (d, J = 8.8 Hz, 2H), 6.84 (d, J = 8.8 Hz, 2H), 3.79 (s, 3H), 3.26 (t, J = 7.2 Hz, 2H), 3.01 (t, J = 7.2 Hz, 2H), 2.40 (s, 3H); ^{13}C NMR (100 MHz, $CDCl_3$): δ 199.4, 157.9, 138.2, 136.4, 133.7, 133.3, 129.3, 128.5, 128.3, 125.1, 113.8, 55.1, 40.6, 29.2, 21.2.

3-(4-Methoxyphenyl)-1-(p-tolyl)propan-1-one 3h:[4]

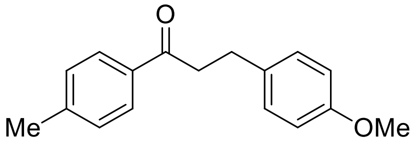

It was obtained as a colourless solid in 85% yield (63 mg). ^{1}H NMR (400 MHz, $CDCl_3$): δ 7.86 (d, J = 8.4 Hz, 2H), 7.25 (d, J = 8.0 Hz, 2H), 7.17 (d, J = 8.4 Hz, 2H), 6.84 (d, J = 8.8 Hz, 2H), 3.79 (s, 3H),

3.24 (t, J = 7.2 Hz, 2H), 3.00 (t, J = 7.2 Hz, 2H), 2.41 (s, 3H); ^{13}C NMR (100 MHz, $CDCl_3$): δ 199.0, 157.9, 143.8, 134.4, 133.4, 129.3, 129.2, 128.1, 113.9, 55.2, 40.6, 29.3, 21.6.

1,3-Bis(4-methoxyphenyl)propan-1-one 3i:[5]

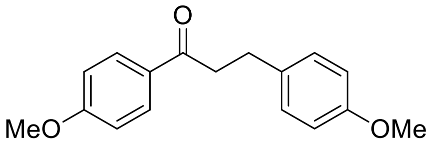

It was obtained as a colourless solid in 85% yield (63 mg). ^{1}H NMR (400 MHz, $CDCl_3$): δ 7.94 (d, J = 9.2 Hz, 2H), 7.17 (d, J = 8.8 Hz, 2H), 6.92 (d, J = 9.2 Hz, 2H), 6.84 (d, J = 8.8 Hz, 2H), 3.86 (s, 3H), 3.79 (s, 3H), 3.21 (t, J = 6.8 Hz, 2H), 3.00 (t, J = 7.2 Hz, 2H); ^{13}C NMR (100 MHz, $CDCl_3$): δ 198.0, 163.4, 157.9, 133.4, 130.3, 130.0, 129.3, 113.9, 113.7, 55.4, 55.2, 40.4, 29.5.

1-(4-Chlorophenyl)-3-phenylpropan-1-one 3j:[1]

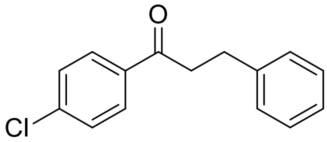

It was obtained as a colourless solid in 85% yield (63 mg). ^{1}H NMR (400 MHz, $CDCl_3$): δ 7.89 (d, J = 8.4 Hz, 2H), 7.42 (d, J = 8.8 Hz, 2H), 7.32-7.21 (m, 5H), 3.27 (t, J = 7.2 Hz, 2H), 3.06 (t, J = 7.6 Hz, 2H); ^{13}C NMR (100 MHz, $CDCl_3$): δ 198.0, 141.0, 139.5, 135.1, 129.4, 128.9, 128.6, 128.4, 126.2, 40.4, 30.0.

1-(4-Chlorophenyl)-3-(4-methoxyphenyl)propan-1-one 3k:[4]

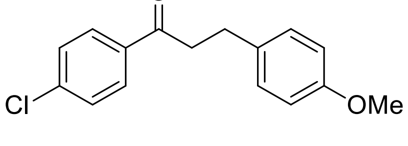

It was obtained as a colourless solid in 85% yield (63 mg). ^{1}H NMR (400 MHz, $CDCl_3$): δ 7.88 (d, J = 8.4 Hz, 2H), 7.42 (J = 8.4 Hz, 2H), 7.16 (d, J = 8.4 Hz, 2H), 6.84 (d, J = 8.8 Hz, 2H), 3.79 (s, 3H), 3.23 (t, J = 7.2 Hz, 2H), 3.00 (t, J = 7.6 Hz, 2H); ^{13}C NMR (100 MHz, $CDCl_3$): δ 198.1, 158.0, 139.4, 135.2, 133.0, 129.4, 129.3, 128.9, 113.9, 55.3, 40.7, 29.2.

3-(Furan-2-yl)-1-phenylpropan-1-one 3n:[1]

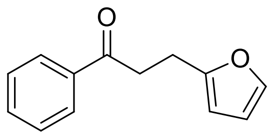

It was obtained as a colourless solid in 85% yield (63 mg). ^{1}H NMR (400 MHz, $CDCl_3$): δ 7.98 (d, J = 7.2 Hz, 2H), 7.57 (t, J = 7.2 Hz, 1H), 7.46 (t, J = 7.2 Hz, 2H), 7.32-7.31 (m, 1H), 6.29 (dd, J = 3.2, 2.0 Hz, 1H), 6.05 (dd, J = 3.2, 0.8 Hz, 1H), 3.34 (t, J = 7.2 Hz, 2H), 3.10 (t, J = 7.2 Hz, 2H); ^{13}C NMR (100 MHz, $CDCl_3$): δ 198.6, 154.8, 141.1, 136.7, 133.1, 128.6, 128.0, 110.2, 105.3, 36.9, 22.5.

3-(Furan-2-yl)-1-(p-tolyl)propan-1-one 3o:[6]

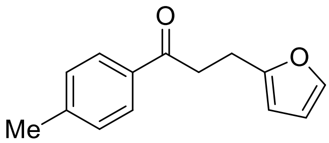

It was obtained as a colourless solid in 85% yield (63 mg). ^{1}H NMR (400 MHz, $CDCl_3$): δ 7.87 (d, J = 6.8 Hz, 2H), 7.31 (dd, J = 1.6, 0.6 Hz, 1H), 7.27-7.25 (m, 2H), 6.28 (dd, J = 2.4, 1.6 Hz, 1H), 6.04 (dd, J = 2.4, 0.4 Hz, 1H), 3.08 (t, J = 6.4 Hz, 2H), 3.31 (t, J = 6.4 Hz, 2H) 2.41 (s, 3H); ^{13}C NMR (100 MHz, $CDCl_3$): δ 198.3, 154.9, 143.9, 141.0, 134.3, 129.3, 128.1, 110.2, 105.2, 36.8, 22.6, 21.6.

3-(furan-2-yl)-1-(m-tolyl)propan-1-one 3p:

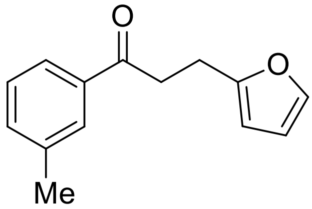

It was obtained as yellow oil in 57% yield (45 mg). IR (cm-1): 2914, 1716, 1680, 1603, 148, 1160, 1257, 1044, 1018, 999, 907, 833, 786, 127, 688, 647. ^{1}H NMR (400 MHz, $CDCl_3$): δ 7.78-7.76 (m, 2H), 7.39-7.33 (m, 2H), 7.31 (dd, J = 1.2, 0.4 Hz, 1H), 6.28 (dd, J = 2.4, 1.6 Hz, 1H), 6.05 (dd, J = 2.4, 0.4 Hz, 1H), 3.32 (t, J = 6.4 Hz, 2H), 3.09 (t, J = 6.4 Hz, 2H), 2.40 (s, 3H); ^{13}C NMR (100 MHz, $CDCl_3$): δ 198.9, 154.8, 141.0, 138.4, 136.7, 133,9, 128.5, 128.4, 125.2, 110.2, 105.3, 36.9, 22.5, 21.3. HRMS (ESI): m/z calcd for $C_{14}H_{14}O_2$ $[M+H]^+$ 215.1067; found 215.1069.

1-Phenyl-3-(pyridin-2-yl)propan-1-one 3r:[3]

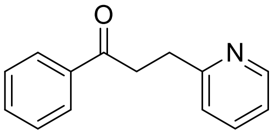

It was obtained as a colourless solid in 85% yield (63 mg). ^{1}H NMR (400 MHz, $CDCl_3$): δ 8.50 (d, J = 3.6 Hz, 1H), 7.98 (d, J = 6.0 Hz, 2H), 7.57 (t, J = 6.0 Hz, 1H), 7.52 (t, J = 6.0 Hz, 1H), 7.43 (t, J = 6.0 Hz, 2H), 7.24 (d, J = 6.4 Hz, 1H), 7.09 (t, J = 5.2 Hz, 1H), 3.50 (t, J = 6.0 Hz, 2H), 3.23 (t, J = 6.0 Hz, 2H); ^{13}C NMR (100 MHz, $CDCl_3$): δ 199.2, 160.6, 149.1, 136.8, 136.3, 132.9, 128.5, 128.0, 123.3, 121.2, 37.7, 32.0.

3-(pyridin-2-yl)-1-(p-tolyl)propan-1-one 3s:[7]

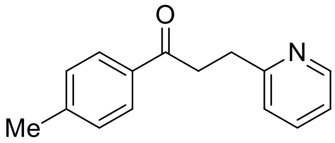

It was obtained as a colourless solid in 85% yield (63 mg). ^{1}H NMR (400 MHz, $CDCl_3$): δ 8.51 (dd, J = 4.8, 0.8 Hz, 1H), 7.89 (d, J = 8.0 Hz, 2H), 7.59 (td, J = 7.6, 2.0 Hz, 1H), 7.27-7.23 (m, 3H), 7.12-7.09 (m, 1H), 3.48 (t, J = 7.2 Hz, 2H), 3.23 (t, J = 7.2 Hz, 2H), 2.40 (s, 3H); ^{13}C NMR (100 MHz, $CDCl_3$): δ 198.9, 160.8, 149.2, 143.7, 129.2, 128.2, 123.4, 121.2, 37.7, 32.1, 21.6.

2-(Benzyloxy)-8-bromoquinoline 11[8,9]**:**

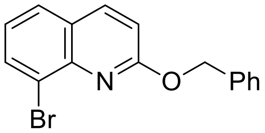

It is prepared according to the literature procedure. It was obtained as a colourless liquid in 82% yield (0.8 g). ^{1}H NMR (400 MHz, $CDCl_3$): δ 7.91-7.86 (m, 2H), 7.59 (dd, J = 8.0, 1.2 Hz, 1H), 7.55-7.53 (m, 2H), 7.33-7.29 (m, 2H), 7.27-7.25 (m, 1H), 7.18-7.13 (m, 1H), 6.90 (d, J = 8.8 Hz, 1H), 5.57 (s, 2H).

4. Synthesis of Pd complexes

Synthesis of Ligand L-1

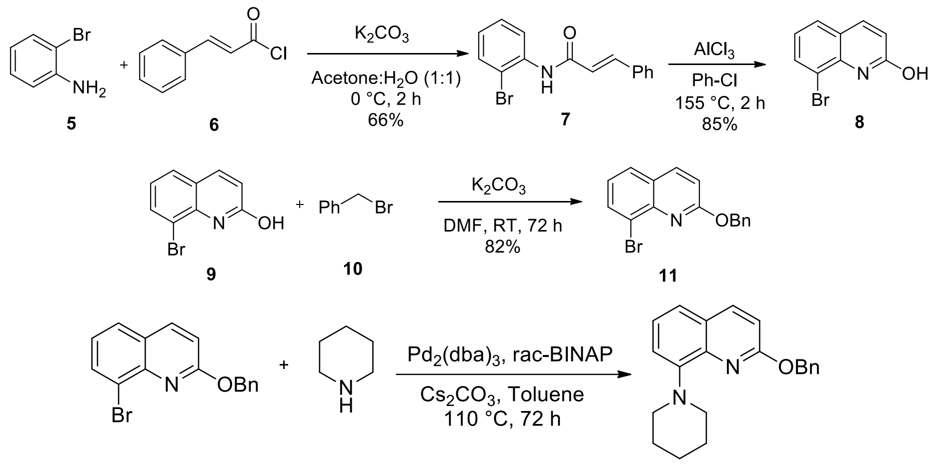

Following literature procedures, 2-(benzyloxy)-8-bromoquinoline compound **11** was obtained in 82% yield starting from 2-bromoaniline and *trans*-cinnamoyl chloride. To a solution of 2-(benzyloxy)-8-bromoquinoline and piperidine in toluene, $Pd_2(dba)_3$ (10 mol%), rac-BINAP (10 mol%) and Cs_2CO_3 (2 equiv.) were added at room temperature. Air from the reaction mixture was evacuated and refilled with nitrogen and the same process was repeated for three times at room temperature. Then, the reaction mixture was stirred at 110 °C for 72 h under N_2 atmosphere. After cooling down to RT, water was added to the reaction mixture, and then compound was extracted with ethyl acetate, dried over $MgSO_4$ and concentrated under reduced pressure. The crude was purified by column chromatography (silica gel, petroleum ether/ethyl acetate) to get furnish pure 2-(benzyloxy)-8-(piperidin-1-yl)quinolone in 71% yield as yellow liquid. IR (cm-1): 2928, 1612, 1573, 1503, 1461, 1261, 1241, 1226, 1087, 1063, 1016, 992, 931, 858, 828, 728, 693, 622. ^{1}H NMR (400 MHz, $CDCl_3$): δ 7.99 (dd, J = 8.8 Hz, 1H), 7.52 (d, J = 7.2 Hz, 2H), 7.42-7.38 (m, 2H), 7.36-7.29 (m, 3H), 7.12 (d, J = 7.2 Hz, 1H), 6.96 (d, J = 8.4 Hz, 1H), 5.60 (s, 2H), 3.31 (br s, 4H), 1.88 (t, J = 5.2 Hz, 4H), 1.68-1.66 (m, 2H); ^{13}C NMR (100 MHz, $CDCl_3$): δ 159.9, 148.7, 140.1, 139.7, 137.4, 128.0, 127.8, 126.3, 124.3, 120.9, 116.7, 112.4, 67.3, 53.2, 26.5, 24.7. HRMS (ESI): m/z calcd for $C_{21}H_{22}N_2O$ $[M+H]^+$ 319.1805; found 319.1804.

8-(Piperidin-1-yl)quinolin-2-ol (L1)

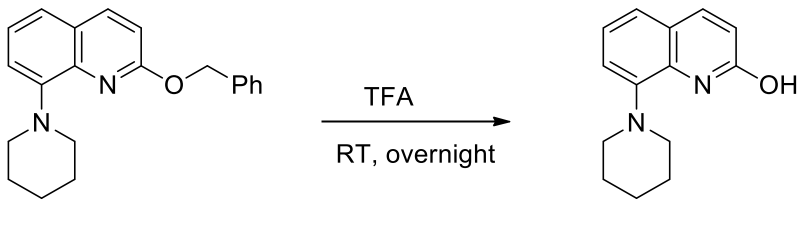

A solution of 2-(benzyloxy)-8-(piperidin-1-yl)quinolone (0.2 mmol) in TFA (3 mL) was stirred for overnight at room temperature. The reaction mixture was neutralized using saturated $NaHCO_3$ solution. The compound was extracted with chloroform, dried over $MgSO_4$ and concentrated under reduced pressure. The crude was purified by column chromatography (silica gel, dichloromethane/methanol) to get pure 8-(Piperidin-1-yl)quinolin-2-ol. It was obtained as a colourless solid in 69% yield (0.8 g). IR (cm-1): 3356, 2927, 2806, 1648, 1602, 1567, 1462, 1395, 1335, 1271, 1257, 1035, 1005, 975, 863, 849, 832, 747, 715, 667, 620. ^1H NMR (400 MHz, $CDCl_3$): δ 9.50 (br s, 1H), 7.74 (d, J = 9.2 Hz, 1H), 7.32 (d, J = 7.6 Hz, 2H), 7.16 (dd, J = 8.0, 7.2 Hz, 1H), 6.66 (d, J = 9.6 Hz, 1H), 2.86 (br s, 4H), 1.78 (t, J = 5.6 Hz, 6H); ^{13}C NMR (100 MHz, $CDCl_3$): δ 162.5, 140.7, 133.6, 123.7, 122.4, 122.23, 122.18, 120.1, 54.1, 26.5, 24.0. HRMS (ESI): m/z calcd for $C_{14}H_{16}N_2O$ $[M+H]^+$ 229.1341 Found 229.1346.

Synthesis of Ligand L-2

2-(Benzyloxy)-6-bromopyridine 15:[11]

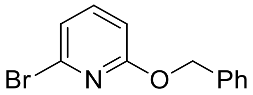

It is prepared according to the literature procedure. It was obtained as a colourless solid in 58% yield (1.2 g). ^1H NMR (400 MHz, $CDCl_3$): δ 7.50-7.40 (m, 6H), 7.10 (d, J = 7.2 Hz, 1H), 6.77 (d, J = 8.4 Hz, 1H), 5.39 (s, 2H).

6-(benzyloxy)picolinaldehyde 16[12]:

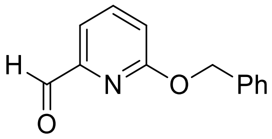

It is prepared according to the literature procedure. It was obtained as a colourless liquid in 93% yield (1.2 g). ^1H NMR (400 MHz, $CDCl_3$): δ 9.98 (s, 1H), 7.75 (t, J = 7.6 Hz, 1H), 7.58 (d, J = 7.2 Hz, 1H), 7.50 (d, J = 7.2 Hz, 2H), 7.42-7.32 (m, 3H), 7.04 (d, J = 8.4 Hz, 1H), 5.49 (s, 2H).

2-(Benzyloxy)-6-(piperidin-1-ylmethyl)pyridine:

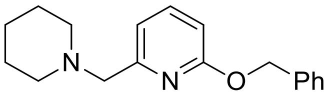

6-(Benzyloxy)picolinaldehyde (1 equiv.) was added to a solution of piperidine (1.2 equiv.) in dry methanol at room temperature under nitrogen atmosphere. The reaction mixture was allowed to stir at

room temperature for overnight. 1.2 equiv. of $NaBH_4$ was added to the reaction mixture at 0 °C in the two batches in 1 h interval time. After 2 h, the reaction mixture was neutralized with saturated NH_4Cl solution and compound was extracted using dichloromethane and dried over $MgSO_4$. Dichloromethane was evaporated in reduced pressure and the crude was purified by column chromatography (silica gel, dichloromethane/methanol) to furnish pure 2-(Benzyloxy)-6-(piperidin-1-ylmethyl)pyridine as a yellow liquid in 94% yield (0.75 g). IR (cm^{-1}): 2932, 1596, 1573, 1336, 1305, 1259, 1148, 1112, 1078, 1037, 1016, 987, 962, 799, 781, 695. ^1H NMR (400 MHz, $CDCl_3$): δ 7.54 (dd, J = 8.4, 7.6 Hz, 1H), 7.47-7.45 (m, 2H), 7.38-7.34 (m, 2H), 7.32-7.28 (m, 1H), 7.01 (d, J = 7.2 Hz, 1H), 6.66 (d, J = 8.0 Hz, 1H), 5.38 (s, 2H), 3.58 (s, 2H), 2.48 (s, 4H), 1.63-1.58 (m, 4H), 1.45-1.44 (m, 2H); ^{13}C NMR (100 MHz, $CDCl_3$): δ 162.9, 138.8, 137.6, 128.3, 128.0, 127.7, 115.9, 108.9, 67.4, 64.7, 54.6, 25.9, 24.2. HRMS (ESI): m/z calcd for $C_{18}H_{22}N_2O$ $[M+H]^+$ 283.1810 Found 283.1813.

6-(Piperidin-1-ylmethyl)pyridin-2-ol (L2):

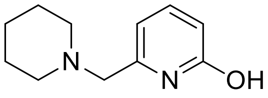

A solution of 2-(Benzyloxy)-6-(piperidin-1-ylmethyl)pyridine (0.2 mmol) in TFA (3 mL) was stirred for overnight at room temperature. The reaction mixture was neutralized using saturated $NaHCO_3$ solution. The compound was extracted with chloroform, dried over $MgSO_4$ and concentrated under reduced pressure. The crude was purified by column chromatography (silica gel, dichloromethane/methanol) to get pure 6-(Piperidin-1-ylmethyl)pyridin-2-ol (**L2**) as a brown solid in 72% yield (0.75 g). IR (cm^{-1}): 2933, 2847, 1653, 1614, 1545, 1459, 1428, 1309, 1177, 1155, 1010, 990, 966, 882, 858, 807, 766, 756, 733, 687. ^1H NMR (400 MHz, $CDCl_3$): δ 10.02 (br s, 1H), 7.30 (dd, J = 9.2, 6.4 Hz, 1H), 6.41 (d, J = 9.2 Hz, 1H), 6.01 (dd, J = 6.8, 0.8 Hz, 1H), 3.33 (s, 2H), 2.40 (s, 4H), 1.60-1.55 (m, 4H), 1.45-1.43 (m, 2H); ^{13}C NMR (100 MHz, $CDCl_3$): δ 163.6, 145.1, 141.0, 119.1, 104.1, 59.2, 54.4, 25.8, 23.8. HRMS (ESI): m/z calcd for $C_{11}H_{16}N_2O$ $[M+Na]^+$ 215.1160 Found 215.1166.

<u>Synthesis of Ligand L-3</u>

N-((6-(benzyloxy)pyridin-2-yl)methyl)-N-ethylethanamine:

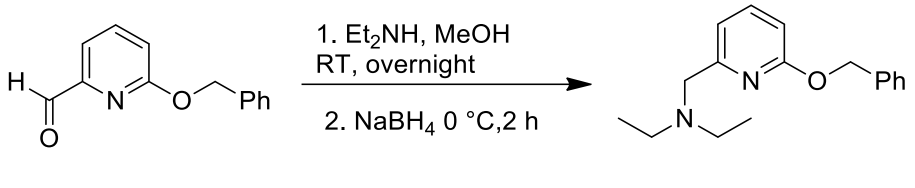

6-(Benzyloxy)picolinaldehyde (1 equiv.) was added to a solution of diethyl amine (1.2 equiv.) in dry methanol at room temperature under nitrogen atmosphere. The reaction mixture was allowed to stir at

room temperature for overnight. 1.2 equiv. of $NaBH_4$ was added to the reaction mixture at 0 °C in the two batches in 1 h interval time. After 2 h, the reaction mixture was neutralized with saturated NH_4Cl solution and compound was extracted using dichloromethane and dried over $MgSO_4$. Dichloromethane was evaporated in reduced pressure and the crude was purified by column chromatography (silica gel, dichloromethane/methanol) to furnish pure N-((6-(benzyloxy)pyridin-2-yl)methyl)-N-ethylethanamine. [13] It was obtained as a colourless liquid in 36% yield (0.8 g). IR (cm^{-1}): 2967, 1596, 1574, 1445, 1265, 1221, 1164, 1071, 1016, 988, 795, 730, 695. ^1H NMR (400 MHz, $CDCl_3$): δ 7.54 (t, J = 7.2 Hz, 1H), 7.47 (d, J = 7.2 Hz, 2H), 7.39-7.35 (m, 2H), 7.33-7.29 (m, 1H), 7.03 (d, J = 7.2 Hz, 1H), 6.65 (d, J = 8.0 Hz, 1H), 5.38 (s, 2H), 3.68 (s, 2H), 2.60 (q, J = 6.8 Hz, 4H), 1.09 (t, J = 6.8 Hz, 6H); ^{13}C NMR (100 MHz, $CDCl_3$): δ 162.8, 157.7, 138.8, 137.6, 128.3, 128.0, 127.6, 126.5, 115.6, 108.6, 67.3, 58.5, 47.3, 12.0. HRMS (ESI): m/z calcd for $C_{17}H_{22}N_2O$ $[M+H]^+$ 271.1805 Found 271.1809.

6-((Diethylamino)methyl)pyridin-2-ol (L3):

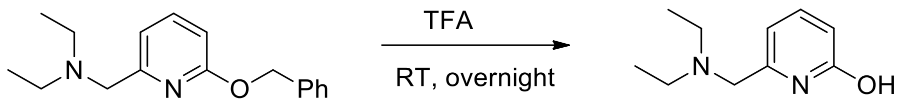

A solution of N-((6-(benzyloxy)pyridin-2-yl)methyl)-N-ethylethanamine (0.2 mmol) in TFA (3 mL) was stirred for overnight at room temperature.[10] The reaction mixture was neutralized using saturated $NaHCO_3$ solution. The compound was extracted with chloroform, dried over $MgSO_4$ and concentrated under reduced pressure. The crude was purified by column chromatography (silica gel, dichloromethane/methanol) to get pure 6-((diethylamino)methyl)pyridin-2-ol. It was obtained as a colourless liquid in 61% yield (0.8 g). IR (cm^{-1}): 2971, 1648, 1610, 1449, 1198, 1176, 1126, 798, 718, 557. ^1H NMR (400 MHz, $CDCl_3$): δ 10.26 (br s, 1H), 7.27 (dd, J =9.2. 6.8 Hz, 1H), 6.34 (dd, J = 9.2, 0.8 Hz, 1H), 6.00 (dd, J = 6.8, 1.2 Hz, 1H), 3.41 (s, 2H), 2.49 (q, J = 7.2 Hz, 4H), 0.96 (t, J = 7.2 Hz, 6H); ^{13}C NMR (100 MHz, $CDCl_3$): δ 163.5, 146.4, 141.1, 118.5, 103.5, 53.9, 47.2, 11.7. HRMS (ESI): m/z calcd for $C_{10}H_{16}N_2O$ $[M+H]^+$ 181.1341 Found 181.1345.

Synthesis of Ligand L4

8-(Piperidin-1-yl)quinoline (**L-4**):

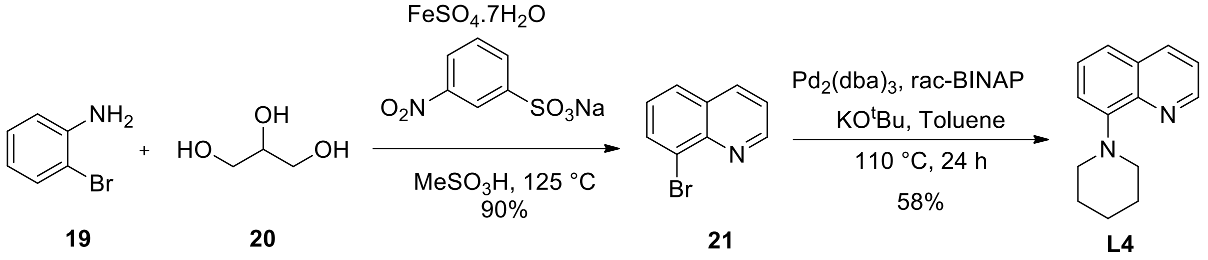

Scheme 6: Preparation of ligand 4 (**L4**)

The deoxy-ligand **L4** was synthesized according to the reported literature procedure.[15] The key starting material 8-bromoquinoline **21** was prepared by the Skraup reaction.[15] Next, the Pd-catalyzed coupling of **21** with piperidine affording the required deoxy-ligand **L4** in 58% yield. ^{1}H NMR (400 MHz, $CDCl_3$): δ 8.90 (dd, J = 1,9, 4.1 Hz, 1H), 8.08 (m, 1H), 7.33-7.41 (m, 3H), 7.14(m, 1H), 3.32 (m, 4H), 1.91 (m, 4H), 1.66(m, 2H), 0.89-1.2 (impurities).

Synthesis of 1-[Pd]

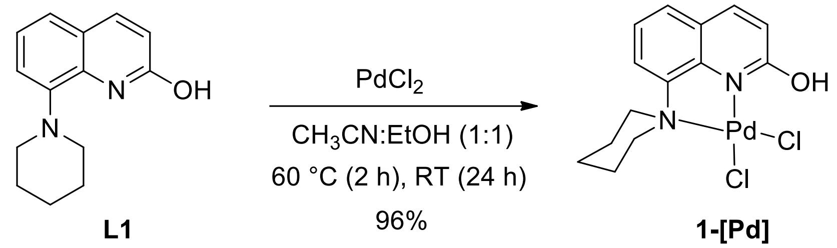

The **1-[Pd]** complex was obtained in 96% yield by treating Ligand L1 (1.2 eq.) with $PdCl_2$(1 eq.) in CH_3CN: EtOH (1:1) mixture for 2 h at 60 °C and further stirring for 24 h at room temperature. Petroleum ether was added into the reaction mixture and the solution was decanted. The product was further washed with petroleum ether and dried under vacuum. X-ray quality crystals were grown from the mixture of toluene and acetonitrile. ^{1}H NMR (400 MHz, $CDCl_3$): δ 12.78 (br s, 1H), 8.19 (d, J = 8.2 Hz, 1H), 8.12 (d, J = 8.8 Hz, 1H), 7.70 (d, J = 7.8 Hz, 1H), 7.44 (t, J = 8 Hz, 1H), 6.93 (d, J = 8.99 Hz, 1H), 4.99 (dt, J = 5, 8.8 Hz, 2H), 3,47 (m, 2H), 1.65-2.2 (m, 6H), 1,60 (impurity of water) ; ^{13}C NMR (100 MHz, $CDCl_3$): δ 167.52, 149.20, 144.52, 141.40, 128.58, 126.59, 126.20, 125.08, 116.98, 60.78, 22.94, 21.49. HRMS (ESI): m/z calcd for $C_{16}H_{19}N_3OPdCl^+$ [M-Cl+CH_3CN]$^+$ 410.0255 Found 410.0252

Synthesis of 2-[Pd]

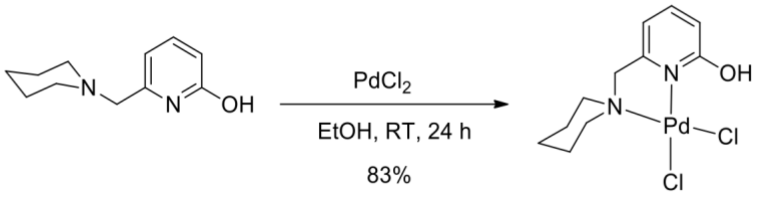

The **2-[Pd]** complex was obtained in 96% yield by treating Ligand 2(1.2 eq.) with $PdCl_2$(1 eq.) in CH_3CN:EtOH (1:1) mixture for 2 h at 60 °C and 24 h at room temperature. Petroleum ether was added into the reaction mixture and the solution was decanted. The product was further washed with petroleum ether and dried under vacuum. X-ray quality crystals were grown from the mixture of toluene and acetonitrile. ^{1}H NMR (400 MHz, CD_2Cl_2): δ 11.62 (br s, 1H), 7.74 (t , J = 8.8 Hz, 1H), 6.95 (d , J = 7.5 Hz, 1H), 6.72 (d, J = 8.4 Hz, 1H), 4.30 (s, 2H), 3.77 (m, 2H), 3.07 (d, J = 13.8 Hz, 2H), 1.76 (m, 3H), 1.50 (m, 3H), impurities: 2.36, 1.98 and 1,26; ^{13}C NMR (100 MHz, $CDCl_3$): δ 167.89, 155.41, 142.47, 113.83, 113.72, 63.99, 58.96, 23.35, 20.58. HRMS (ESI): m/z calcd for $C_{13}H_{19}N_3OPdCl^+$ [M-Cl+CH_3CN]$^+$ 374.0251 Found 374.0255.

Synthesis of 3-[Pd]

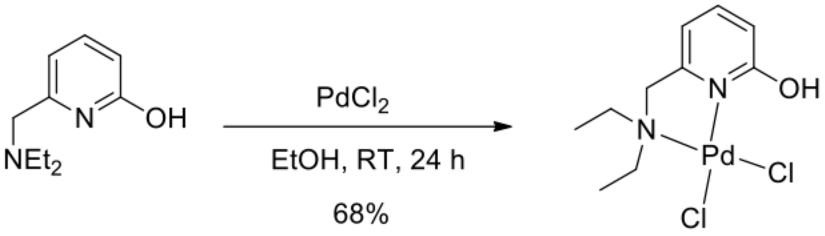

The **3-[Pd]** complex was obtained in 96% yield by treating Ligand 3(1.2 eq.) with $PdCl_2$(1 eq.) in CH_3CN:EtOH (1:1) mixture for 2 h at 60 °C and 24 h at room temperature. Petroleum ether was added into the reaction mixture and the solution was decanted. The product was further washed with petroleum ether and dried under vacuum. X-ray quality crystals were grown from the mixture of toluene and acetonitrile ^{1}H NMR (400 MHz, $CDCl_3$): δ 11.83 (br s, 1H), 7.69 (m,1H), 6.88 (d, J = 7.2 Hz, 1H), 6.71 (d, J = 8.6 Hz, 1H), 4.01 (s, 2H), 3.16 (m, 2H), 2.57 (m, 2H), 1.61 (t, J = 7.1 Hz, 6H), 1.71 and 2.01 (impurities) ; ^{13}C NMR (100 MHz, $CDCl_3$): δ 167.82, 156.51, 141.89, 113.44, 112.41, 65.34, 56.04, 12.33. HRMS (ESI): m/z calcd for $C_{12}H_{19}N_3OPdCl^+$ [M-Cl+CH_3CN]$^+$ 362.0251 Found 362.0252.

Synthesis of 4-[Pd]

The **4-[Pd]** complex was obtained in 78% yield by treating Ligand 4 (**L4**) with $PdCl_2$ in CH_3CN:EtOH (1:1) mixture for 2 h at 60 °C and 24 h at room temperature (Scheme 7).

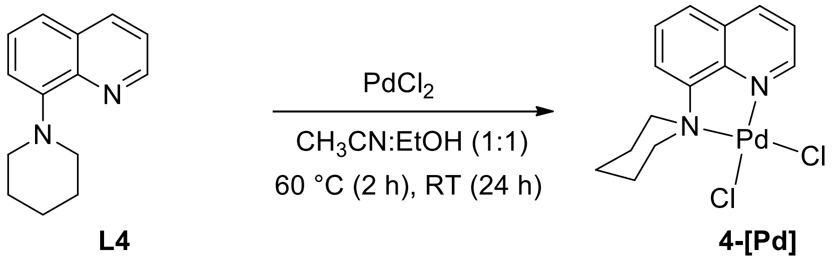

Scheme 7: Synthesis of **4-[Pd]** complex

The **4-[Pd]** complex was obtained in 96% yield by treating Ligand 4(1.2 eq.) with $PdCl_2$(1 eq.) in CH_3CN:EtOH (1:1) mixture for 2 h at 60 °C and 24 h at room temperature. Petroleum ether was added into the reaction mixture and the solution was decanted. The product was further washed with petroleum ether and dried under vacuum. X-ray quality crystals were grown from the mixture of toluene and acetonitrile. ^{1}H NMR (400 MHz, $CDCl_3$): 9.58 (m, 1H), 8.43 (m, 2H), 7.93 (d, *J* = 8 Hz, 1H), 7.70 (t, *J* = 8.1 Hz, 1H), 7.62 (m, 1H), 5.00 (dt, *J* = 6, 9.2 Hz, 2H), 3,51 (m, 2H), 1.67-2.2 (m, 6H), 1,58, 1.25 and 0.87 (impurities); ^{13}C NMR (100 MHz, $CDCl_3$): δ 152.07, 150.46, 147.06, 139.23, 13.80, 128.67, 128.07, 127.64, 122.98, 60.21, 22.72, 21.72. HRMS (ESI): m/z calcd for $C_{16}H_{19}N_3PdCl^+$ [M-Cl+CH_3CN]$^+$ 394.0302 Found 394.0300.

5. X-ray crystal structures

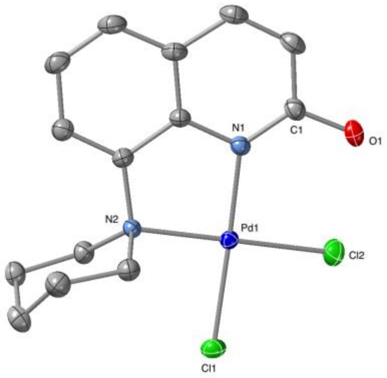

Figure S23. Molecular structure of 1-[Pd] at the 30 % probability level. Hydrogen atoms are omitted for clarity.

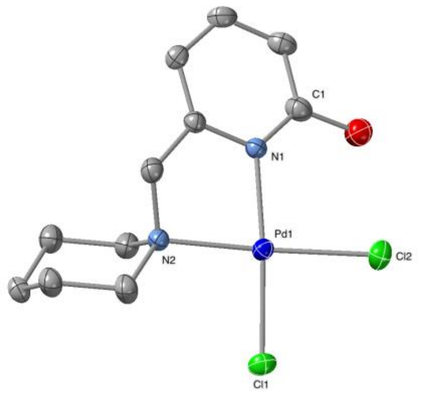

Figure S23. Molecular structure of 2-[Pd] at the 30 % probability level. Hydrogen atoms are omitted for clarity.

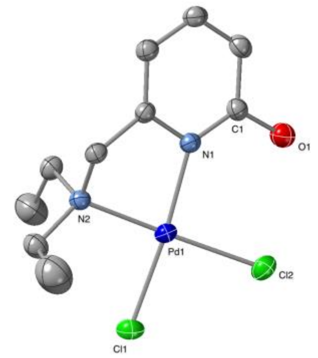

Figure S23. Molecular structure of 3-[Pd] at the 30 % probability level. Hydrogen atoms are omitted for clarity.

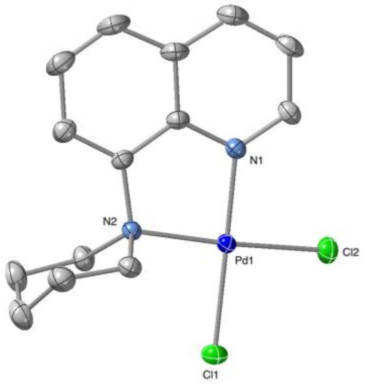

Figure S23. Molecular structure of 4-[Pd] at the 30 % probability level. Hydrogen atoms are omitted for clarity.

Table 1 Crystal data and structure refinement for the palladium complexes.

Identification code	1-[Pd]	2-[Pd]	3-[Pd]	4-[Pd]
Empirical formula	$C_{14}H_{16}Cl_2N_2OPd$	$C_{22}H_{32}Cl_4N_4O_2Pd_2$	$C_{10}H_{16}Cl_2N_2OPd$	$C_{14}H_{16}Cl_2N_2Pd_1$
Formula weight	405.59	739.11	357.55	389.59
Temperature/K	293.00	293.00	293.0	293
Crystal system	monoclinic	monoclinic	monoclinic	monoclinic
Space group	$P2_1/n$	$P2_1/n$	$P2_1/n$	$P2_1/n$
a/Å	10.1365(4)	10.0690(3)	7.89501(14)	7.0059(2)
b/Å	12.9777(4)	12.1280(2)	11.50931(18)	11.9229(4)
c/Å	12.0874(5)	22.1284(6)	15.1843(2)	17.5449(5)
α/°	90	90	90	90
β/°	110.7449(4)	97.898(2)	104.4210(18)	94.375(2)
γ/°	90	90	90	90
Volume/Å^3	1486.99(10)	2676.62(12)	1336.27(4)	1461.27(8)
Z	4	4	4	4
ρ_{calc}g/cm^3	1.812	1.834	1.777	1.771
μ/mm^{-1}	1.603	1.771	1.770	1.622
F(000)	808.0	1472.0	712.0	776.0
Radiation	MoKα (λ = 0.71069)	MoKα (λ = 0.71069)	MoKα (λ = 0.71069)	MoKα (λ =0.71069)
2Θ range for data collection/°	6.278 to 58.268	6.444 to 57.566	6.396 to 54.626	5.776 to 57.644
Index ranges	-12 ≤ h ≤ 13, -17 ≤ k ≤ 17, -16 ≤ l ≤ 15	-13 ≤ h ≤ 12, -15 ≤ k ≤ 15, -29 ≤ l ≤ 27	-10 ≤ h ≤ 10, -14 ≤ k ≤ 14, -19 ≤ l ≤ 19	-9 ≤ h ≤ 9, -15 ≤ k ≤ 15, -22 ≤ l ≤ 23
Reflections collected	9971	24069	2850	16291
Independent reflections	3535 [R_{int} = 0.0177, R_{sigma} = 0.0214]	6231 [R_{int} = 0.0409, R_{sigma} = 0.0366]	2850 [R_{int} = 0.0189, R_{sigma} = 0.0268]	3536 [R_{int} = 0.0464, R_{sigma} = 0.0416]
Data/restraints/parameters	3535/0/181	6231/0/315	2850/1/169	3536/0/172
Goodness-of-fit on F^2	0.831	1.40	1.111	1.059
Final R indexes [I>=2σ (I)]	R_1 = 0.0266, wR_2 = 0.0946	R_1 = 0.0349, wR_2 = 0.0366	R_1 = 0.0345, wR_2 = 0.0863	R_1 = 0.0367, wR_2 = 0.0654
Final R indexes [all data]	R_1 = 0.0317, wR_2 = 0.0143	R_1 = 0.0559, wR_2 = 0.0402	R_1 = 0.0429, wR_2 = 0.0924	R_1 = 0.0536, wR_2 = 0.0708
Largest diff. peak/hole / e Å^{-3}	0.44/-0.89	0.81/-0.60	1.39/-0.53	0.57/-0.41

CCDC	2209156	2104033	2104035	2108795

Table 2: Selected bond lengths and angles

Bond lengths (Å)	Pd-1	Pd-2	Pd-3	Pd-4
Pd1−N1	2.043(2)	2.065(3)	2.076(3)	2.015(2)
Pd1−N2	2.099(2)	2.085(3)	2.078(3)	2.115(2)
Pd1−Cl1	2.298(8)	2.2980(12)	2.3175(12)	2.3153(8)
Pd1−Cl2	2.311(9)	2.3061(12)	2.2853(12)	2.3148(8)
O1-C1	1.321(4)	1.325(5)	1.322(6)	-
Bond angles (deg):				
N1−Pd1-N2	83.24(9)	81.70(11)	81.64(13)	83.25(9)
N1−Pd1-Cl2	98.08(7)	96.09(9)	96.34(10)	94.21(8)
Cl1−Pd1-Cl2	85.36(3)	87.86(5)	88.05(5)	89.03(3)
Cl1−Pd1-N2	93.57(6)	94.75(8)	94.10(10)	93.99(7)

6. References

1. X.-N. Cao, X.-M. Wan, F.-L. Yang, K. Li, X.-Q. Hao, T. Shao, X. Zhu, M.-P. Song, *J. Org. Chem*. **2018**, *83*, 3657−3668.
2. M. PeÇa-Llpez, P. Piehl, S. Elangovan, H. Neumann, M. Beller, *Angew. Chem. Int. Ed*. **2016**, *55*, 14967–14971.
3. J. Das, M. Vellakkaran, D. Banerjee, *J. Org. Chem*. **2019**, *84*, 769−779.
4. X. Hu, H. Zhu, X. Sang, D. Wang, *Adv. Synth. Catal.* **2018**, *360*, 4293–4300.
5. Q. Xu, J. Chen, H. Tian, X. Yuan, S. Li, C. Zhou, J. Liu, *Angew. Chem. Int. Ed*. **2014**, *53*, 225–229.
6. M. K. Barman, A. Jana, B. Maji, *Adv. Synth. Catal*. **2018**, *360*, 3233–3238.
7. R. S. Rohokale, B. Koenig, D. D. Dhavale, *J. Org. Chem*. **2016**, *81*, 7121−7126.
8. L. D. Wickramasinghe, R. Zhou, R. Zong, P. Vo, K. J. Gagnon, R. P. Thummel, *J. Am. Chem. Soc*. **2015**, *137*, 13260–13263.
9. P. Bamborough, C.-w. Chung, R. C. Furze, P. Grandi, A.-M. Michon, R. J. Sheppard, H. Barnett, H. Diallo, D. P. Dixon, C. Douault, E. J. Jones, B. Karamshi, D. J. Mitchell, R. K. Prinjha, C. Rau, R. J. Watson, T. Werner, E. H. Demont, *J. Med. Chem*. **2015**, *58*, 6151−6178.
10. M. Akazome, S. Suzuki, Y. Shimizu, K. Henmi, K. Ogura, *J. Org. Chem*. **2000**, *65*, 6917-6921.
11. J.-N. Heo, Y. S. Song, B. T. Kim, *Tetrahedron Lett*. **2005**, *46*, 4621-4625.
12. M. Hatanaka, K. Takahashi, S. Nakamura, T. Mashino, *Bioorg. Med. Chem*. **2005**, *13*, 6763-6770.

13. H. A. Michaels, C. S. Murphy, R. J. Clark, M. W. Davidson, L. Zhu, *Inorg. Chem*. **2010**, *49*, 4278–4287.

7. Copy of ^{1}H and ^{13}C NMR spectra

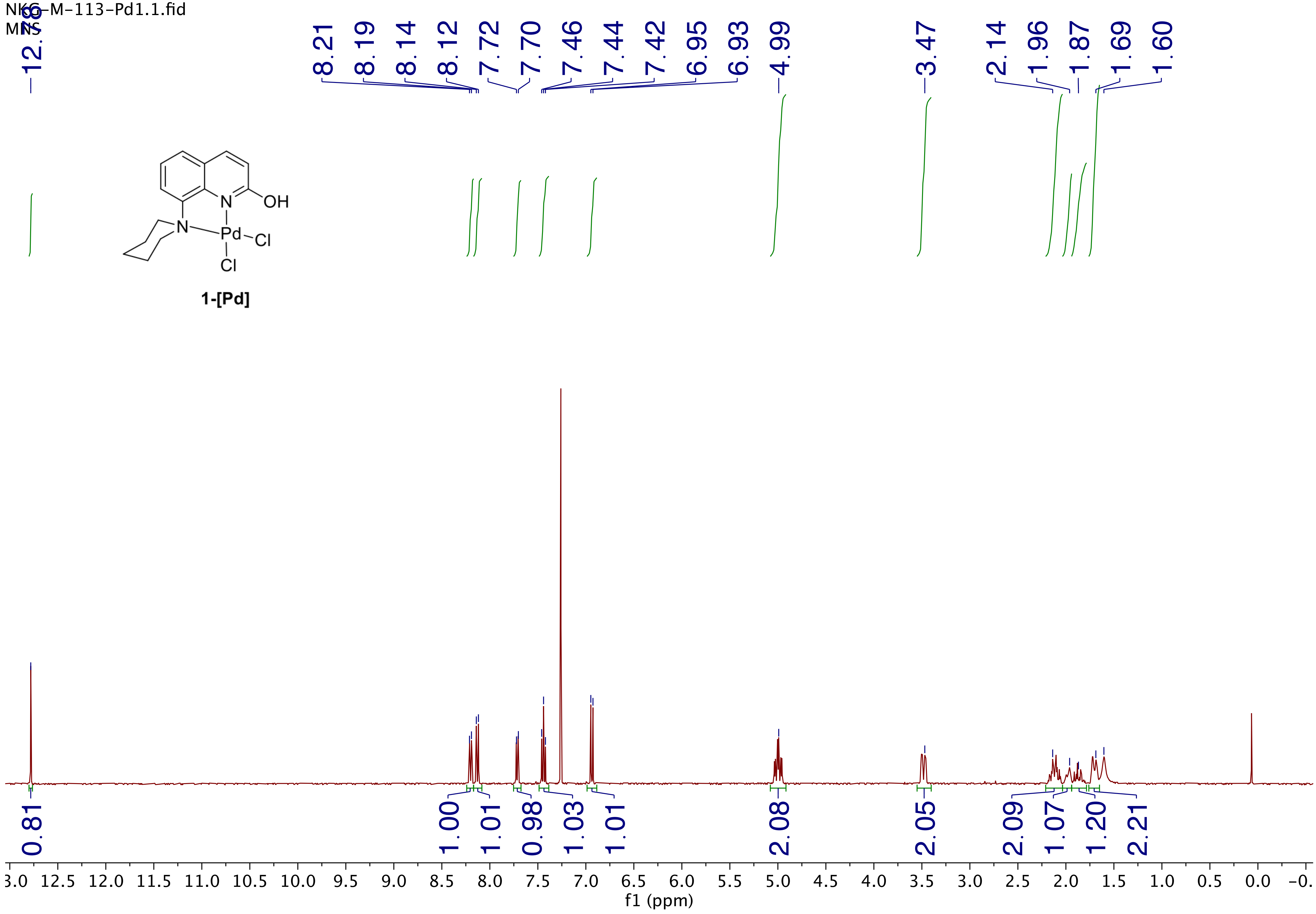
NKG-M-113-Pd1.1.fid
MNS
12.78
8.21 8.19 8.14 8.12 7.72 7.70 7.46 7.44 7.42 6.95 6.93
4.99
3.47
2.14 1.96 1.87 1.69 1.60
OH
N
Pd
Cl
Cl
1-[Pd]
0.81
1.00 1.01 0.98 1.03 1.01
2.08
2.05
2.09 1.07 1.20 2.21
13.0 12.5 12.0 11.5 11.0 10.5 10.0 9.5 9.0 8.5 8.0 7.5 7.0 6.5 6.0 5.5 5.0 4.5 4.0 3.5 3.0 2.5 2.0 1.5 1.0 0.5 0.0 -0.5
f1 (ppm)

NKG-M-113-Pd1.2.fid
MNS

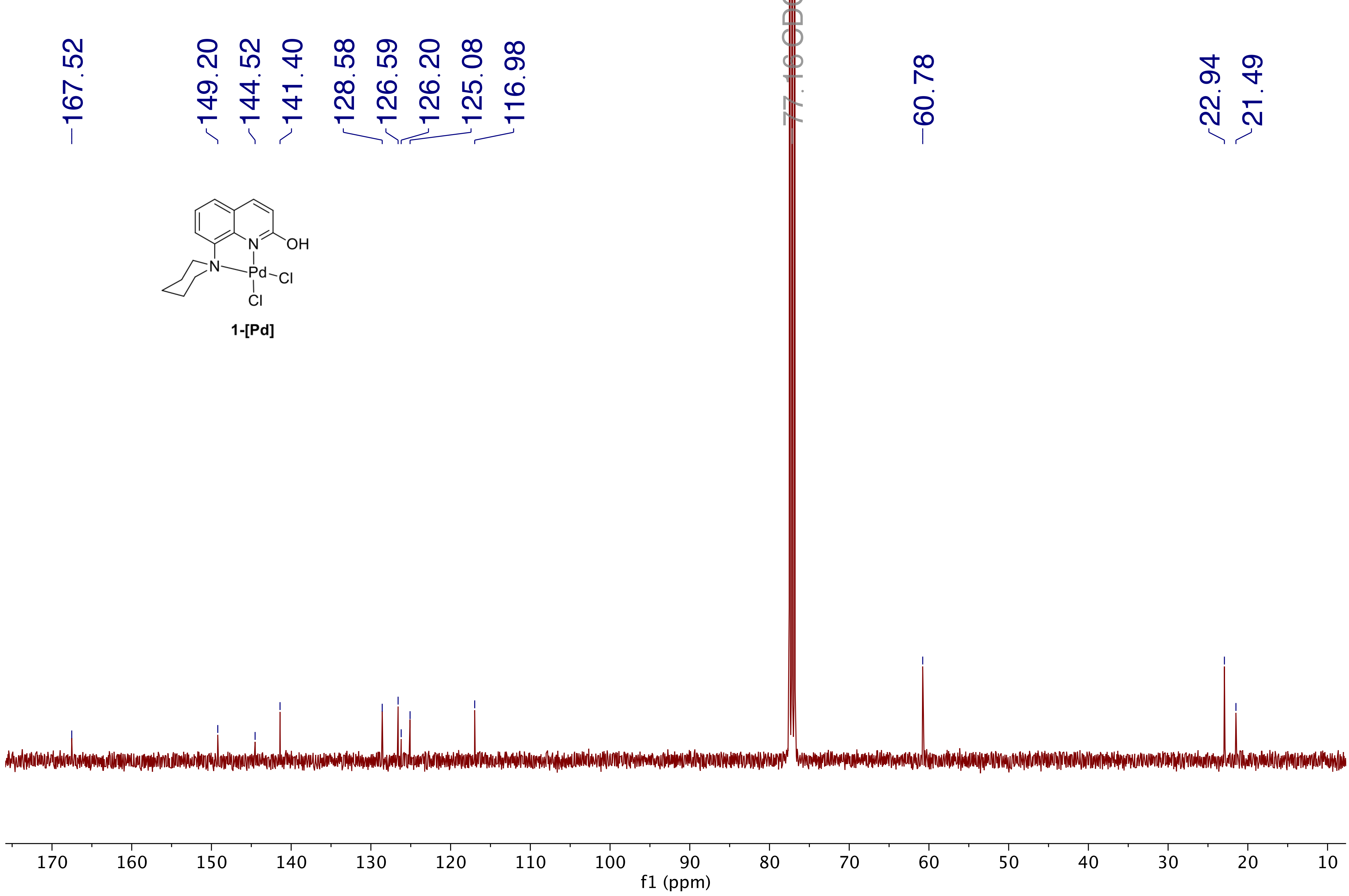

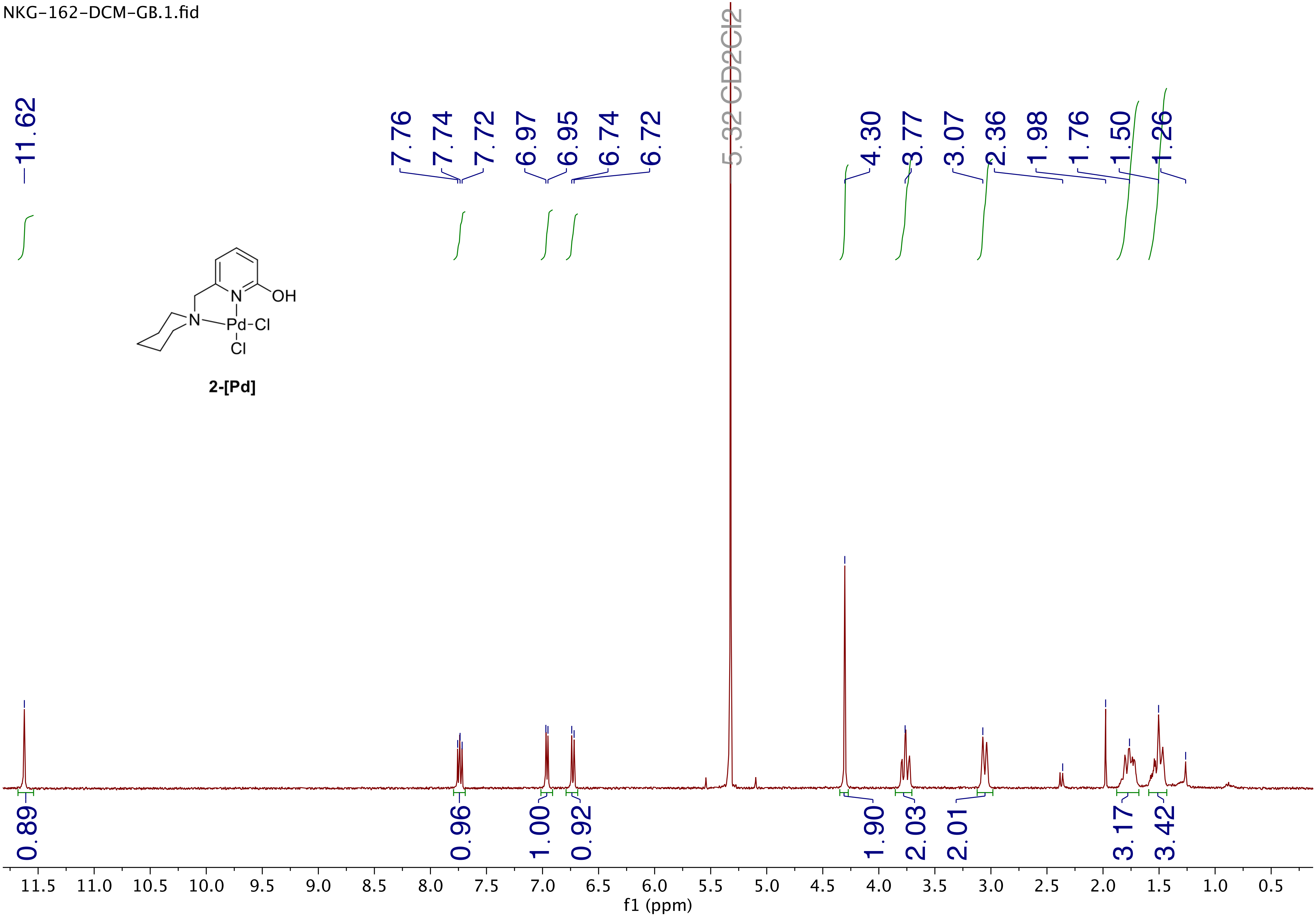
NKG-162-DCM-GB.1.fid
11.62
7.76
7.74
7.72
6.97
6.95
6.74
6.72
5.32 CD2Cl2
4.30
3.77
3.07
2.36
1.98
1.76
1.50
1.26
OH
N
N
Pd-Cl
Cl
2-[Pd]
0.89
0.96
1.00
0.92
1.90
2.03
2.01
3.17
3.42
11.5 11.0 10.5 10.0 9.5 9.0 8.5 8.0 7.5 7.0 6.5 6.0 5.5 5.0 4.5 4.0 3.5 3.0 2.5 2.0 1.5 1.0 0.5
f1 (ppm)

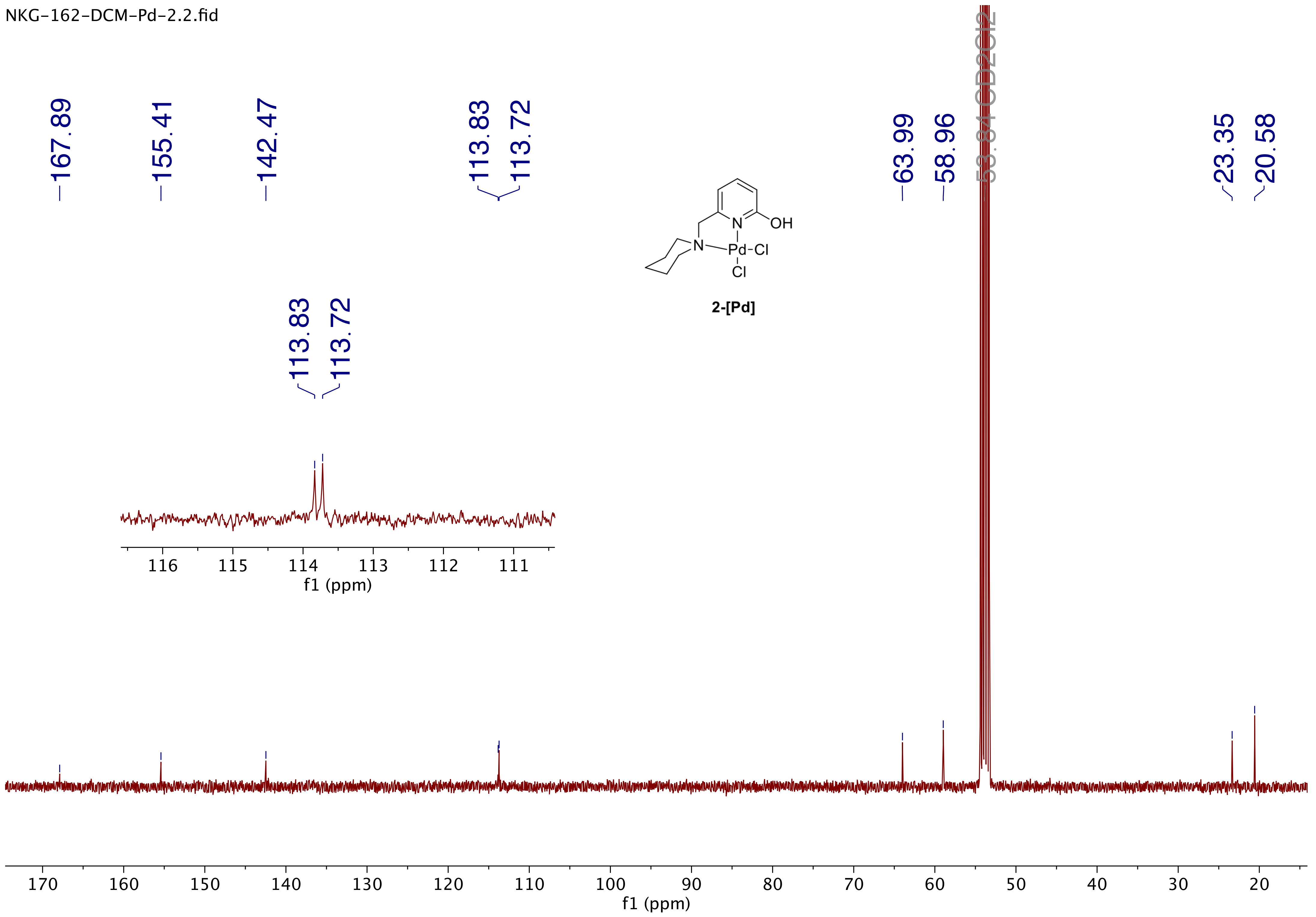
NKG-162-DCM-Pd-2.2.fid
167.89
155.41
142.47
113.83
113.72
63.99
58.96
CD2Cl2
23.35
20.58
N
OH
N
Pd-Cl
Cl
2-[Pd]
113.83
113.72
116
115
114
113
112
111
f1 (ppm)
170
160
150
140
130
120
110
100
90
80
70
60
50
40
30
20
f1 (ppm)

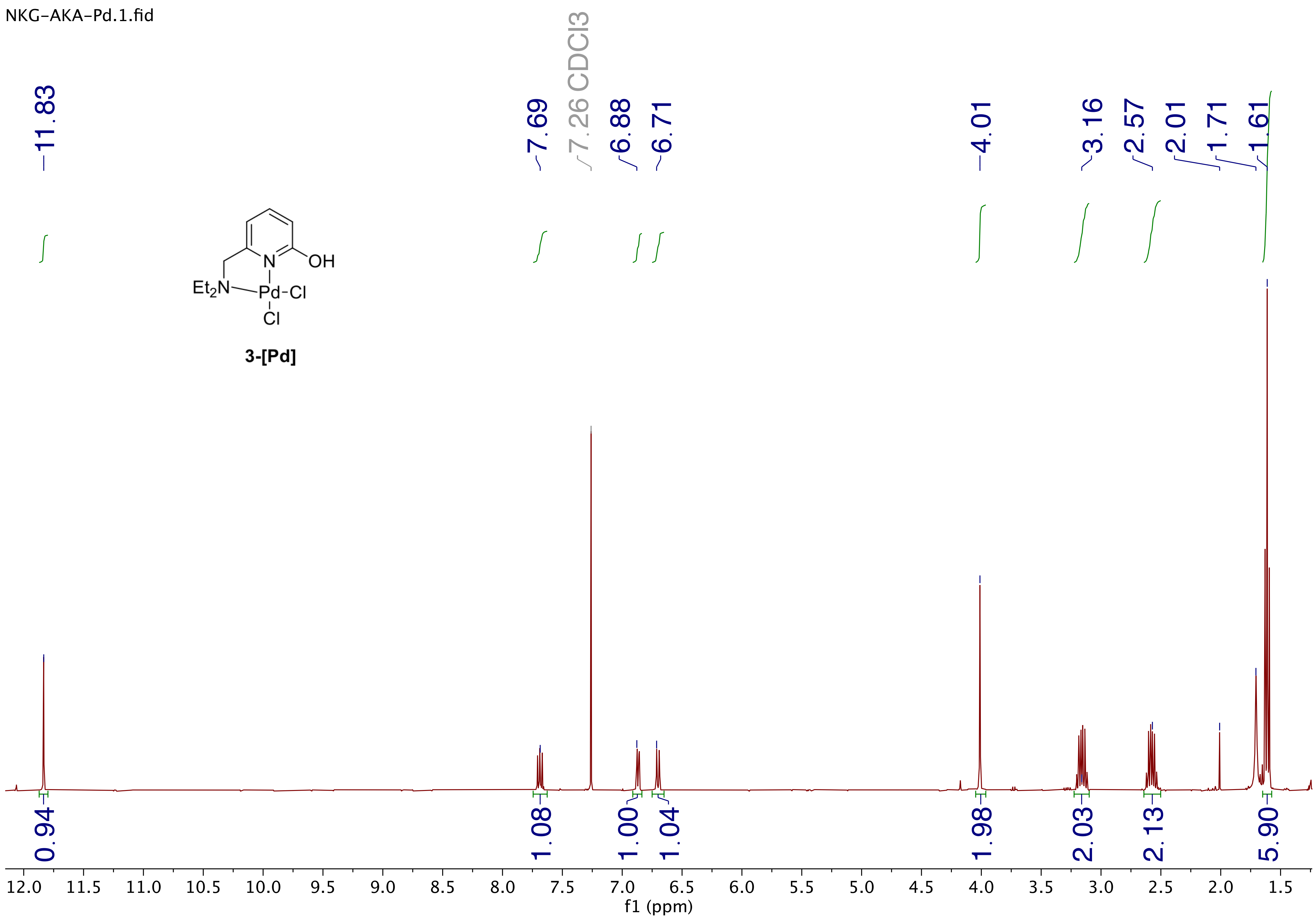
NKG-AKA-Pd.1.fid
11.83
7.69
7.26 CDCl3
6.88
6.71
4.01
3.16
2.57
2.01
1.71
1.61
OH
N
Et2N
Pd-Cl
Cl
3-[Pd]
0.94
1.08
1.00
1.04
1.98
2.03
2.13
5.90
12.0
11.5
11.0
10.5
10.0
9.5
9.0
8.5
8.0
7.5
7.0
6.5
6.0
5.5
5.0
4.5
4.0
3.5
3.0
2.5
2.0
1.5
f1 (ppm)

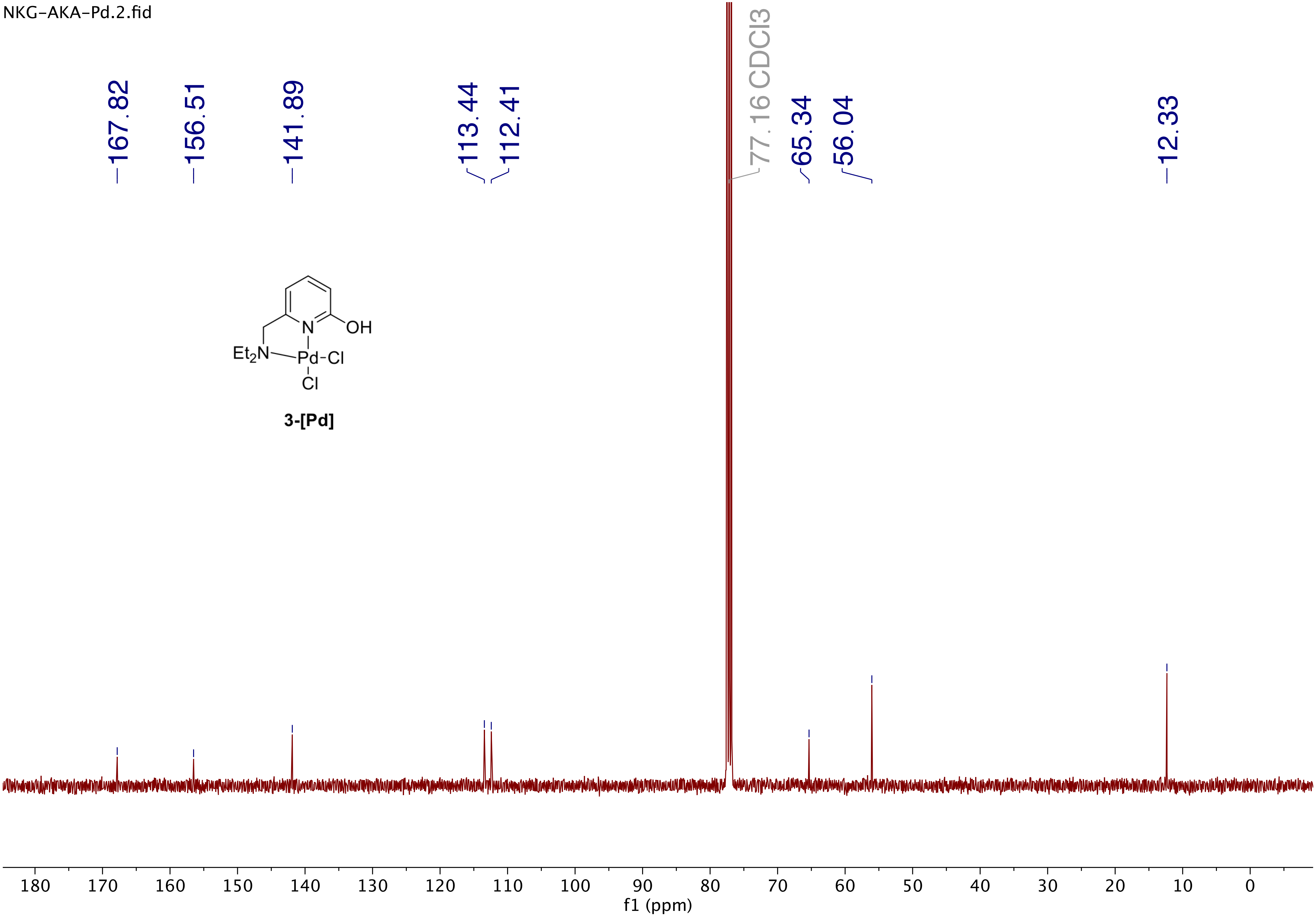
NKG-AKA-Pd.2.fid
167.82
156.51
141.89
113.44
112.41
77.16 CDCl3
65.34
56.04
12.33
OH
N
Et_2N
Pd-Cl
Cl
3-[Pd]
180
170
160
150
140
130
120
110
100
90
80
70
60
50
40
30
20
10
0
f1 (ppm)

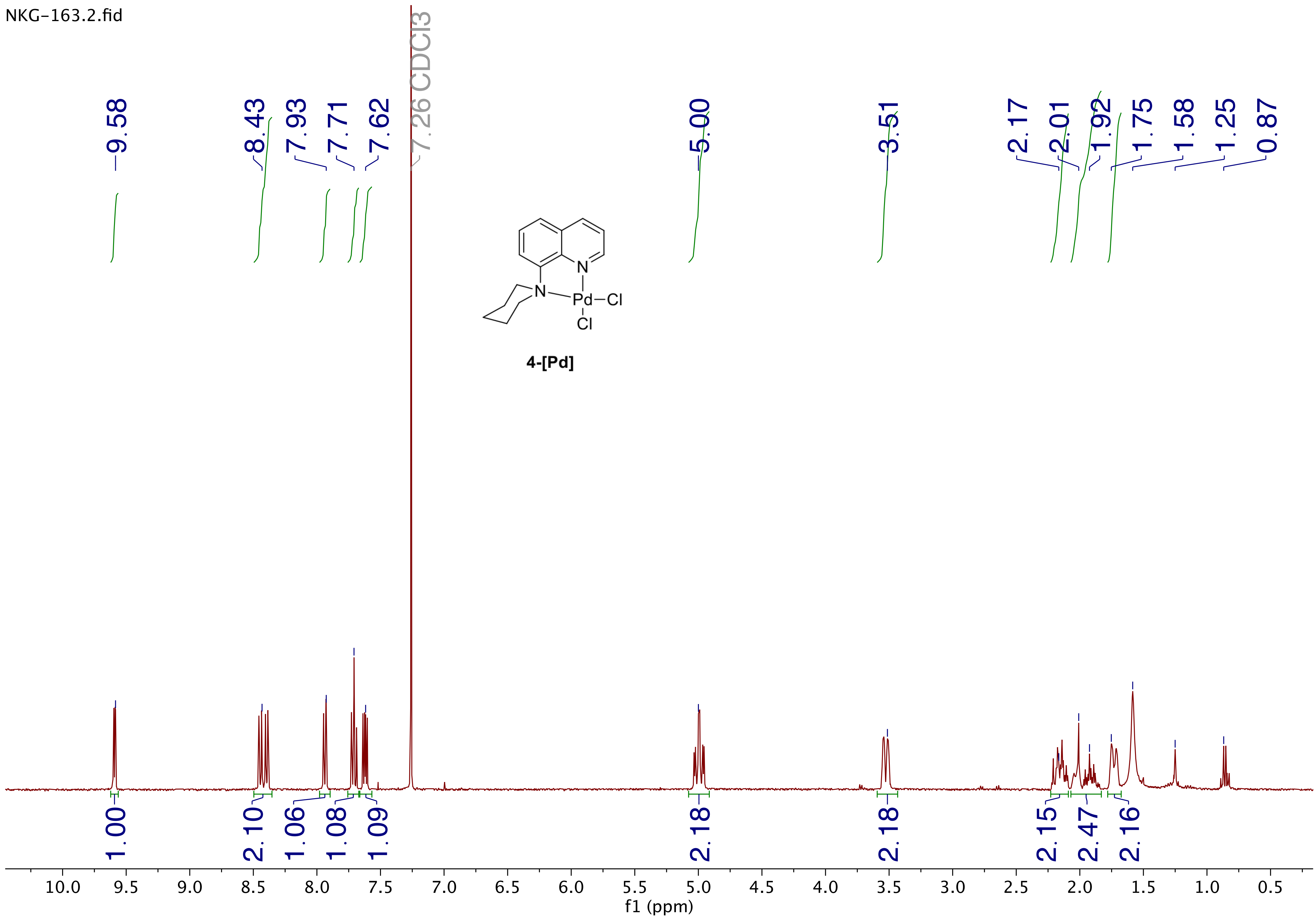

NKG-163.2.fid
9.58
8.43
7.93
7.71
7.62
7.26 CDCl3
5.00
3.51
2.17
2.01
1.92
1.75
1.58
1.25
0.87
N
N
Pd
Cl
Cl
4-[Pd]
1.00
2.10
1.06
1.08
1.09
2.18
2.18
2.15
2.47
2.16
10.0 9.5 9.0 8.5 8.0 7.5 7.0 6.5 6.0 5.5 5.0 4.5 4.0 3.5 3.0 2.5 2.0 1.5 1.0 0.5
f1 (ppm)

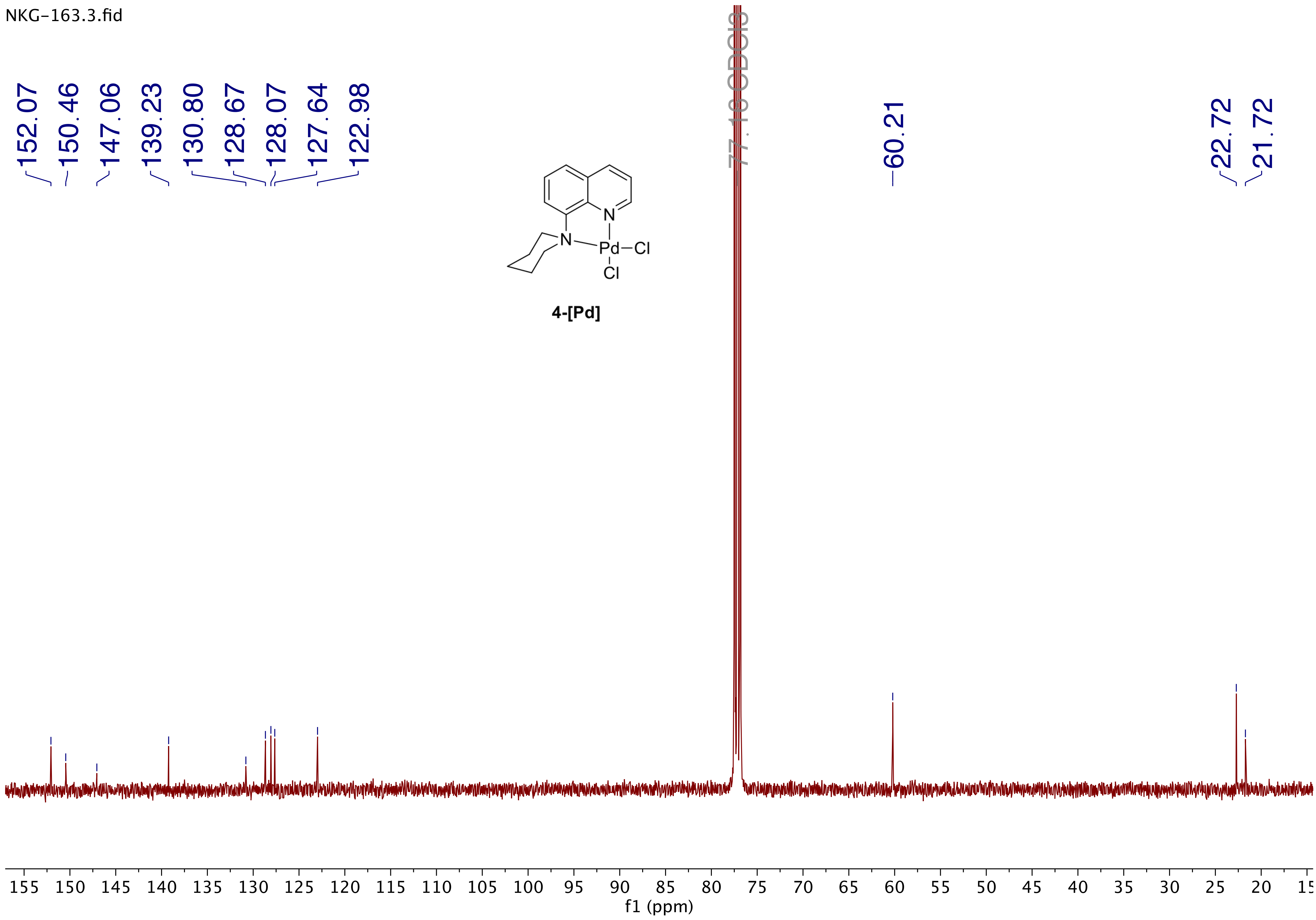
NKG-163.3.fid
152.07
150.46
147.06
139.23
130.80
128.67
128.07
127.64
122.98
77.16 CDCl3
60.21
22.72
21.72
N
N
Pd
Cl
Cl
4-[Pd]
155 150 145 140 135 130 125 120 115 110 105 100 95 90 85 80 75 70 65 60 55 50 45 40 35 30 25 20 15
f1 (ppm)

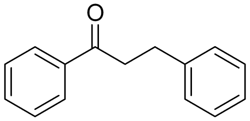

3a

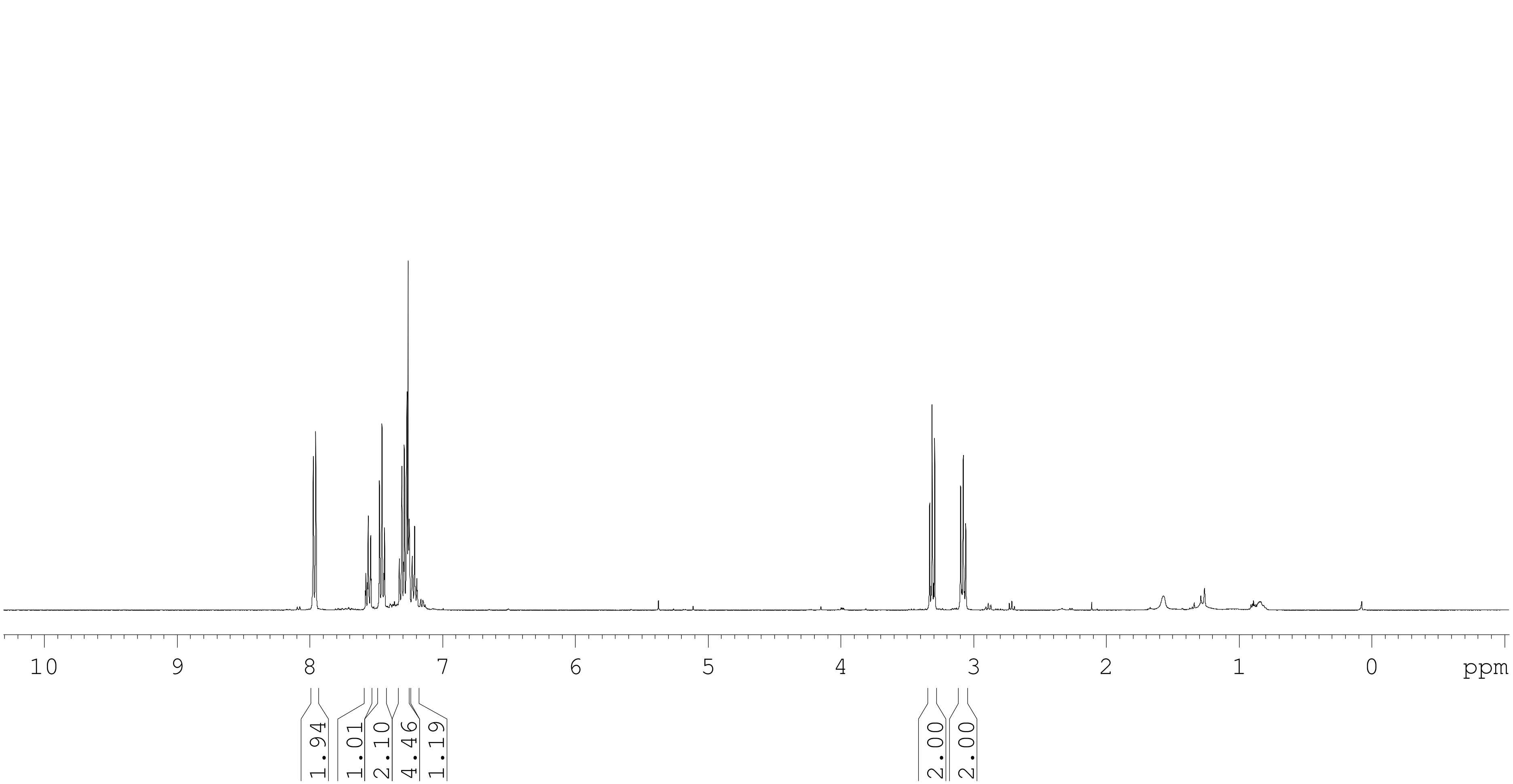

7.975
7.957
7.582
7.579
7.576
7.566
7.561
7.556
7.546
7.542
7.539
7.476
7.457
7.439
7.326
7.307
7.295
7.289
7.273
7.269
7.260
7.252
7.233
7.229
7.225
7.211
7.194
3.331
3.314
3.293
3.098
3.078
3.059
10
9
8
7
6
5
4
3
2
1
0
ppm
1.94
1.01
2.10
4.46
1.19
2.00
2.00

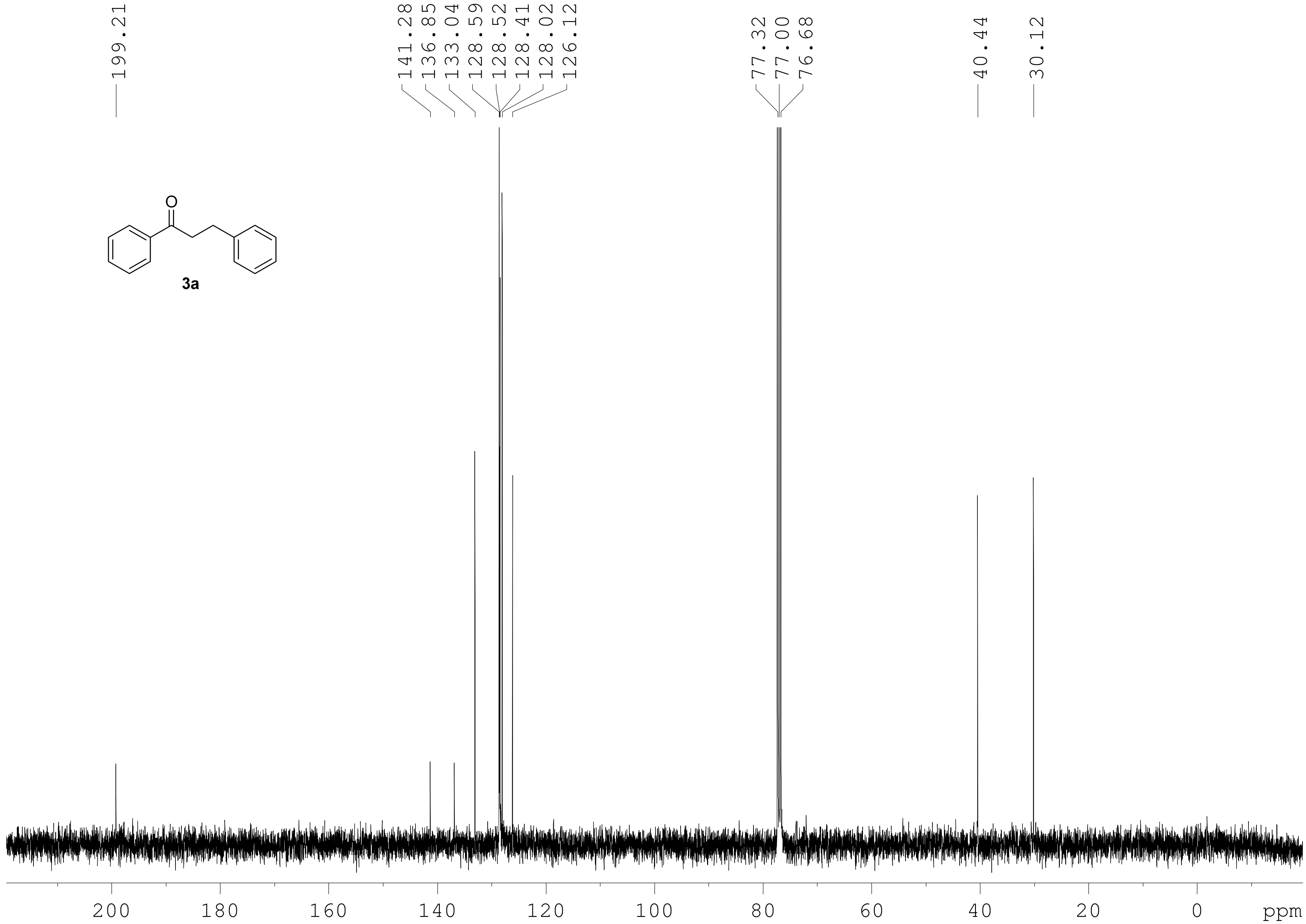
199.21
141.28
136.85
133.04
128.59
128.52
128.41
128.02
126.12
77.32
77.00
76.68
40.44
30.12
O
3a
200
180
160
140
120
100
80
60
40
20
0
ppm

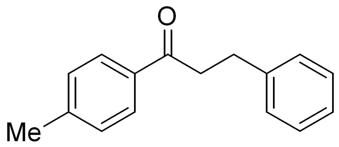

Me
O

3b

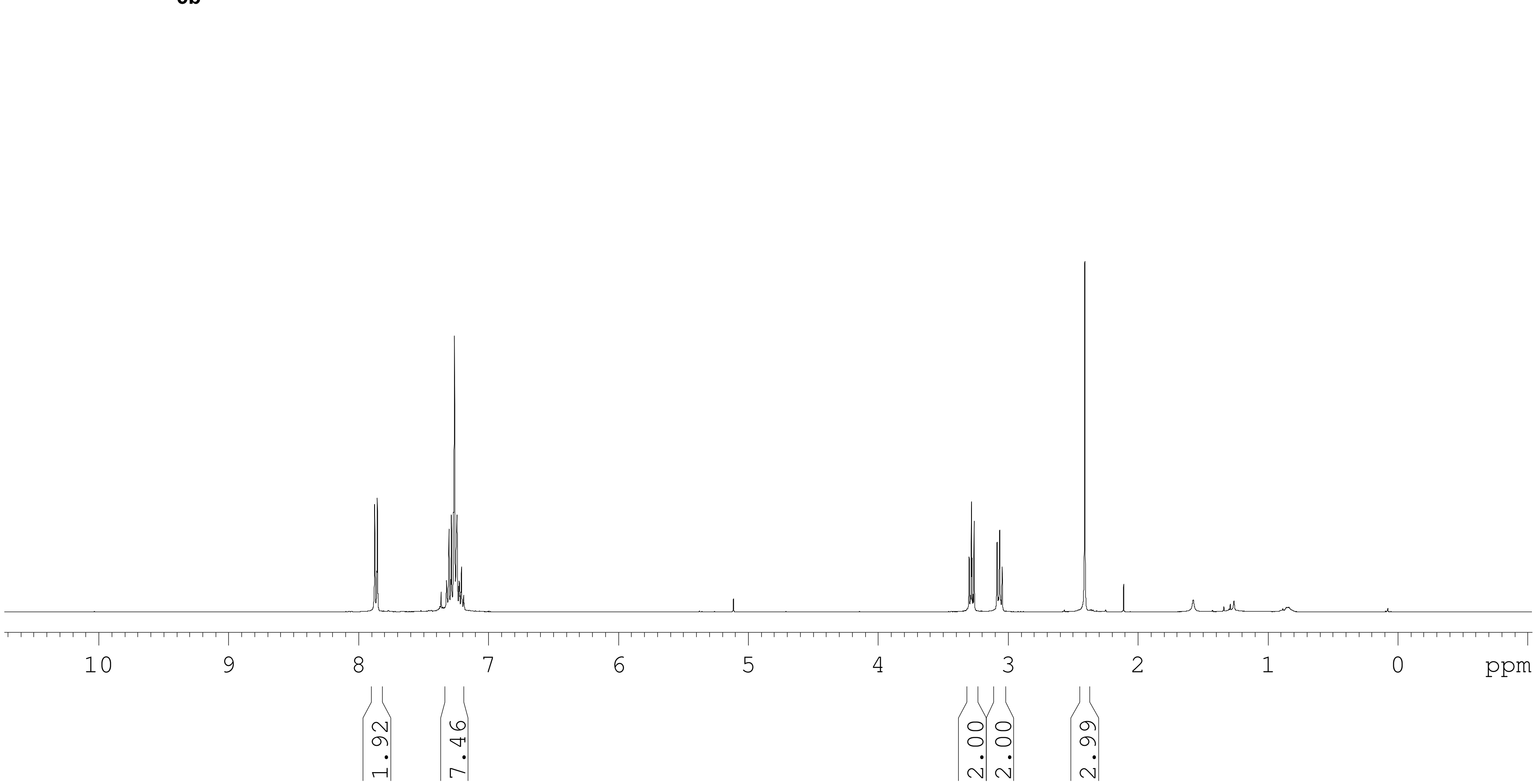

7.874
7.853
7.320
7.302
7.289
7.284
7.267
7.262
7.259
7.245
7.240
7.227
7.223
7.205
3.297
3.280
3.259
3.083
3.062
3.044
2.408
10
9
8
7
6
5
4
3
2
1
0
ppm
1.92
7.46
2.00
2.00
2.99

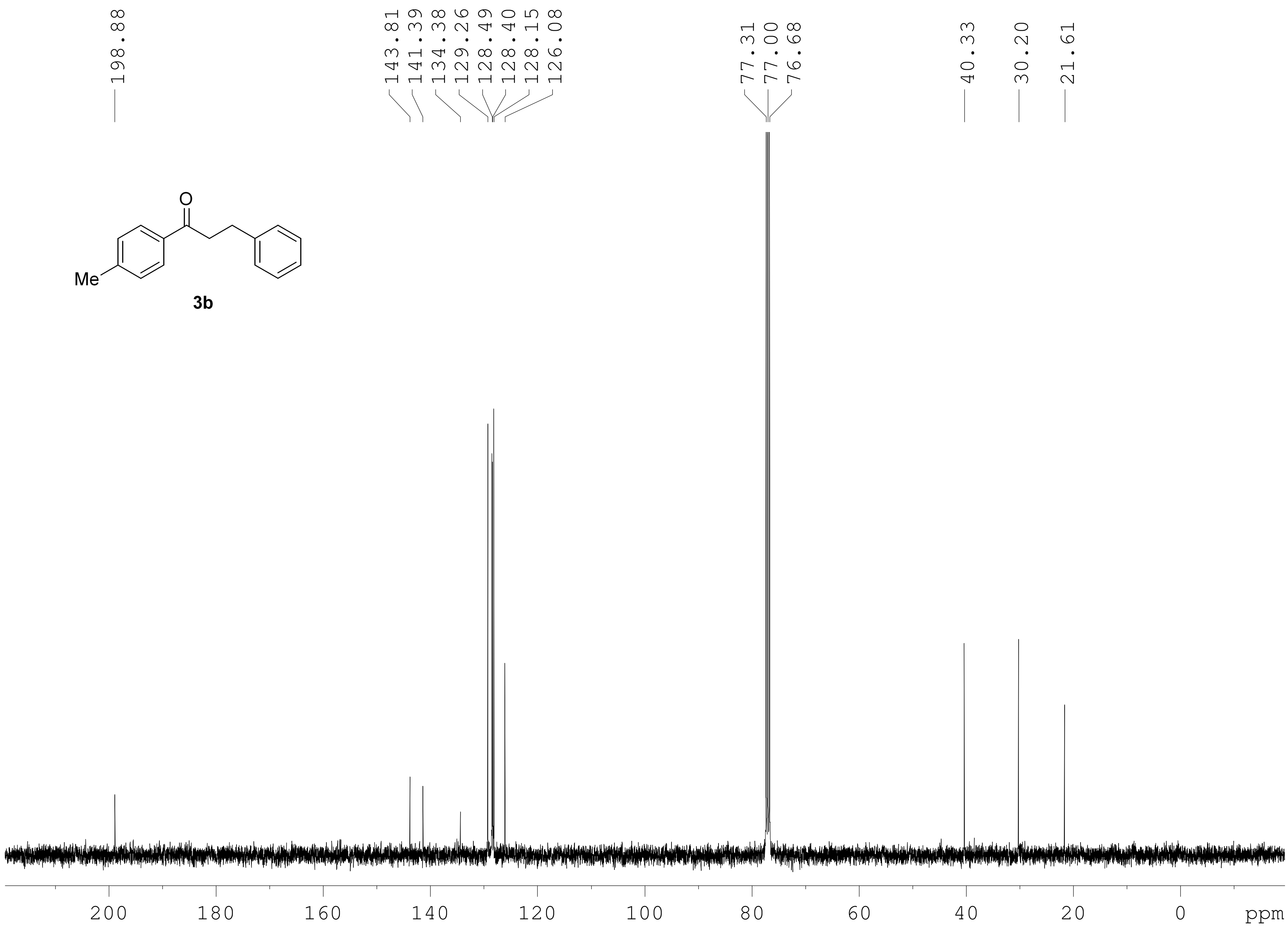
198.88
143.81
141.39
134.38
129.26
128.49
128.40
128.15
126.08
77.31
77.00
76.68
40.33
30.20
21.61
O
Me
3b
200
180
160
140
120
100
80
60
40
20
0
ppm

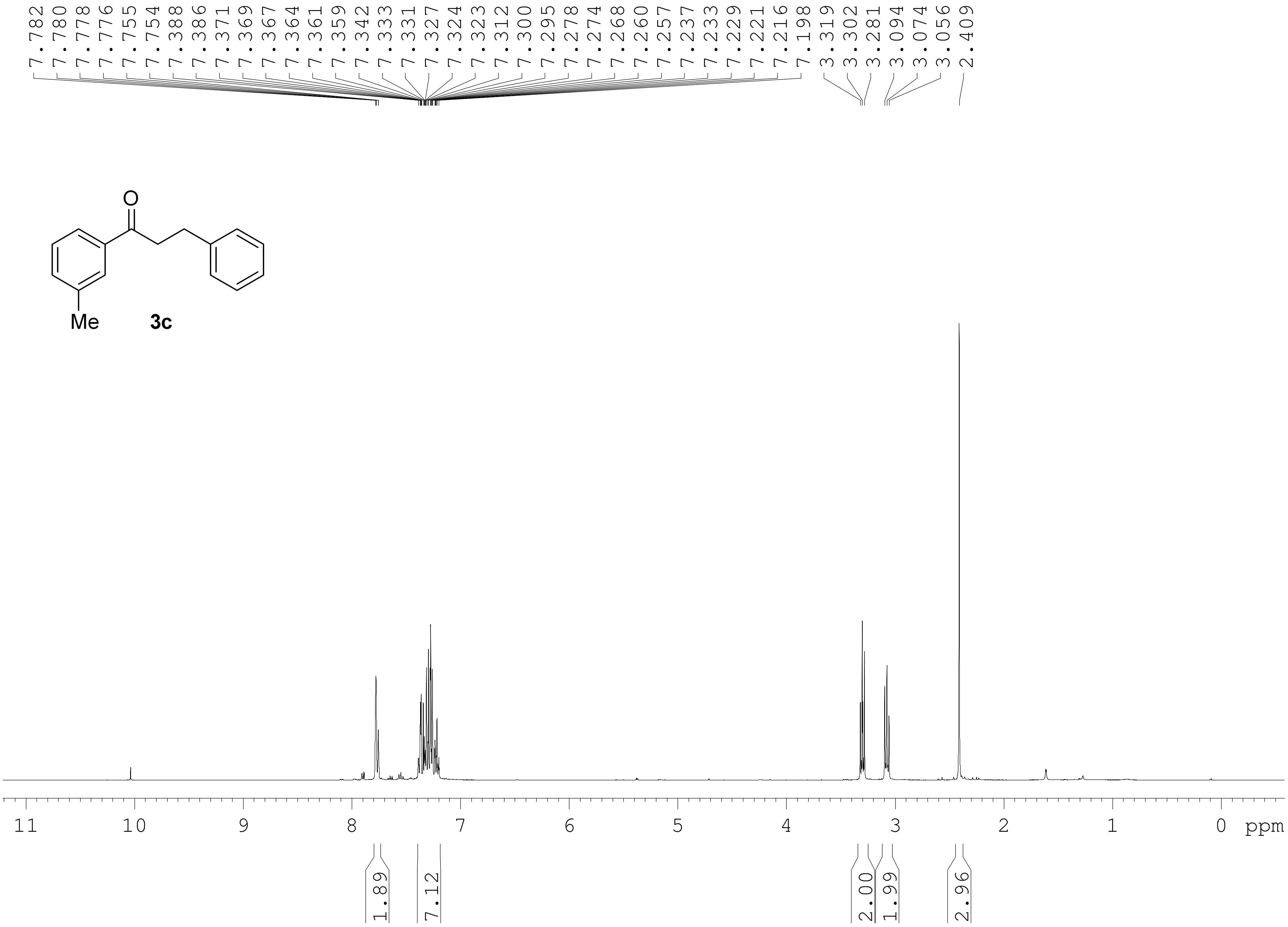

O
Me
3c
7.782
7.780
7.778
7.776
7.755
7.754
7.388
7.386
7.371
7.369
7.367
7.364
7.361
7.359
7.342
7.333
7.331
7.327
7.324
7.323
7.312
7.300
7.295
7.278
7.274
7.268
7.260
7.257
7.237
7.233
7.229
7.221
7.216
7.198
3.319
3.302
3.281
3.094
3.074
3.056
2.409
11
10
9
8
7
6
5
4
3
2
1
0 ppm
1.89
7.12
2.00
1.99
2.96

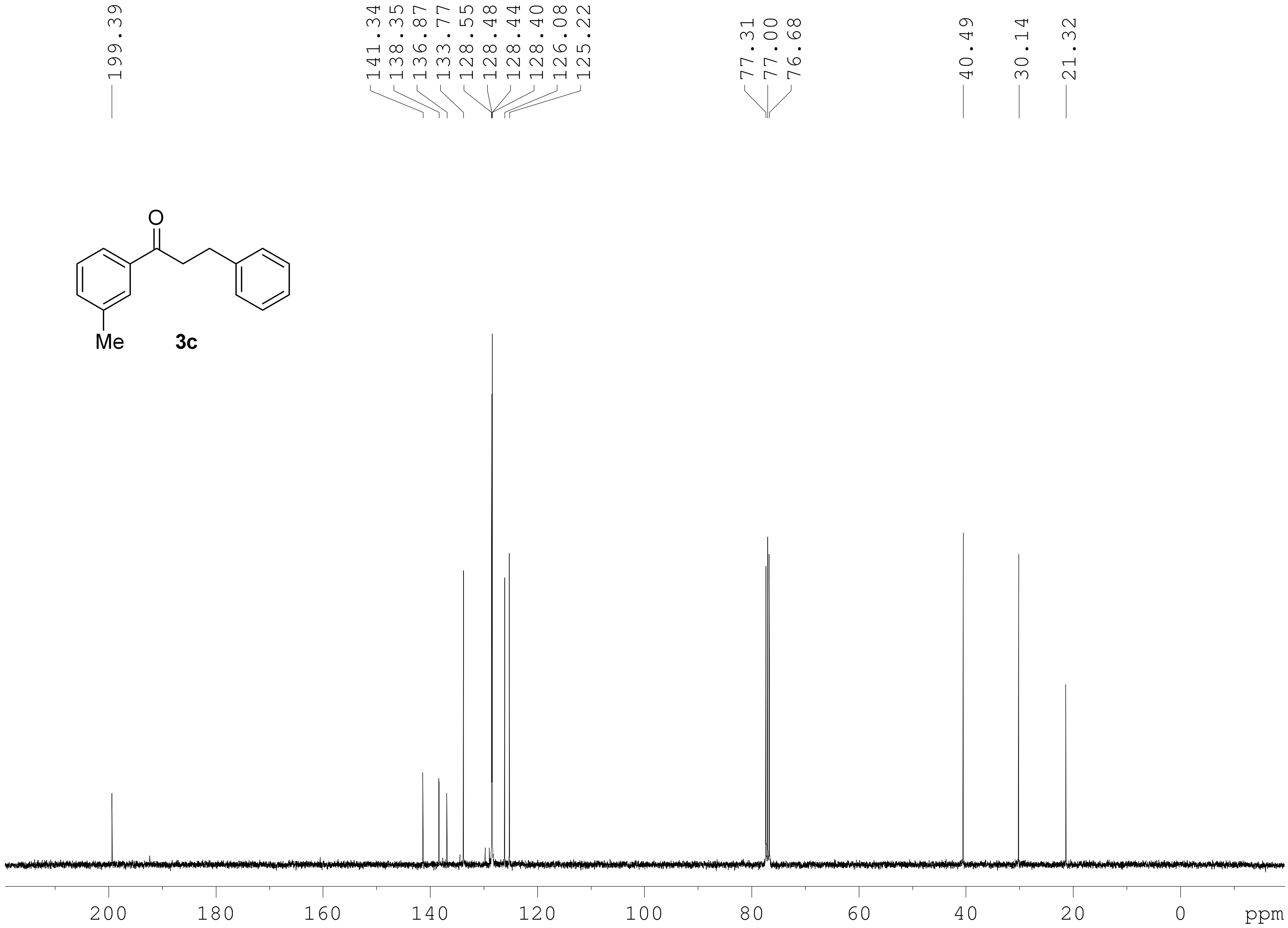

199.39
141.34
138.35
136.87
133.77
128.55
128.48
128.44
128.40
126.08
125.22
77.31
77.00
76.68
40.49
30.14
21.32
O
Me
3c
200
180
160
140
120
100
80
60
40
20
0
ppm

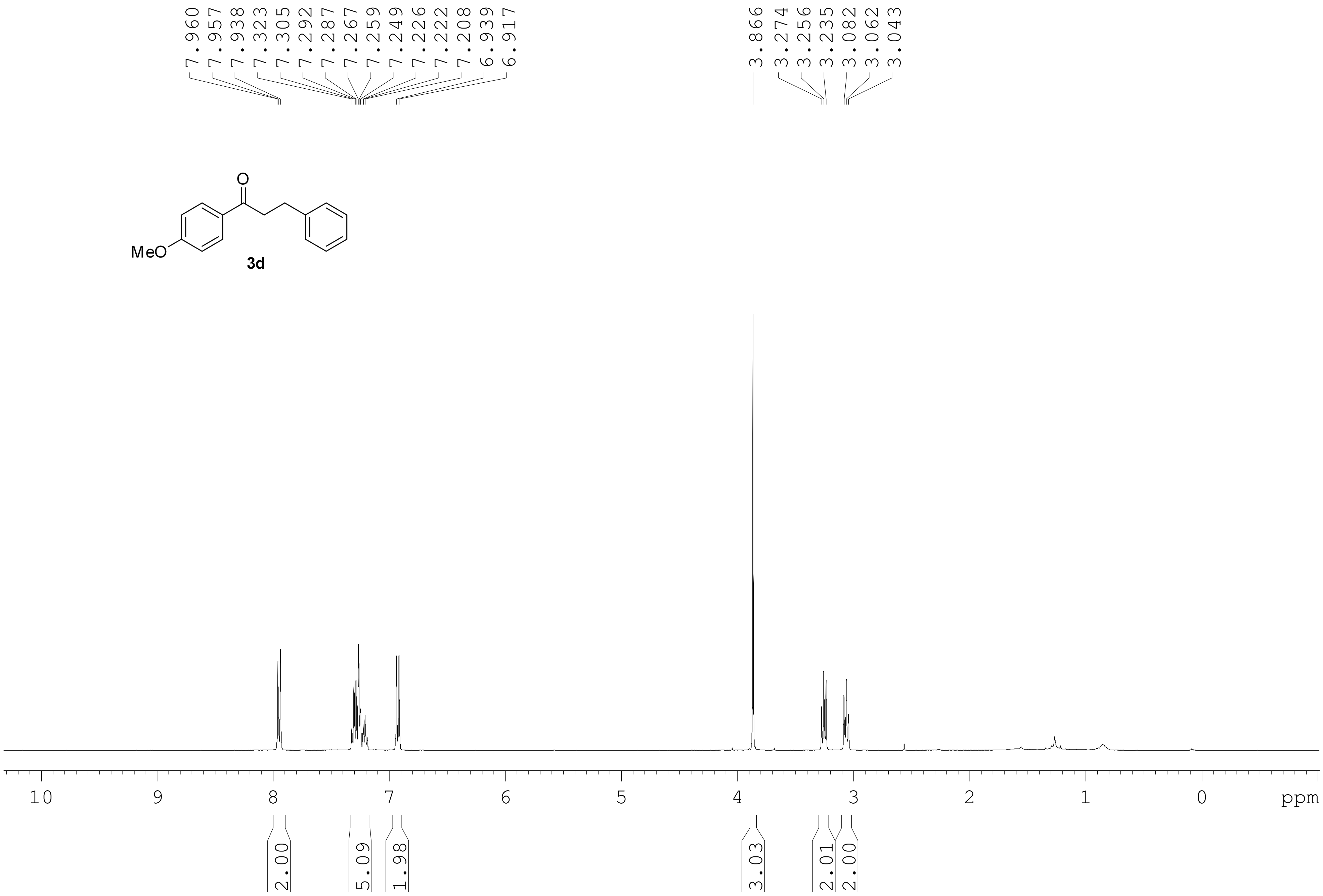
7.960
7.957
7.938
7.323
7.305
7.292
7.287
7.267
7.259
7.249
7.226
7.222
7.208
6.939
6.917
3.866
3.274
3.256
3.235
3.082
3.062
3.043
O
MeO
3d
10
9
8
7
6
5
4
3
2
1
0
ppm
2.00
5.09
1.98
3.03
2.01
2.00

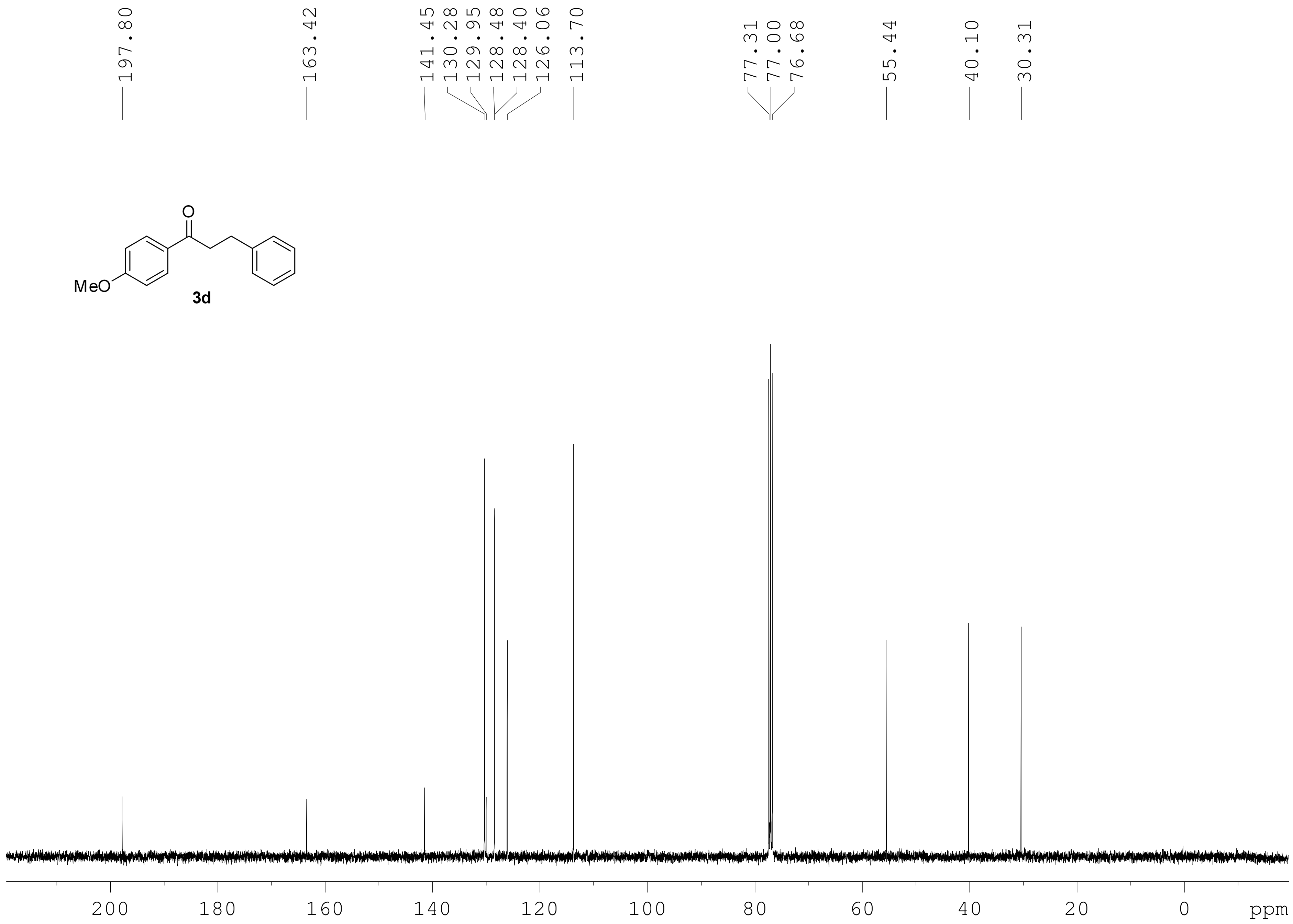
197.80
163.42
141.45
130.28
129.95
128.48
128.40
126.06
113.70
77.31
77.00
76.68
55.44
40.10
30.31
O
MeO
3d
200
180
160
140
120
100
80
60
40
20
0
ppm

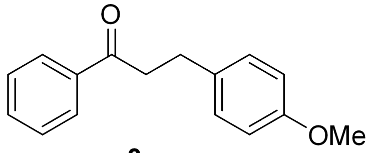
O
OMe

3e

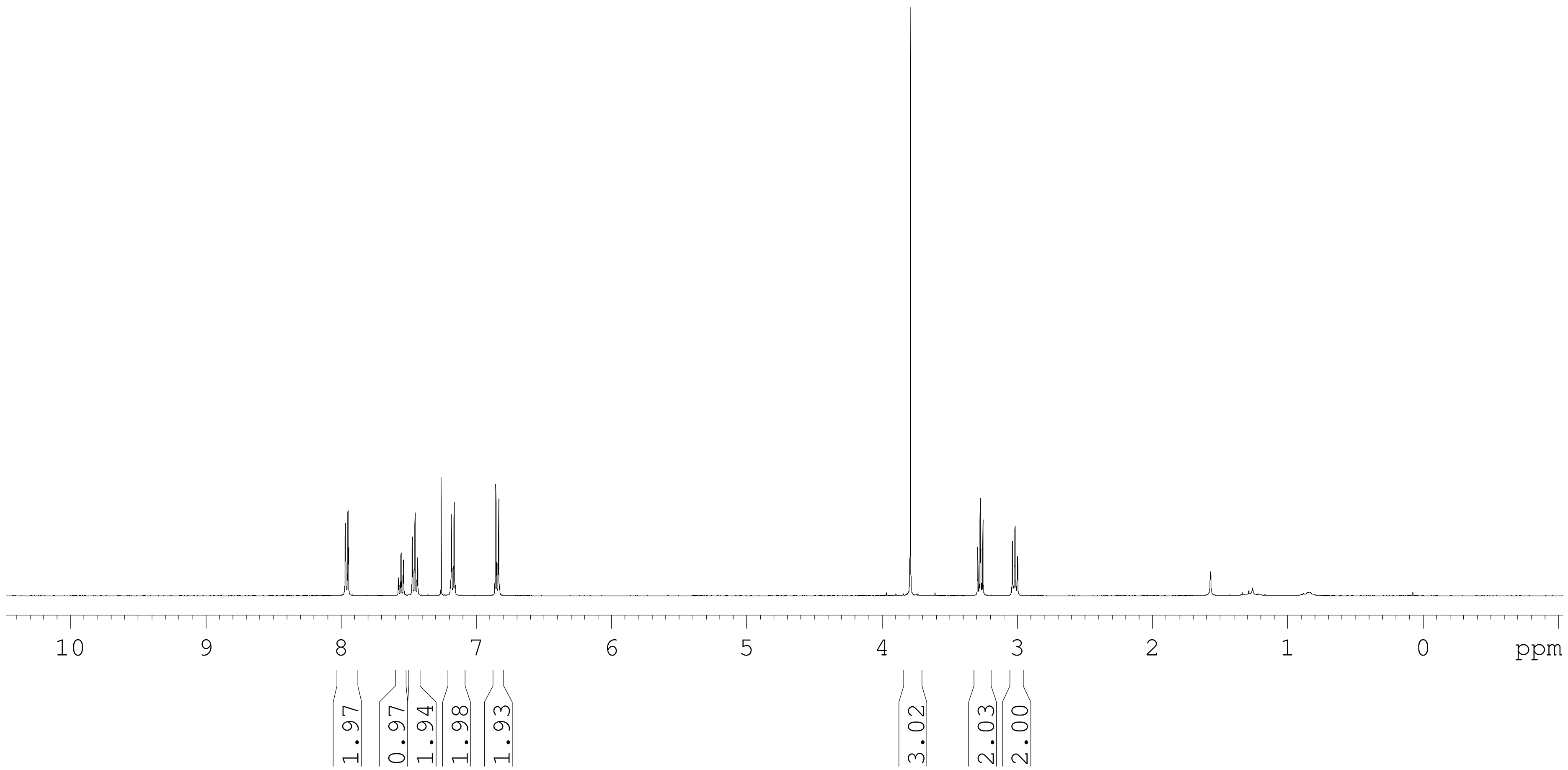
7.966
7.949
7.577
7.574
7.571
7.561
7.556
7.551
7.541
7.537
7.534
7.472
7.452
7.434
7.185
7.163
6.855
6.833
3.790
3.291
3.274
3.253
3.036
3.016
2.998
10
9
8
7
6
5
4
3
2
1
0
ppm
1.97
0.97
1.94
1.98
1.93
3.02
2.03
2.00

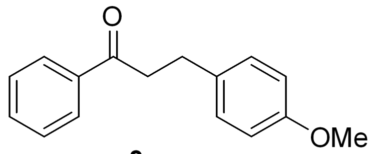
O
OMe

3e

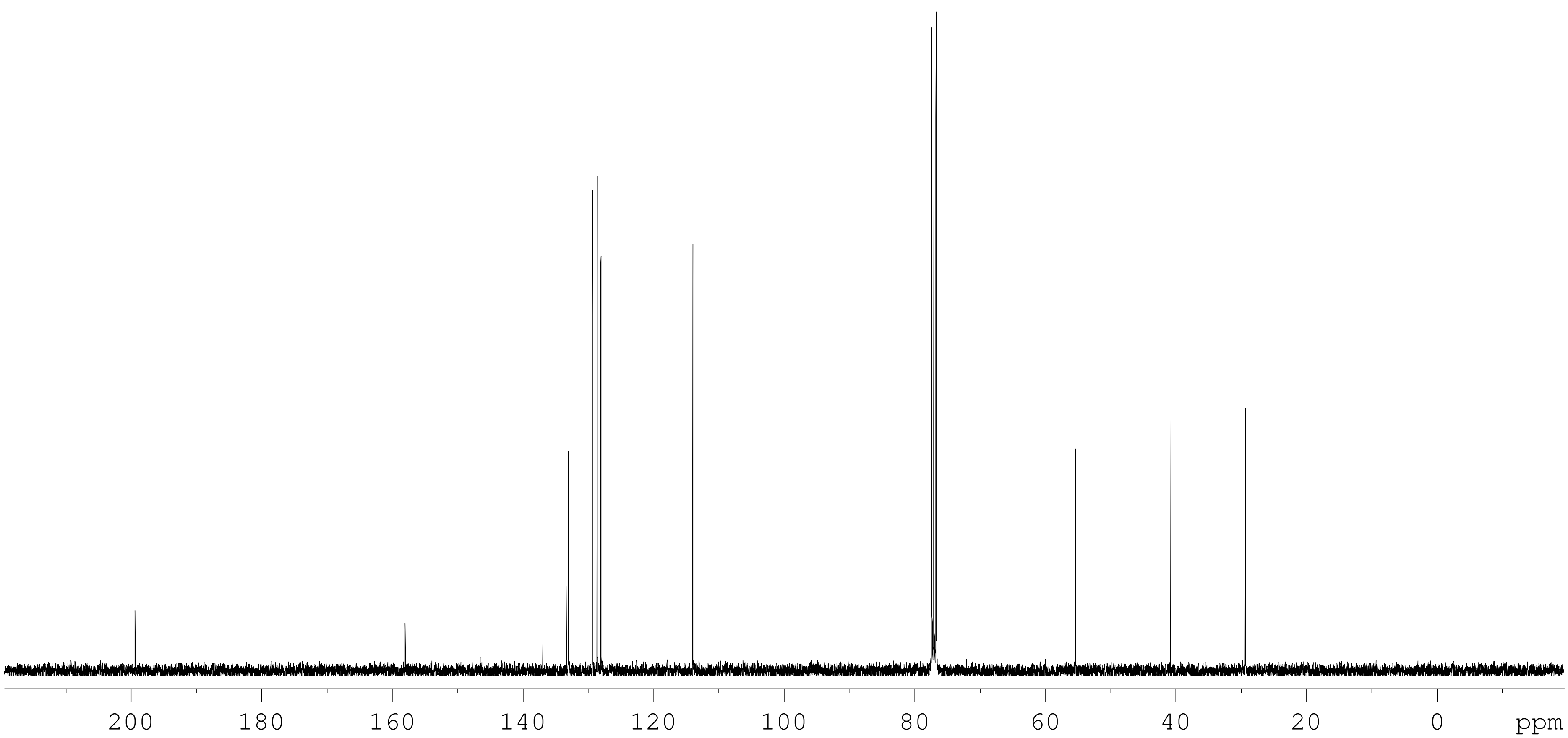
199.37
157.97
136.89
133.30
133.01
129.33
128.57
128.02
113.92
77.31
77.00
76.68
55.26
40.70
29.26
200
180
160
140
120
100
80
60
40
20
0
ppm

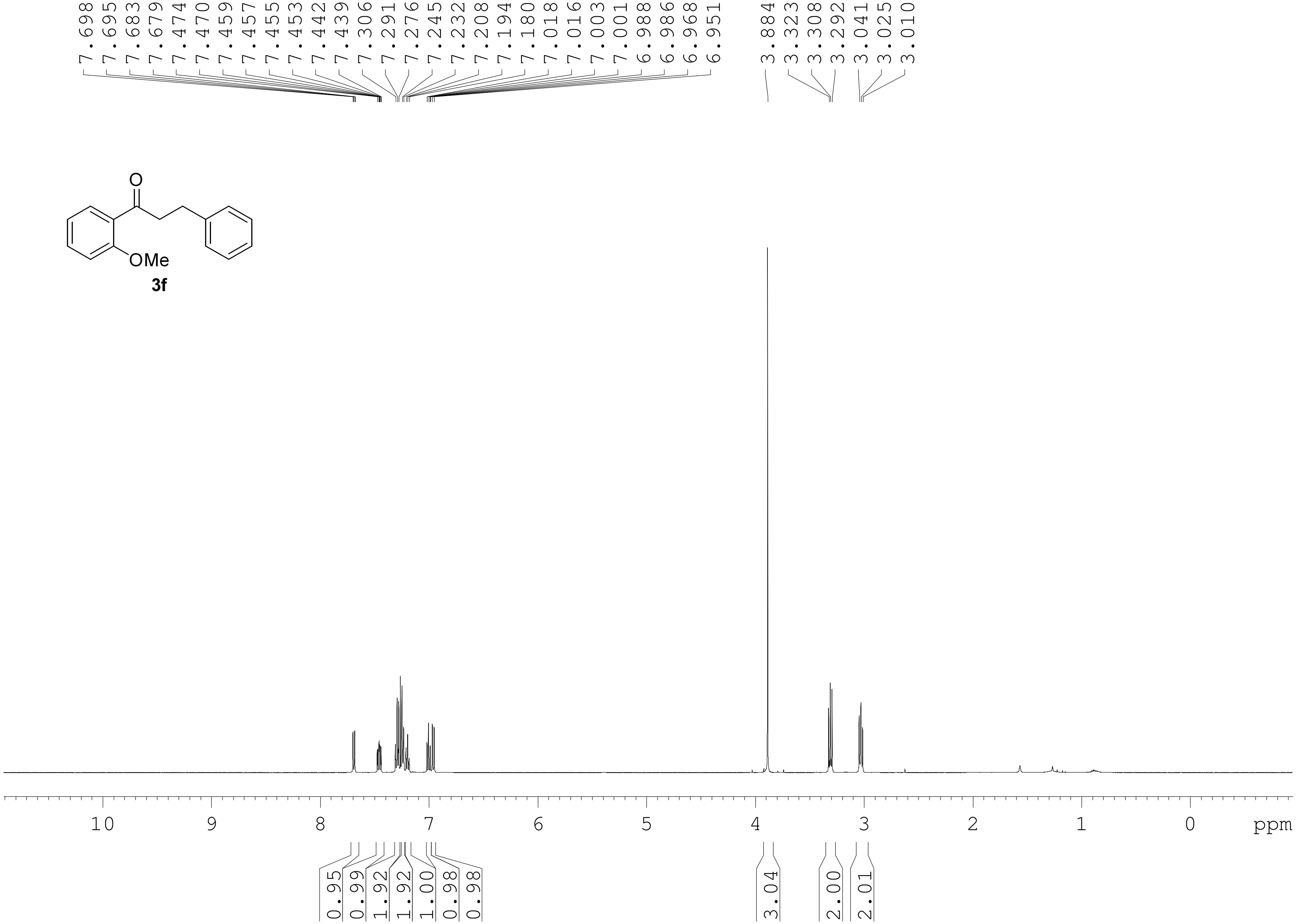
7.698
7.695
7.683
7.679
7.474
7.470
7.459
7.457
7.455
7.453
7.442
7.439
7.306
7.291
7.276
7.245
7.232
7.208
7.194
7.180
7.018
7.016
7.003
7.001
6.988
6.986
6.968
6.951
3.884
3.323
3.308
3.292
3.041
3.025
3.010
O
OMe
10
9
8
7
6
5
4
3
2
1
0
ppm
0.95
0.99
1.92
1.92
1.00
0.98
0.98
3.04
2.00
2.01

3f

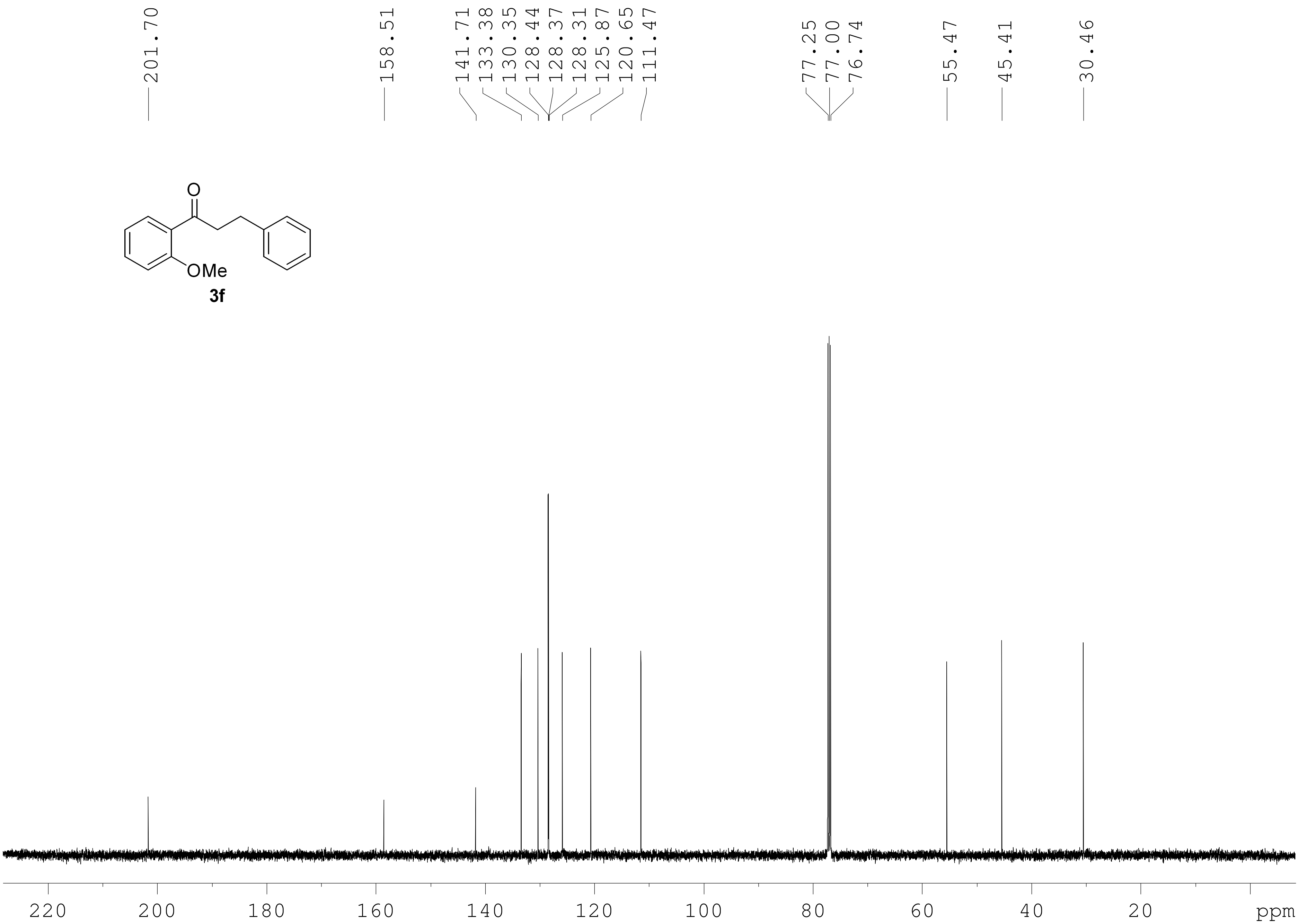

201.70
158.51
141.71
133.38
130.35
128.44
128.37
128.31
125.87
120.65
111.47
77.25
77.00
76.74
55.47
45.41
30.46
O
OMe
3f
220
200
180
160
140
120
100
80
60
40
20
ppm

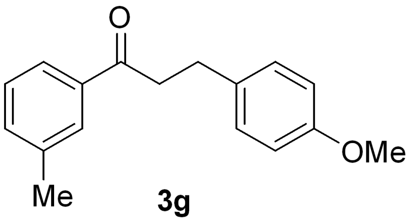

O
OMe
Me

3g

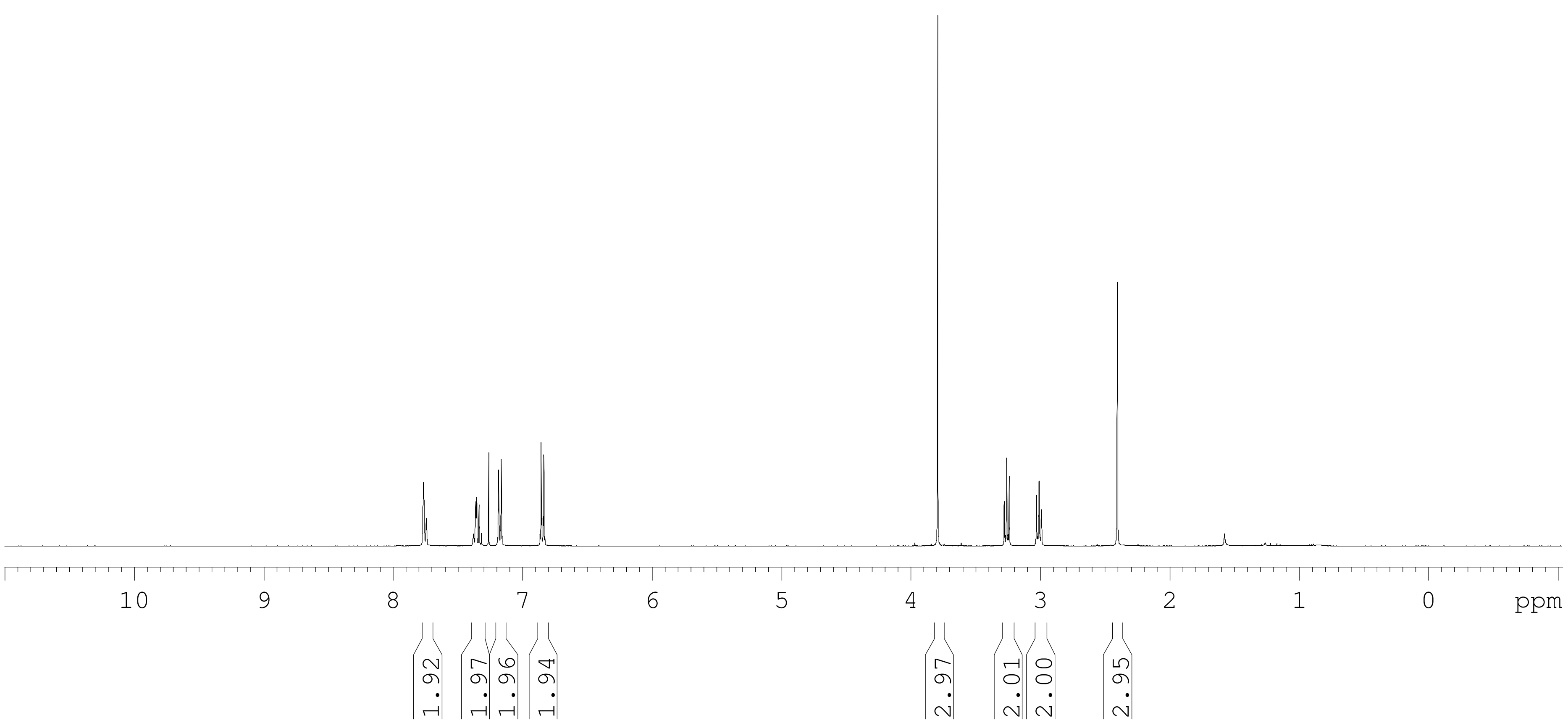

7.763
7.740
7.378
7.377
7.374
7.359
7.358
7.355
7.352
7.350
7.333
7.314
7.184
7.162
6.855
6.833
3.790
3.275
3.257
3.237
3.026
3.006
2.988
2.401
10
9
8
7
6
5
4
3
2
1
0
ppm
1.92
1.97
1.96
1.94
2.97
2.01
2.00
2.95

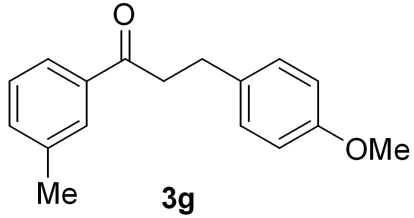

3g

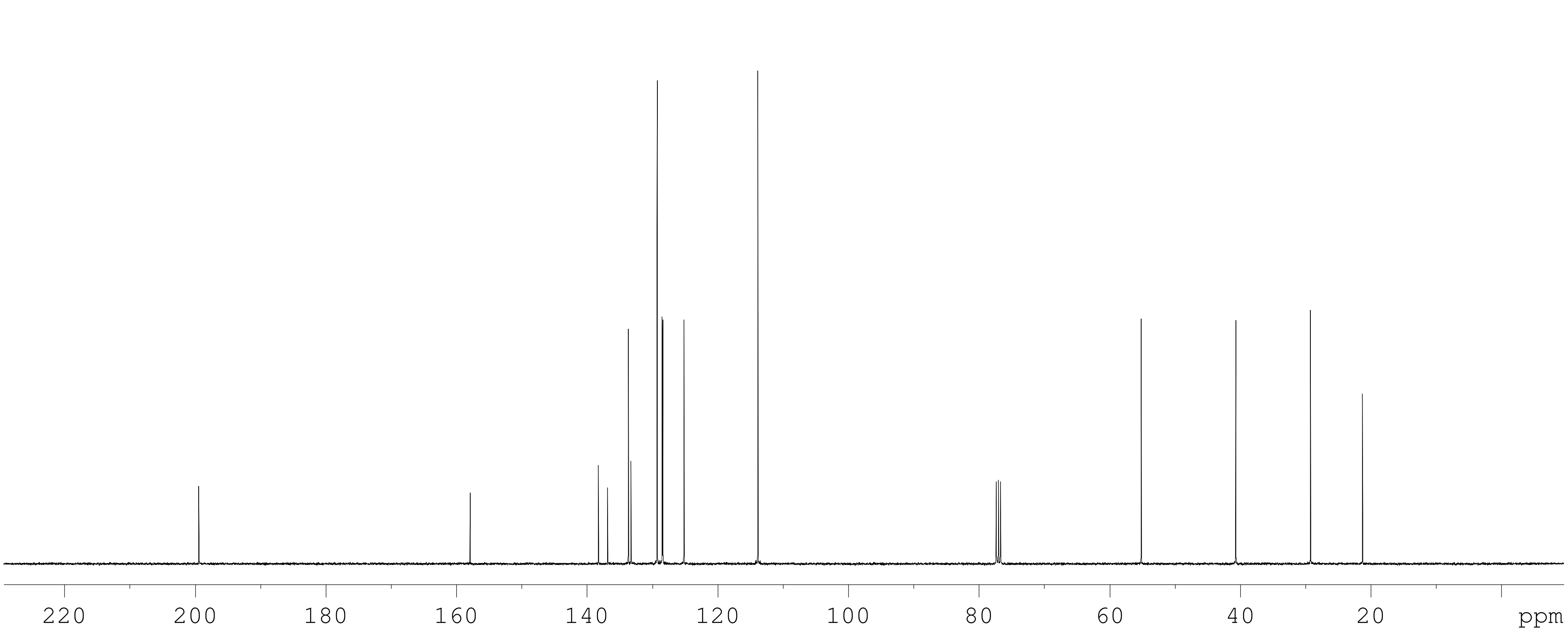
199.43
157.88
138.24
136.84
133.66
133.27
129.25
128.47
128.35
125.14
113.82
77.31
77.00
76.68
55.13
40.64
29.20
21.23
220
200
180
160
140
120
100
80
60
40
20
ppm

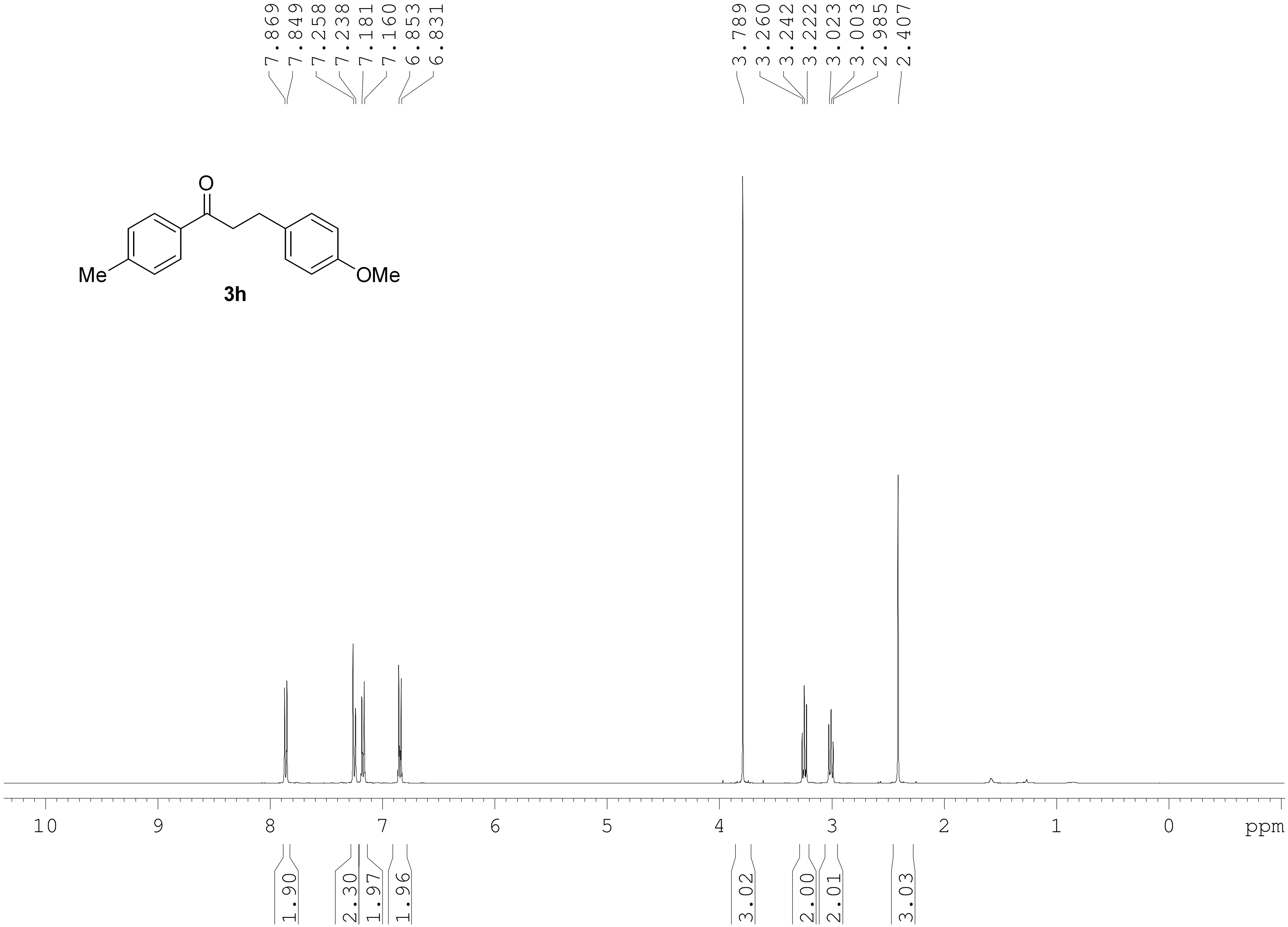
7.869
7.849
7.258
7.238
7.181
7.160
6.853
6.831
3.789
3.260
3.242
3.222
3.023
3.003
2.985
2.407
O
Me
OMe
3h
10
9
8
7
6
5
4
3
2
1
0
ppm
1.90
2.30
1.97
1.96
3.02
2.00
2.01
3.03

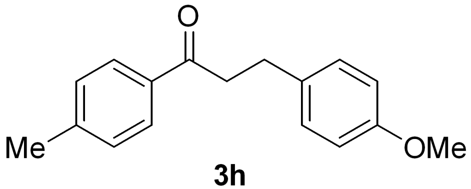
O
Me
OMe

3h

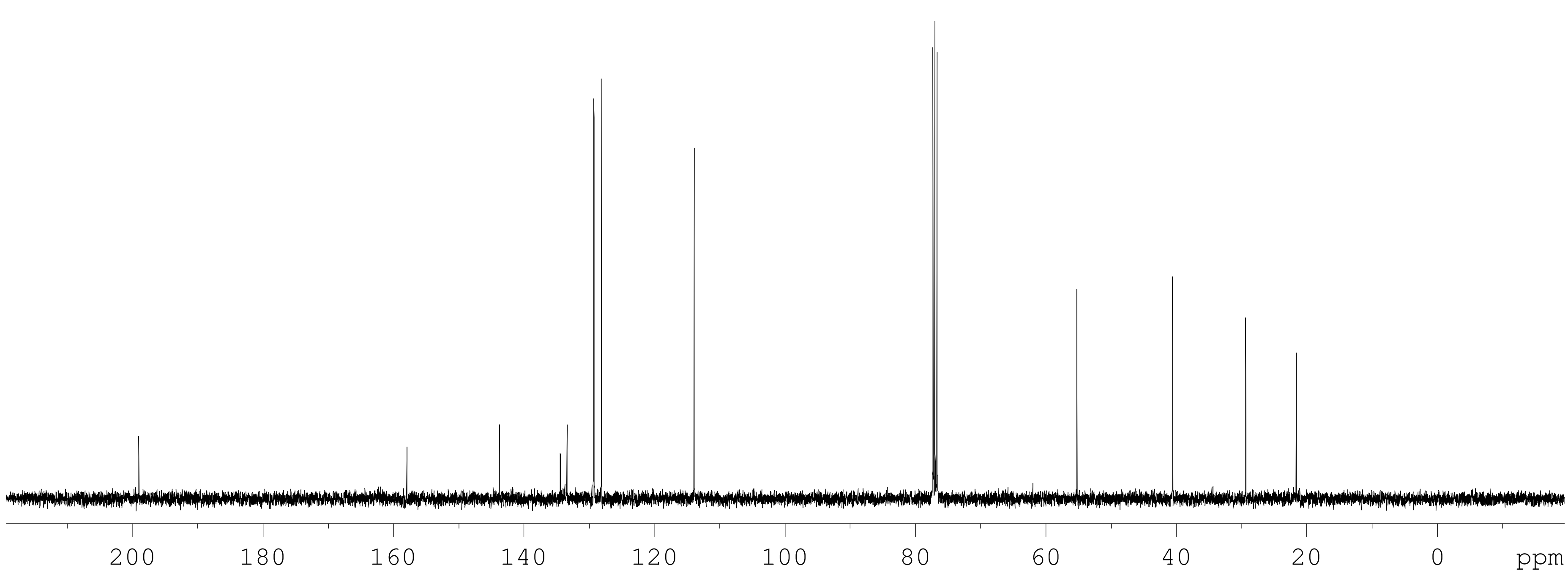
199.04
157.94
143.76
134.42
133.41
129.32
129.24
128.14
113.90
77.31
77.00
76.68
55.25
40.58
29.35
21.60
200
180
160
140
120
100
80
60
40
20
0
ppm

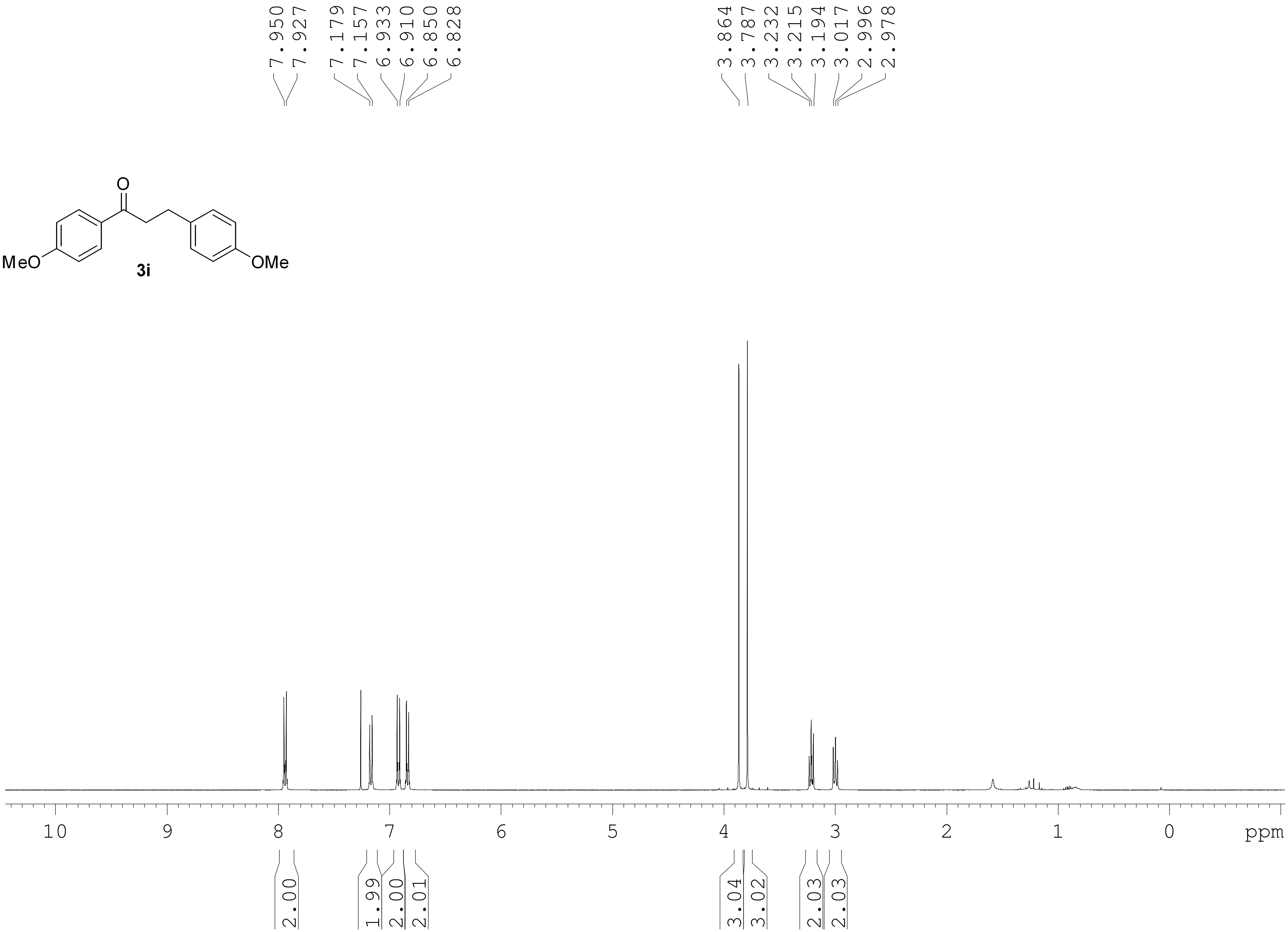
7.950
7.927
7.179
7.157
6.933
6.910
6.850
6.828
3.864
3.787
3.232
3.215
3.194
3.017
2.996
2.978
O
MeO
3i
OMe
10
9
8
7
6
5
4
3
2
1
0
ppm
2.00
1.99
2.00
2.01
3.04
3.02
2.03
2.03

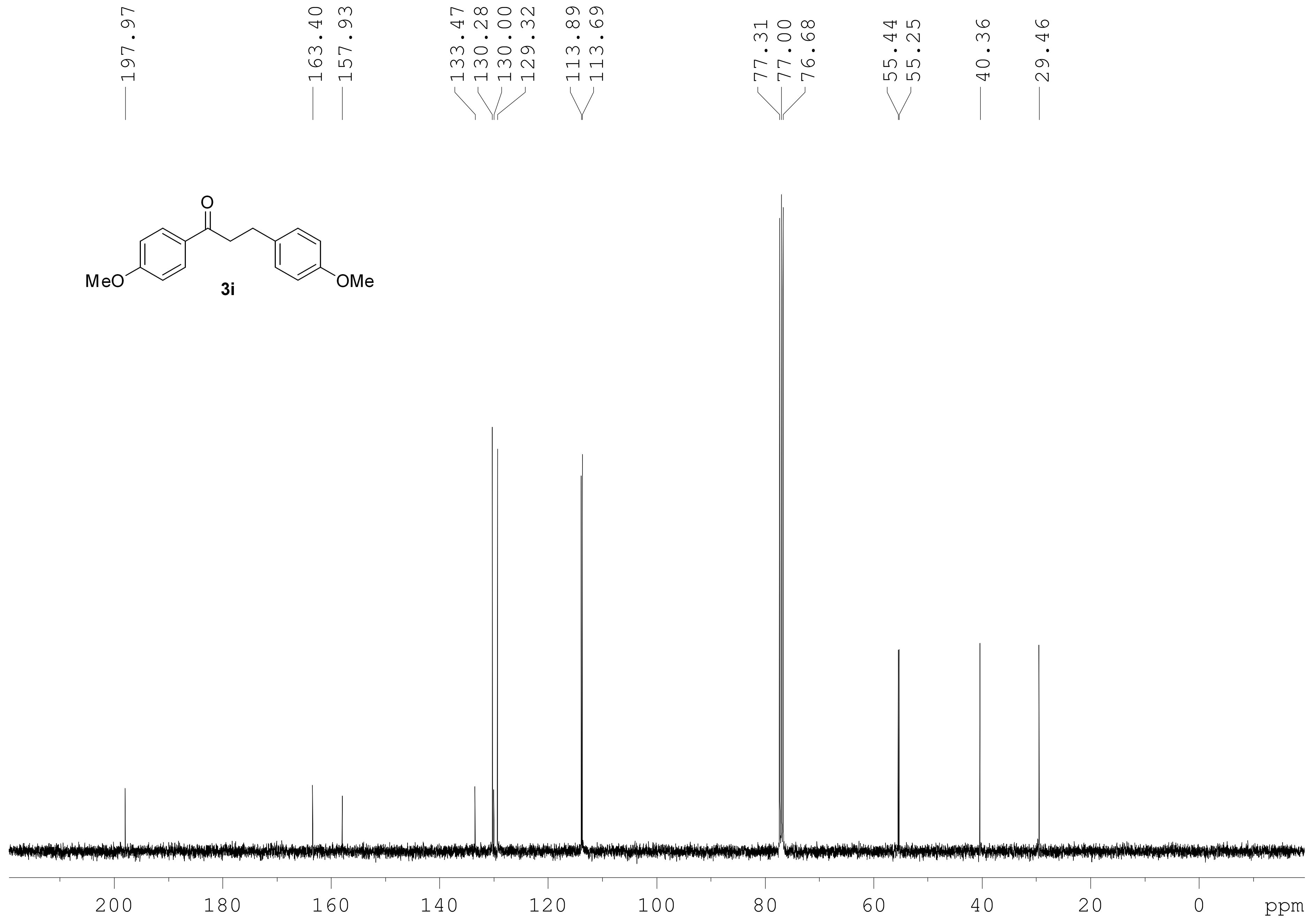

197.97
163.40
157.93
133.47
130.28
130.00
129.32
113.89
113.69
77.31
77.00
76.68
55.44
55.25
40.36
29.46
O
MeO
OMe
3i
200
180
160
140
120
100
80
60
40
20
0
ppm

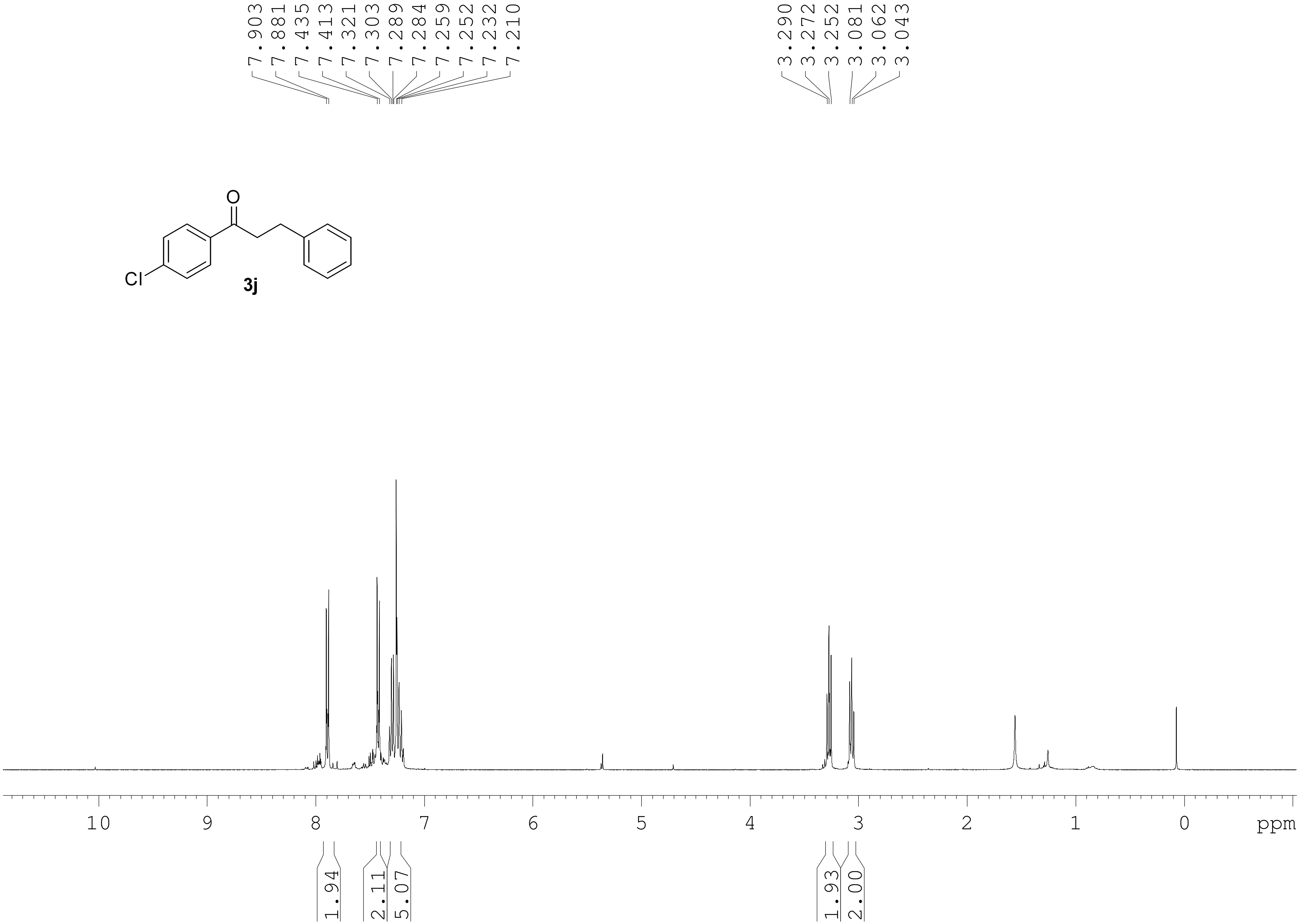

7.903
7.881
7.435
7.413
7.321
7.303
7.289
7.284
7.259
7.252
7.232
7.210
3.290
3.272
3.252
3.081
3.062
3.043
O
Cl
10
9
8
7
6
5
4
3
2
1
0
ppm
1.94
2.11
5.07
1.93
2.00

3j

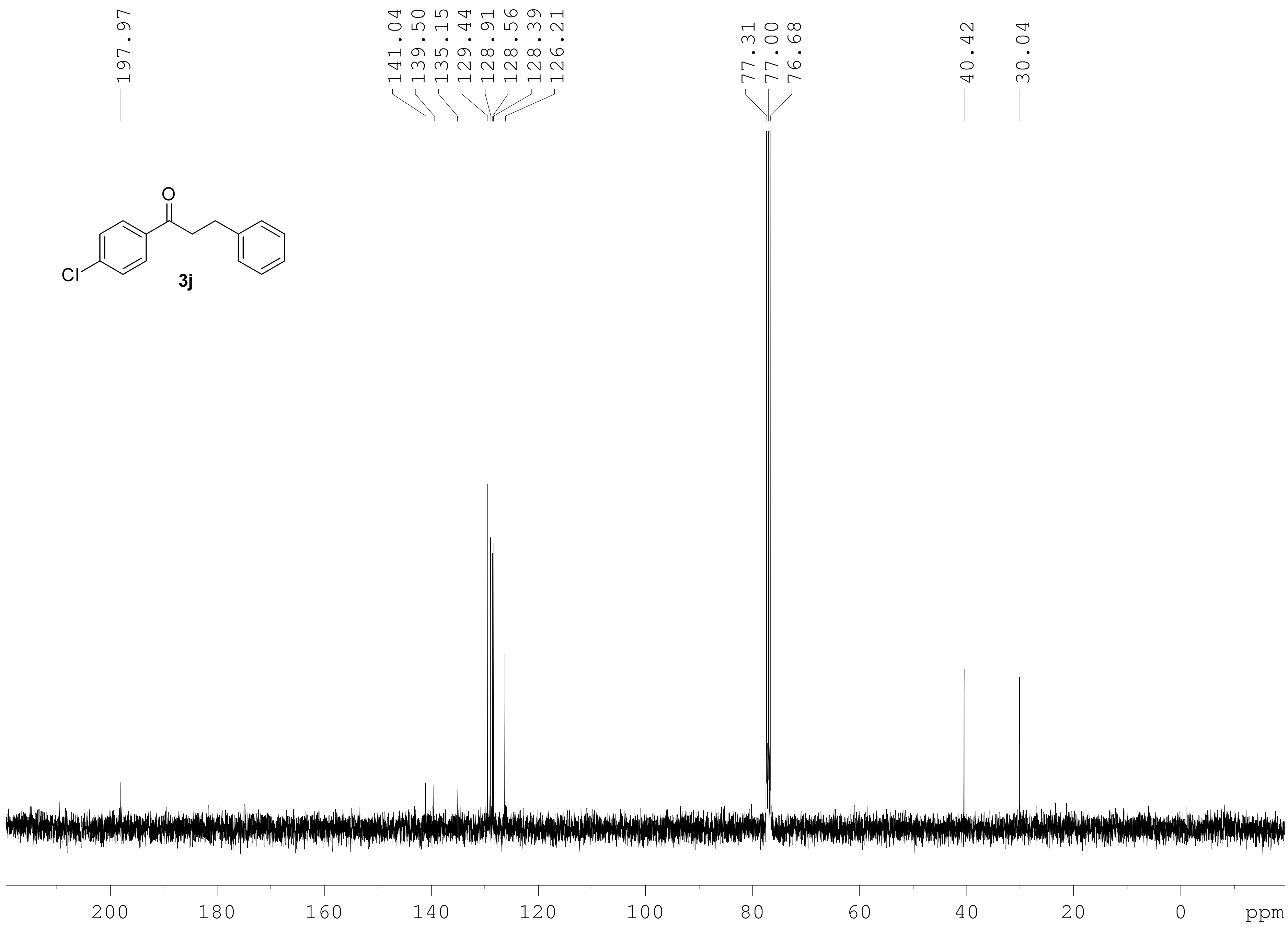

197.97
141.04
139.50
135.15
129.44
128.91
128.56
128.39
126.21
77.31
77.00
76.68
40.42
30.04
O
Cl
3j
200
180
160
140
120
100
80
60
40
20
0
ppm

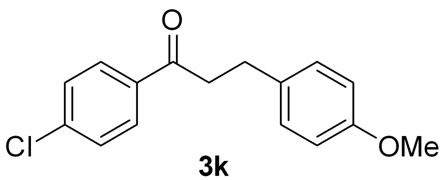
O
Cl
OMe

3k

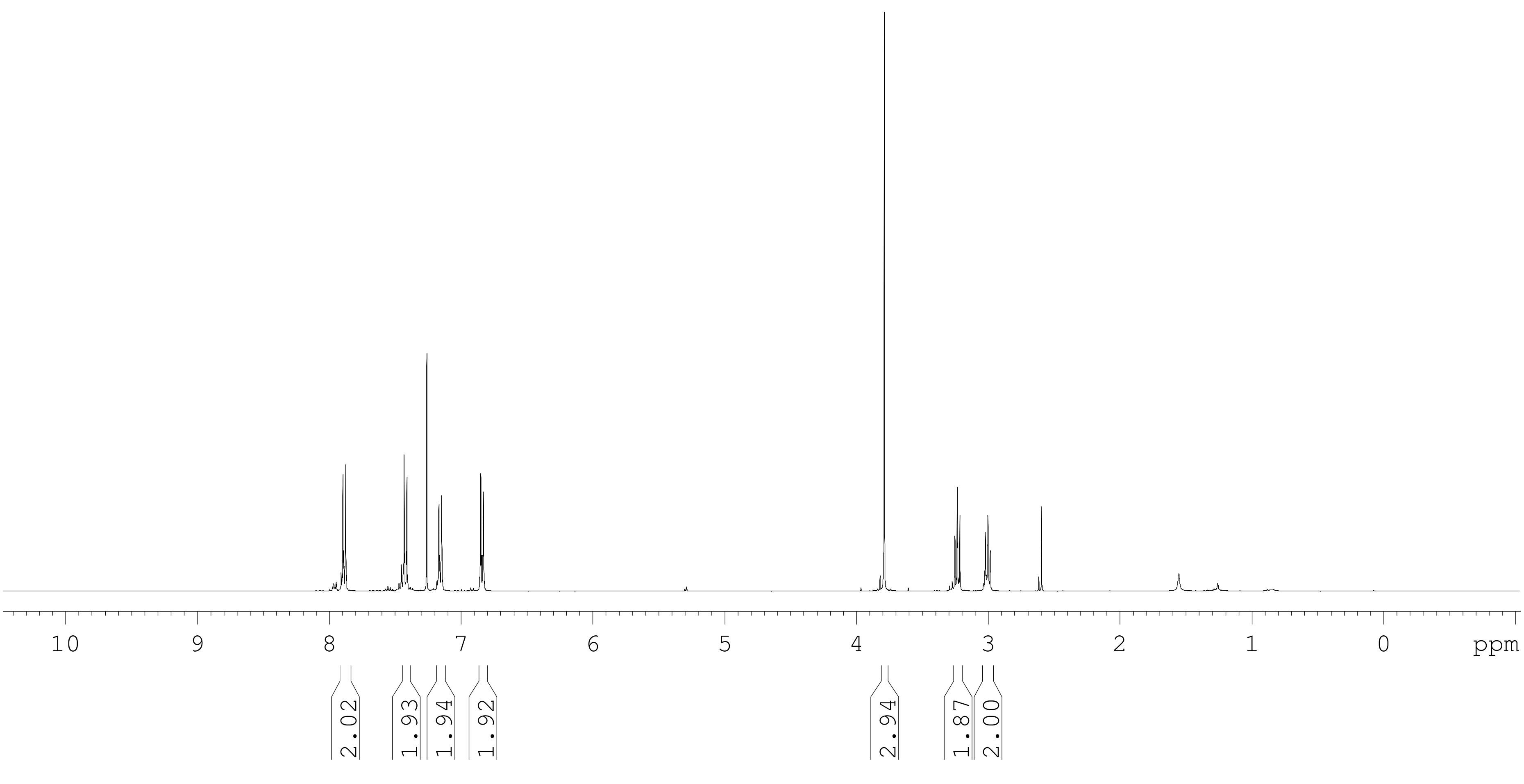
7.895
7.874
7.431
7.410
7.167
7.145
6.849
6.827
3.787
3.251
3.233
3.213
3.020
3.001
2.982
10
9
8
7
6
5
4
3
2
1
0
ppm
2.02
1.93
1.94
1.92
2.94
1.87
2.00

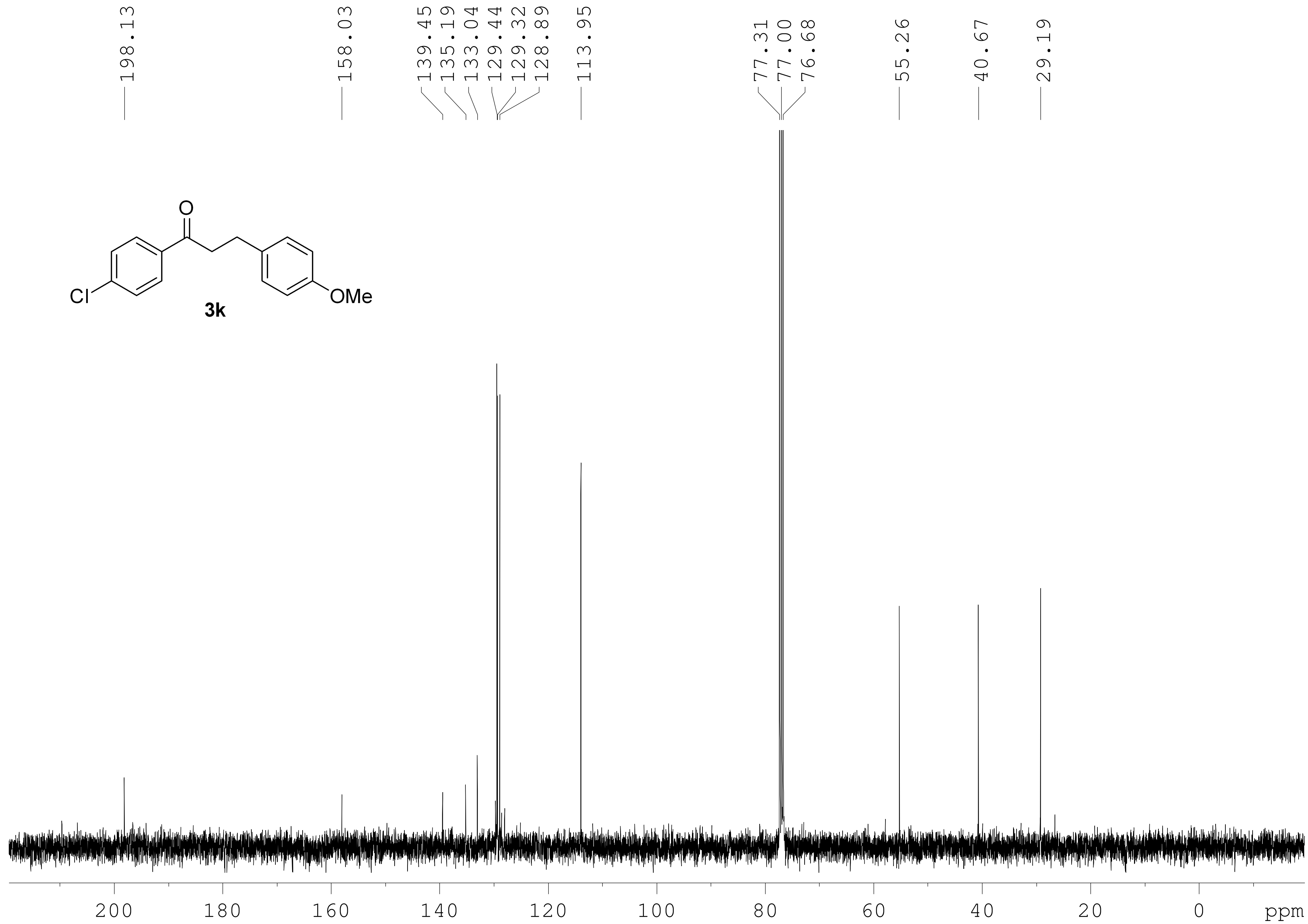
198.13
158.03
139.45
135.19
133.04
129.44
129.32
128.89
113.95
77.31
77.00
76.68
55.26
40.67
29.19
O
Cl
OMe
3k
200
180
160
140
120
100
80
60
40
20
0
ppm

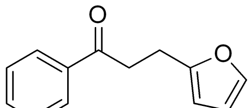

3n

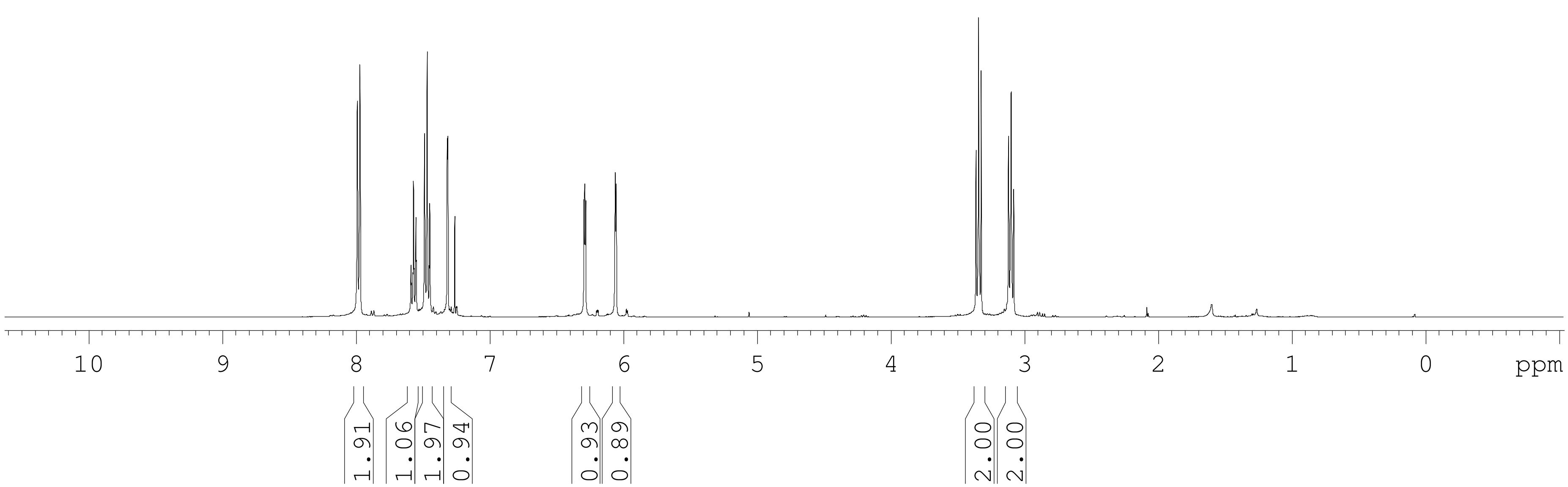
7.987
7.969
7.586
7.568
7.549
7.485
7.465
7.447
7.315
7.314
7.311
6.292
6.287
6.285
6.280
6.060
6.058
6.052
6.050
3.360
3.342
3.322
3.116
3.097
3.079
10
9
8
7
6
5
4
3
2
1
0
ppm
1.91
1.06
1.97
0.94
0.93
0.89
2.00
2.00

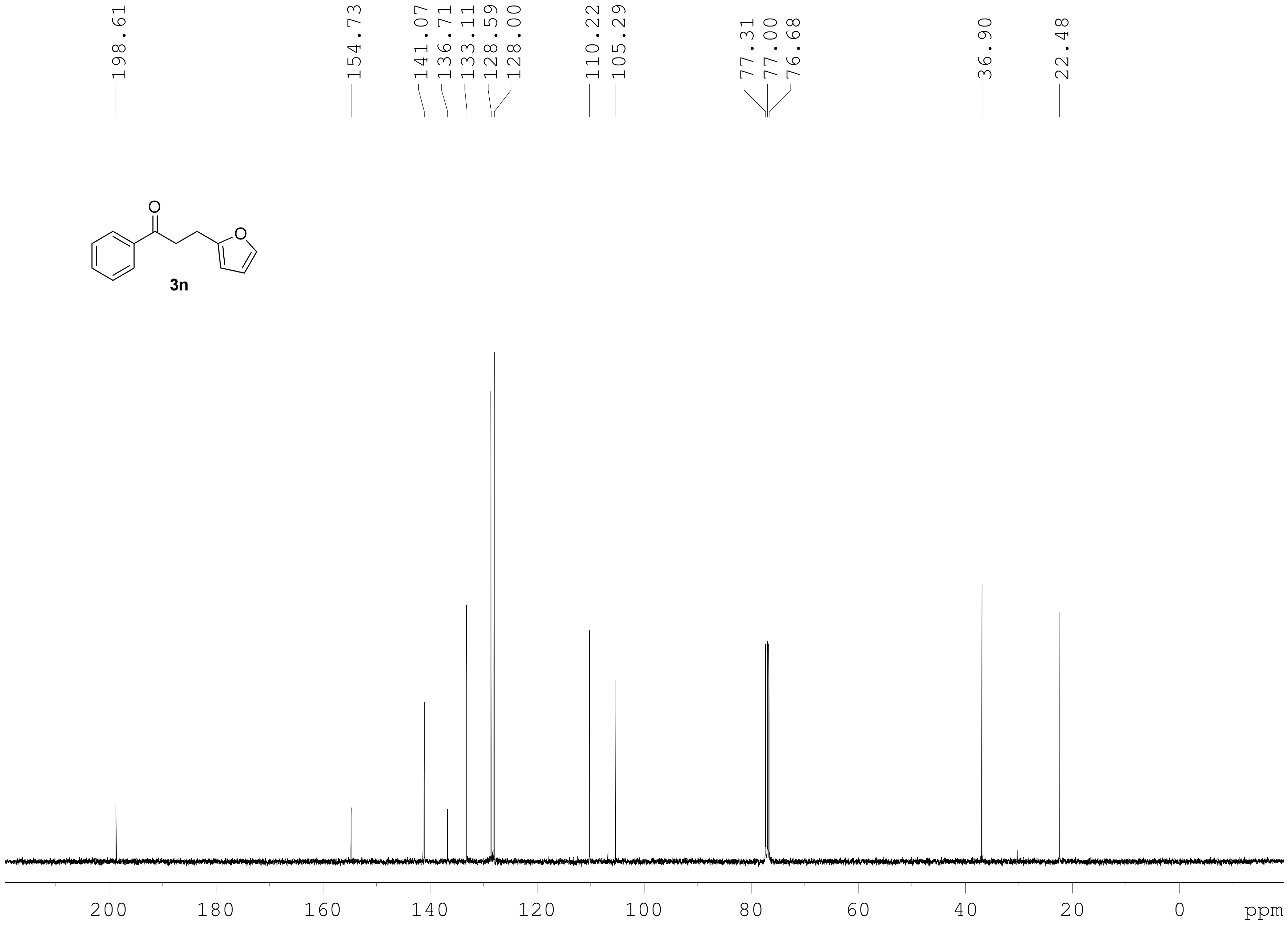

198.61
154.73
141.07
136.71
133.11
128.59
128.00
110.22
105.29
77.31
77.00
76.68
36.90
22.48
O
O
3n
200
180
160
140
120
100
80
60
40
20
0
ppm

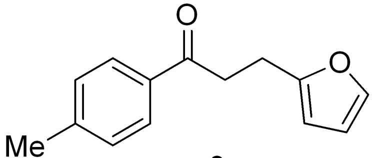
O
O
Me

3o

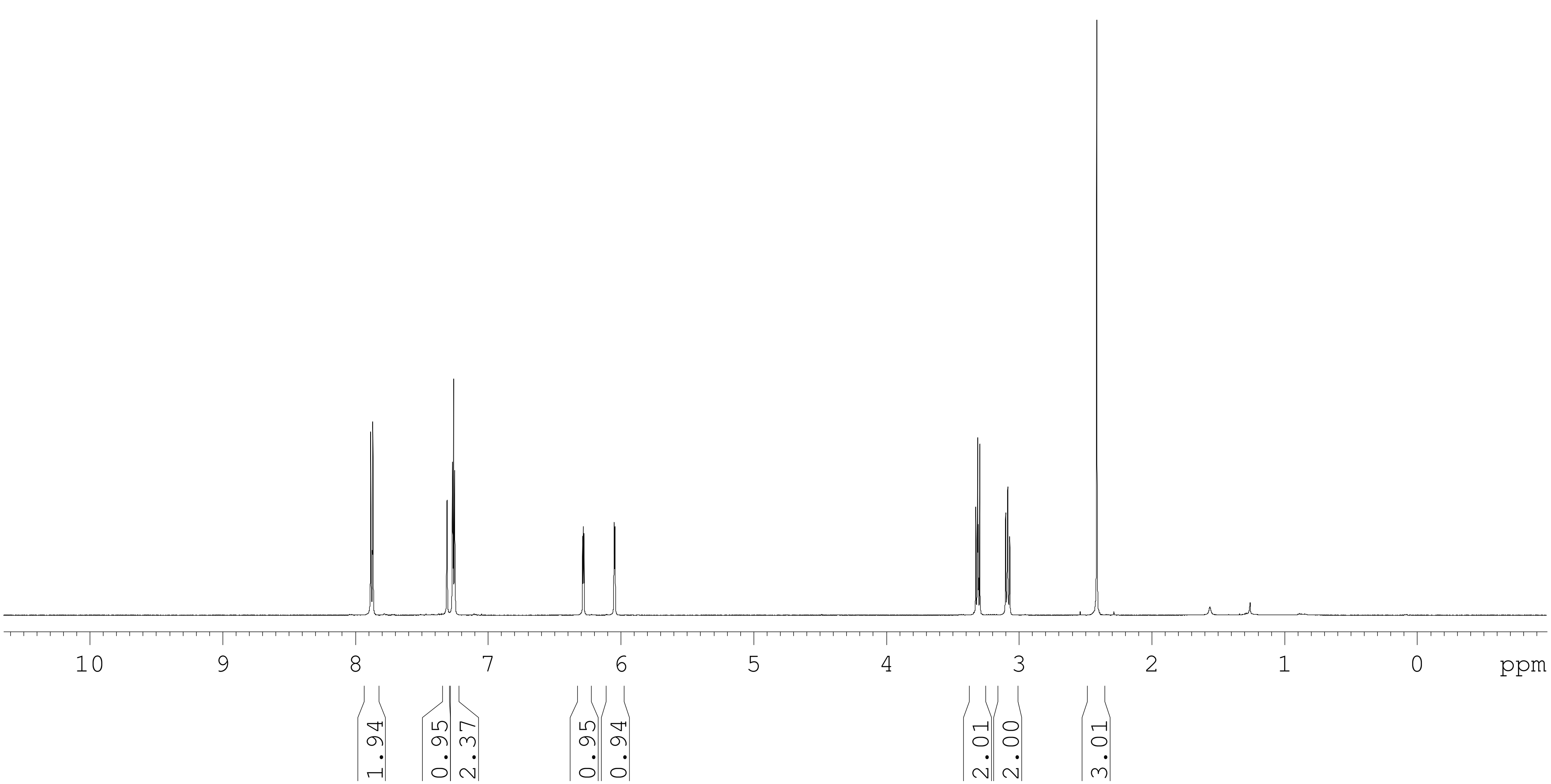
7.884
7.867
7.311
7.309
7.307
7.305
7.267
7.266
7.259
7.250
6.286
6.282
6.280
6.276
6.049
6.048
6.043
6.041
3.323
3.309
3.293
3.098
3.082
3.068
2.411
10
9
8
7
6
5
4
3
2
1
0
ppm
1.94
0.95
2.37
0.95
0.94
2.01
2.00
3.01

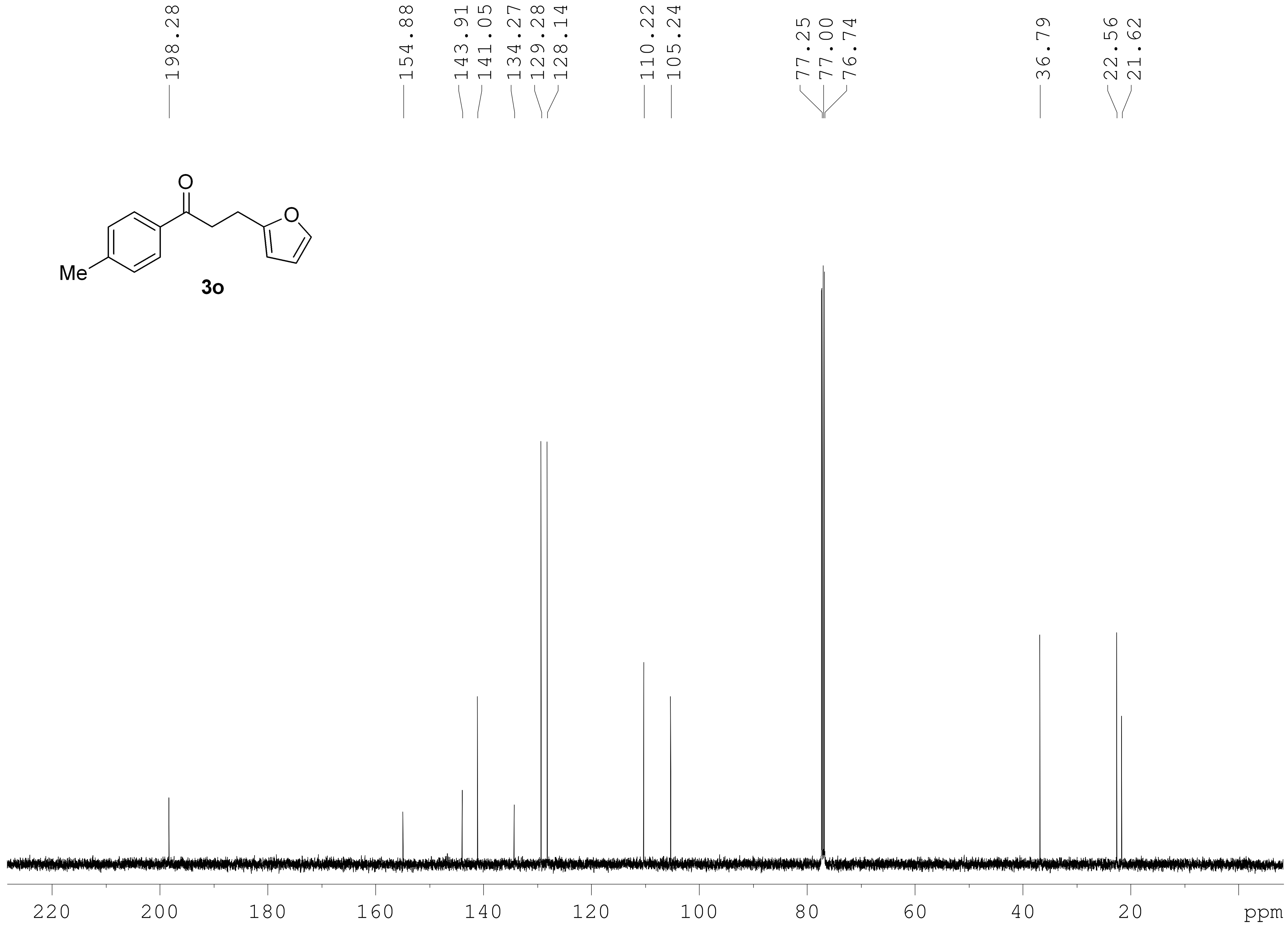

198.28
154.88
143.91
141.05
134.27
129.28
128.14
110.22
105.24
77.25
77.00
76.74
36.79
22.56
21.62
O
O
Me
3o
220
200
180
160
140
120
100
80
60
40
20
ppm

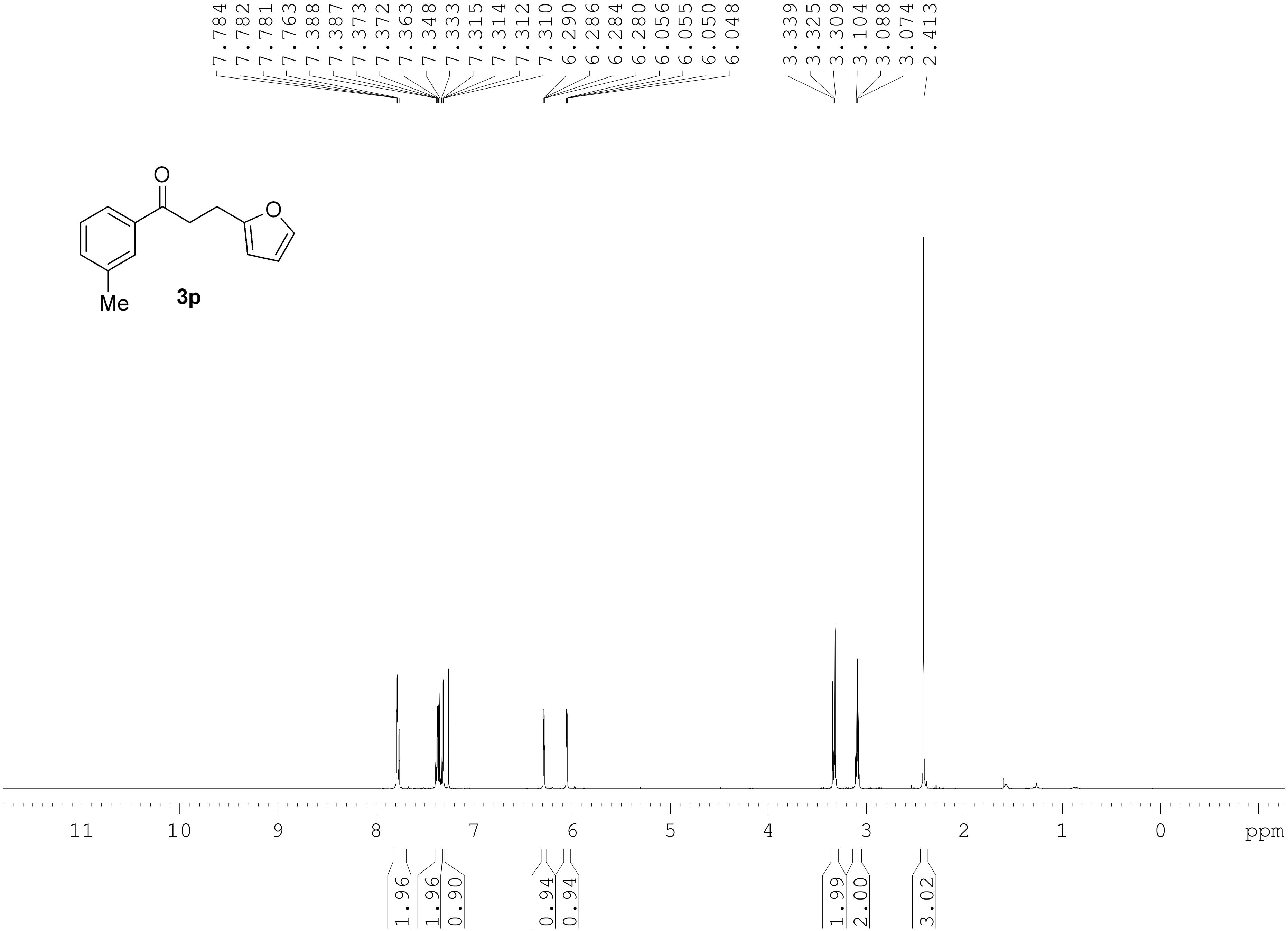
O
O
Me
3p
7.784
7.782
7.781
7.763
7.388
7.387
7.373
7.372
7.363
7.348
7.333
7.315
7.314
7.312
7.310
6.290
6.286
6.284
6.280
6.056
6.055
6.050
6.048
3.339
3.325
3.309
3.104
3.088
3.074
2.413
11
10
9
8
7
6
5
4
3
2
1
0
ppm
1.96
1.96
0.90
0.94
0.94
1.99
2.00
3.02

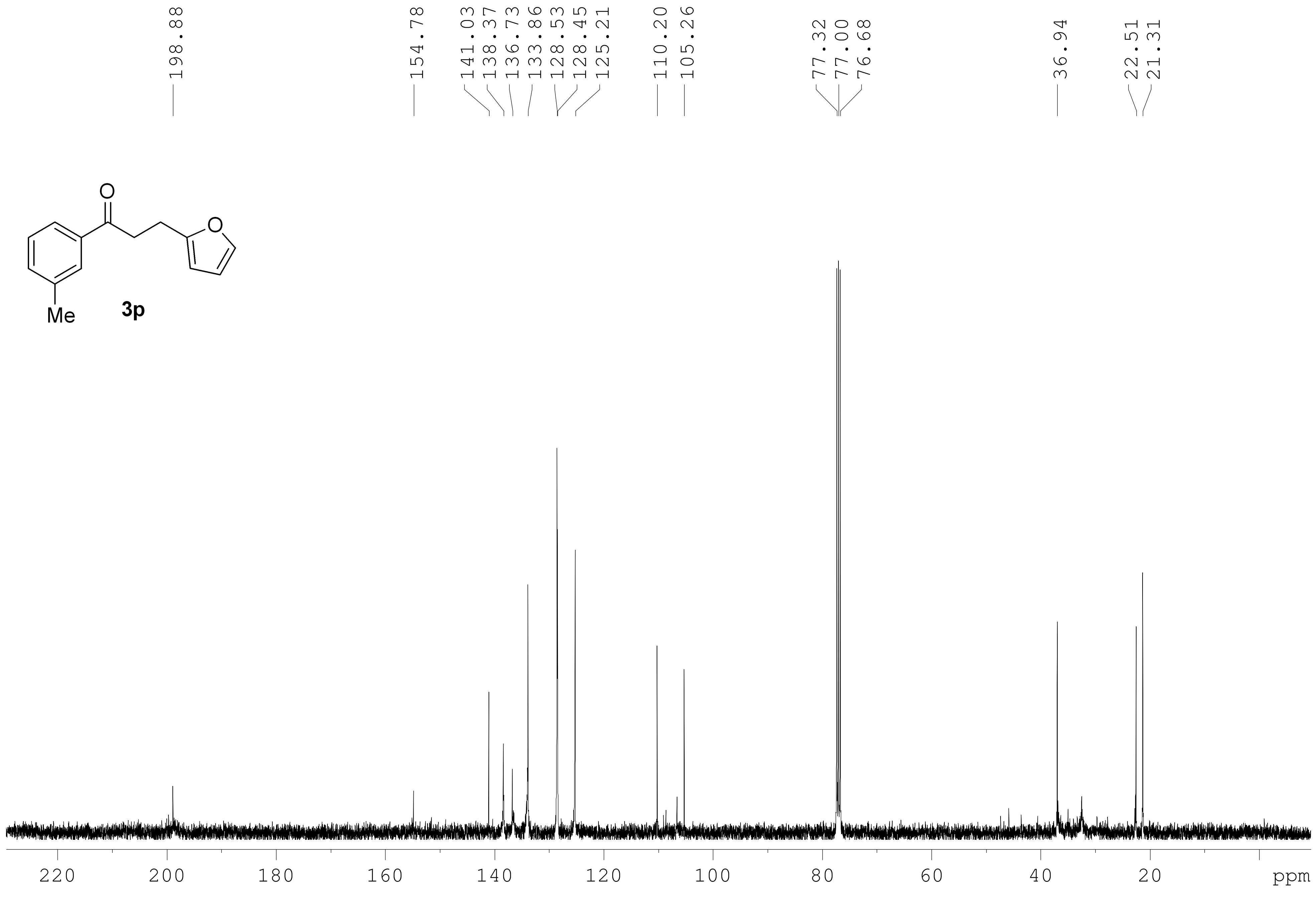
198.88
154.78
141.03
138.37
136.73
133.86
128.53
128.45
125.21
110.20
105.26
77.32
77.00
76.68
36.94
22.51
21.31
O
O
Me
3p
220
200
180
160
140
120
100
80
60
40
20
ppm

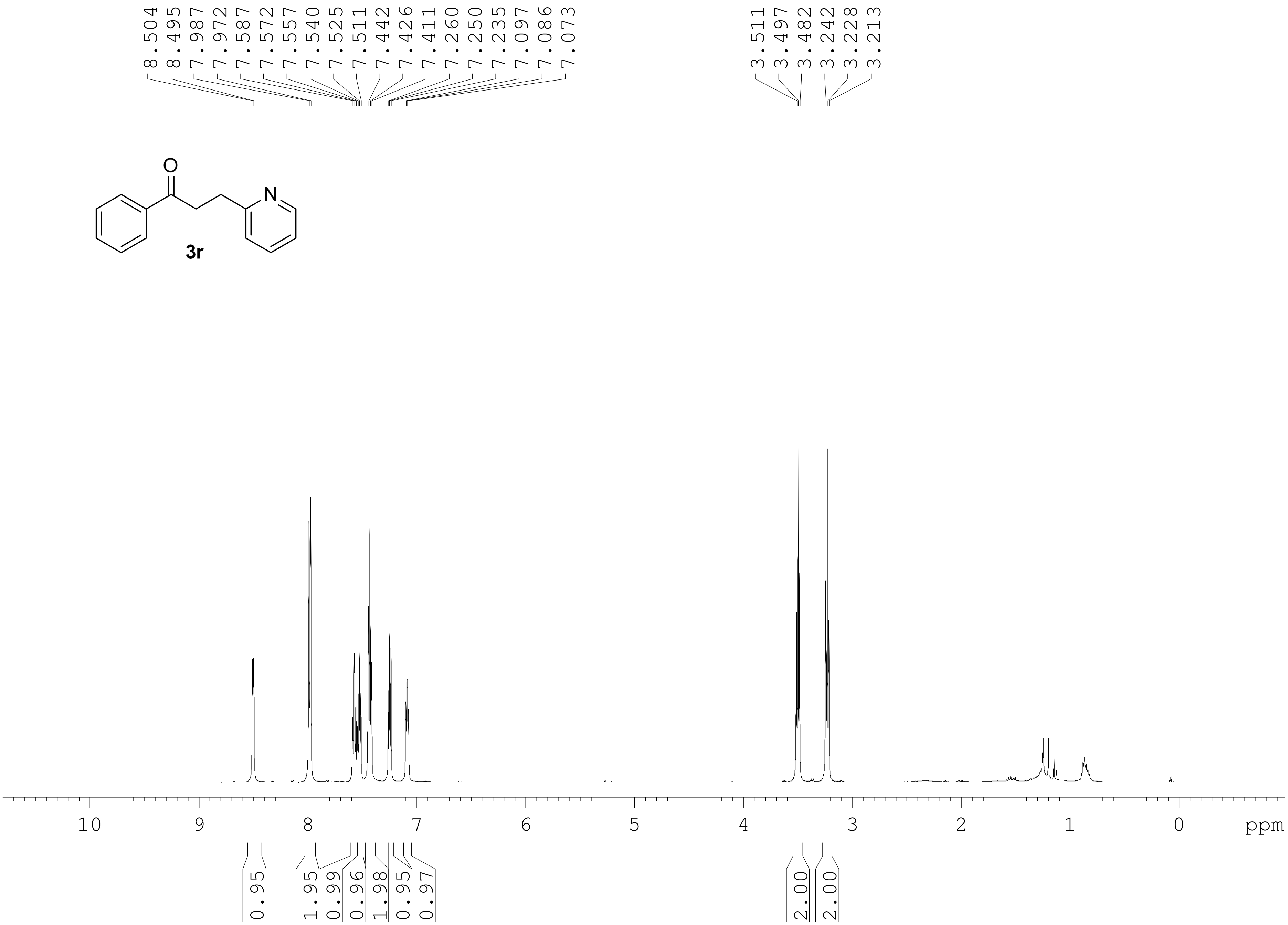

8.504
8.495
7.987
7.972
7.587
7.572
7.557
7.540
7.525
7.511
7.442
7.426
7.411
7.260
7.250
7.235
7.097
7.086
7.073
3.511
3.497
3.482
3.242
3.228
3.213
O
N
3r
10
9
8
7
6
5
4
3
2
1
0
ppm
0.95
1.95
0.99
0.96
1.98
0.95
0.97
2.00
2.00

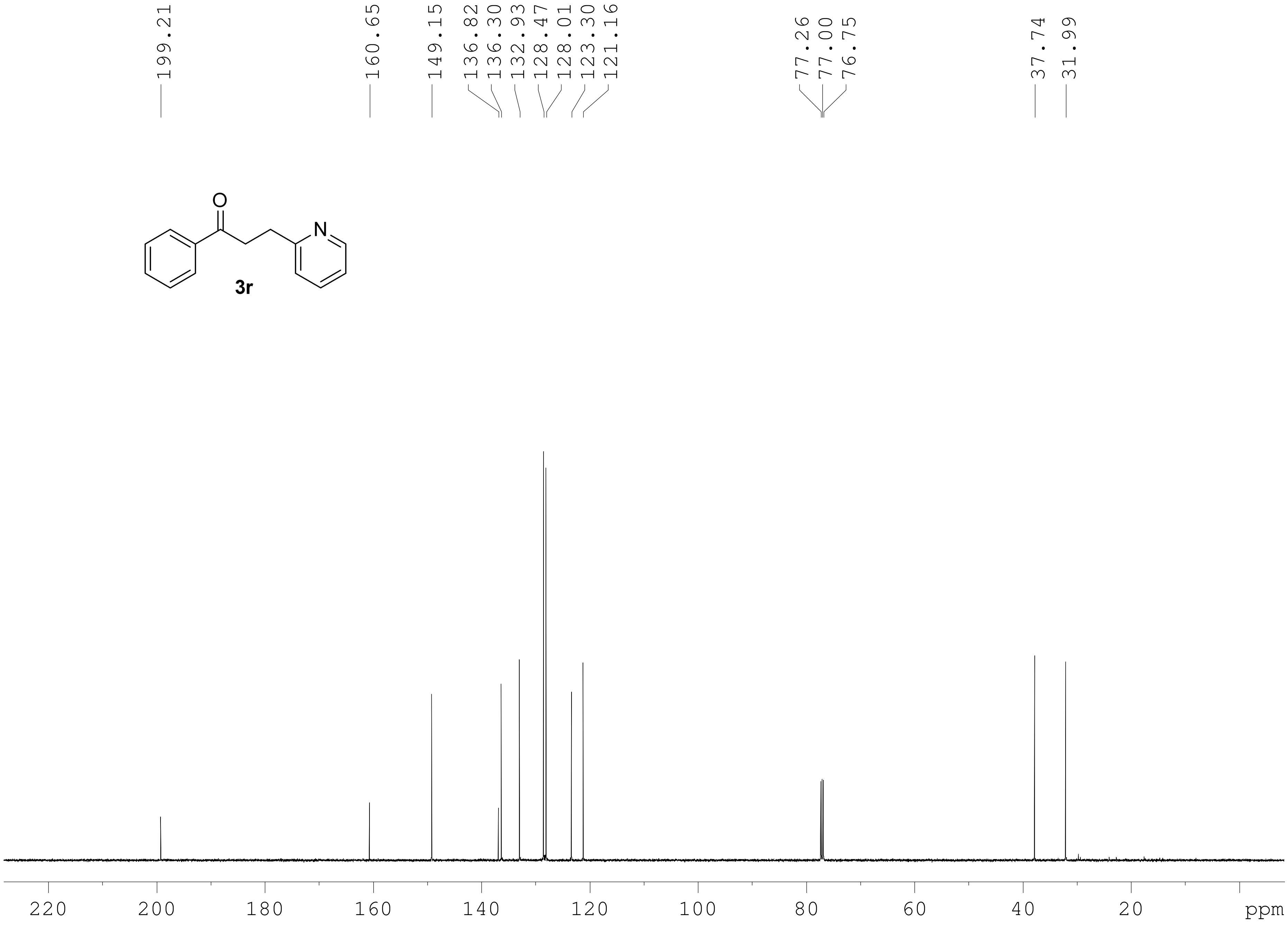
199.21
160.65
149.15
136.82
136.30
132.93
128.47
128.01
123.30
121.16
77.26
77.00
76.75
37.74
31.99
O
N
3r
220
200
180
160
140
120
100
80
60
40
20
ppm

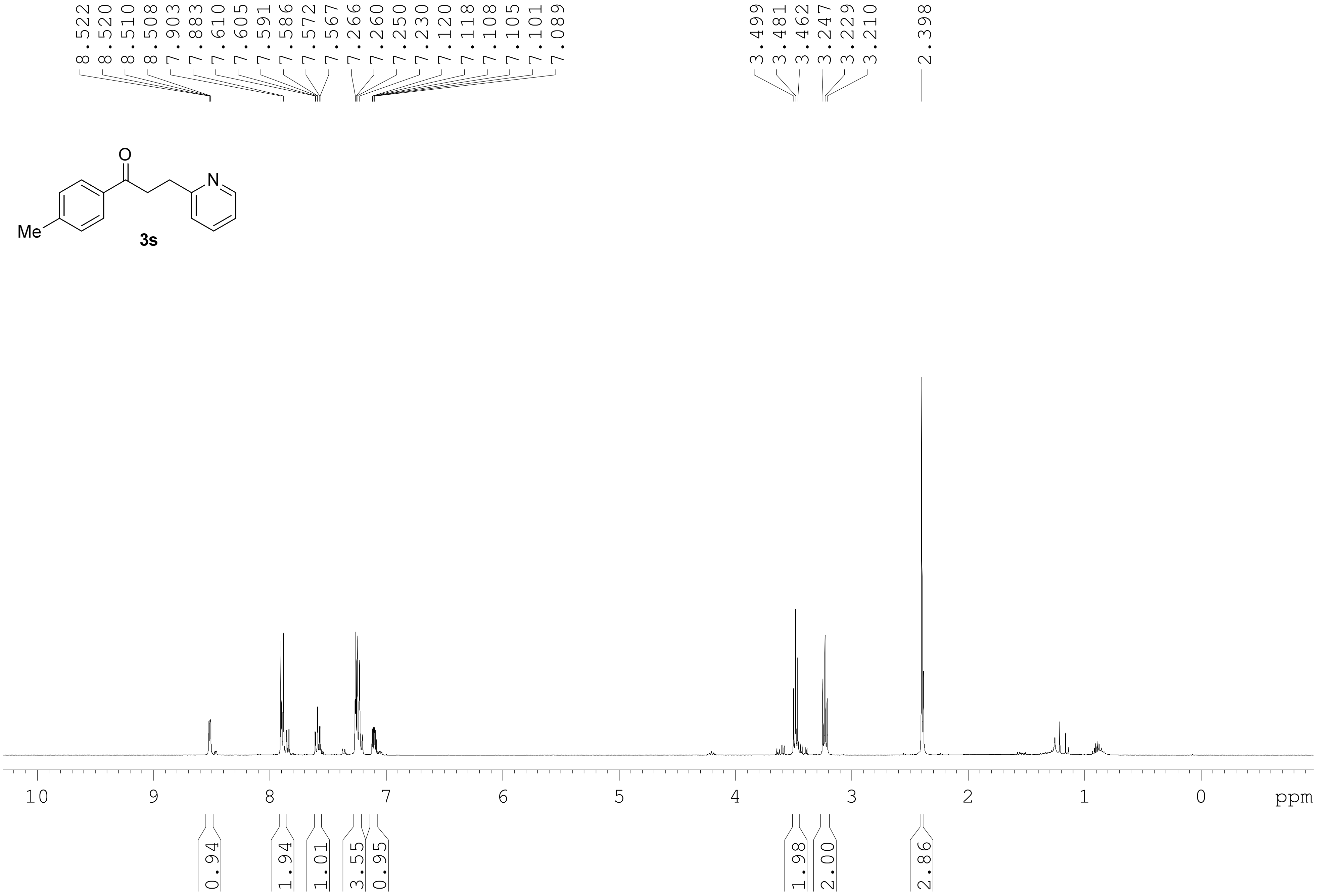
8.522 8.520 8.510 8.508 7.903 7.883 7.610 7.605 7.591 7.586 7.572 7.567 7.266 7.260 7.250 7.230 7.120 7.118 7.108 7.105 7.101 7.089
3.499 3.481 3.462 3.247 3.229 3.210
2.398
O
N
Me
3s
10 9 8 7 6 5 4 3 2 1 0 ppm
0.94 1.94 1.01 3.55 0.95 1.98 2.00 2.86

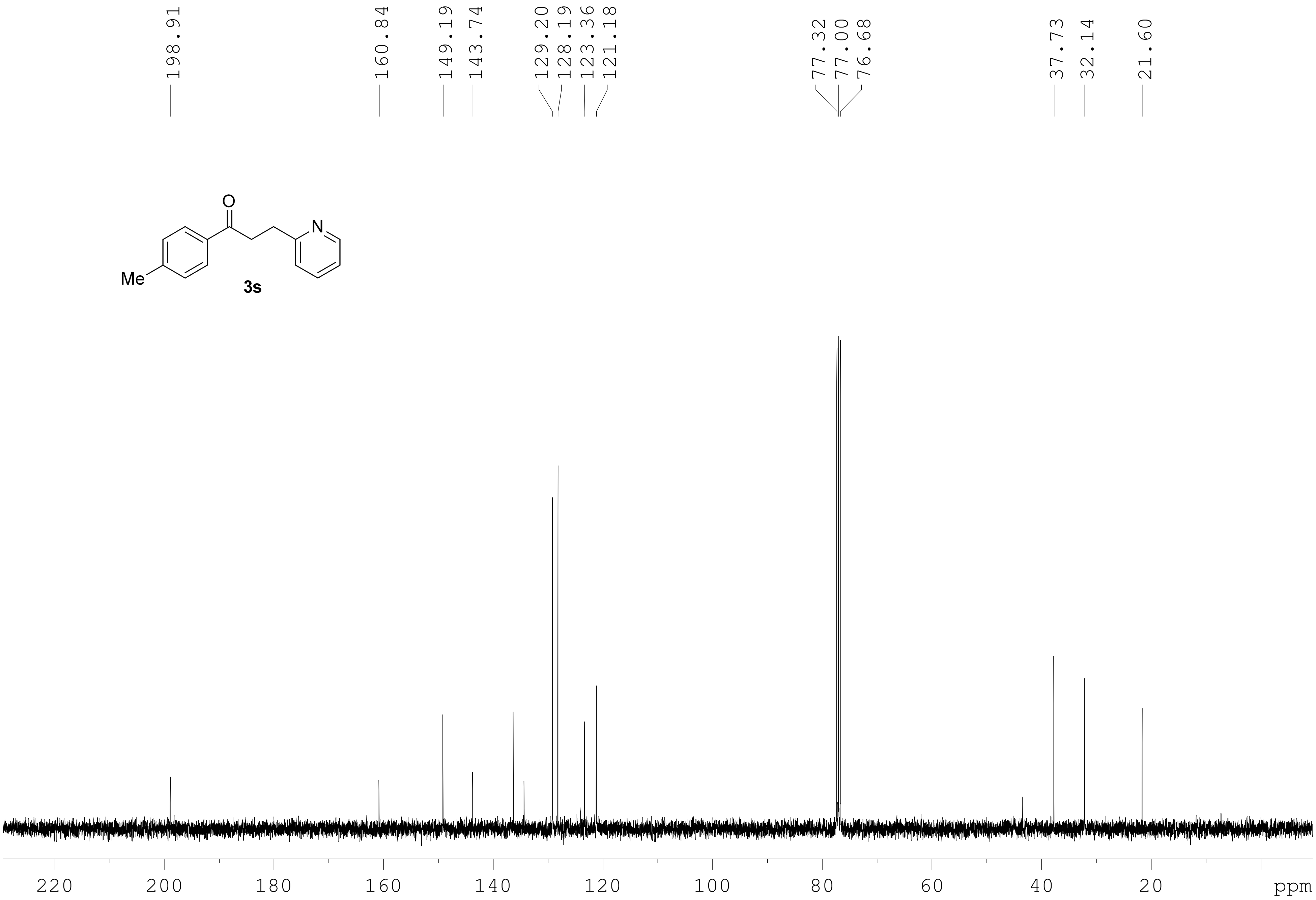
198.91
160.84
149.19
143.74
129.20
128.19
123.36
121.18
77.32
77.00
76.68
37.73
32.14
21.60
O
N
Me
3s
220
200
180
160
140
120
100
80
60
40
20
ppm

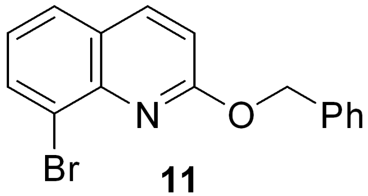
Br
N
O
Ph

11

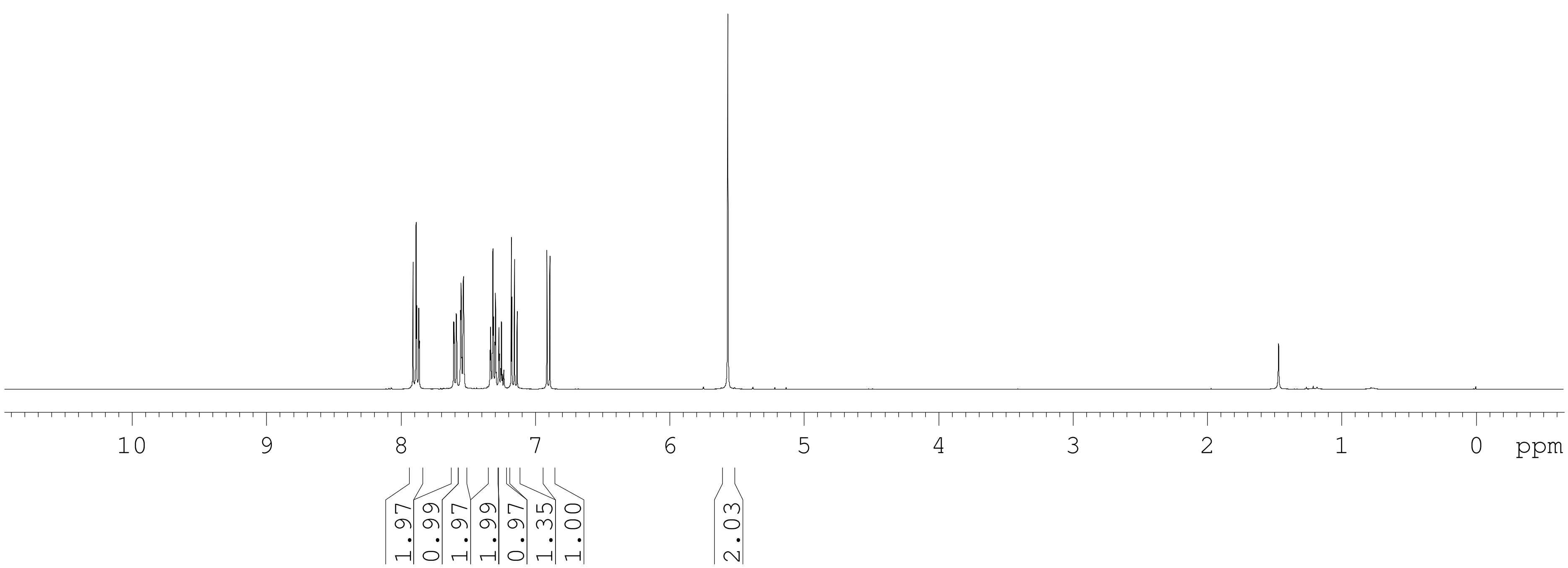
7.908
7.886
7.881
7.866
7.862
7.606
7.603
7.586
7.583
7.555
7.551
7.546
7.534
7.335
7.332
7.314
7.310
7.299
7.295
7.272
7.269
7.265
7.250
7.177
7.173
7.153
7.134
6.912
6.890
5.566
10
9
8
7
6
5
4
3
2
1
0
ppm
1.97
0.99
1.97
1.99
0.97
1.35
1.00
2.03

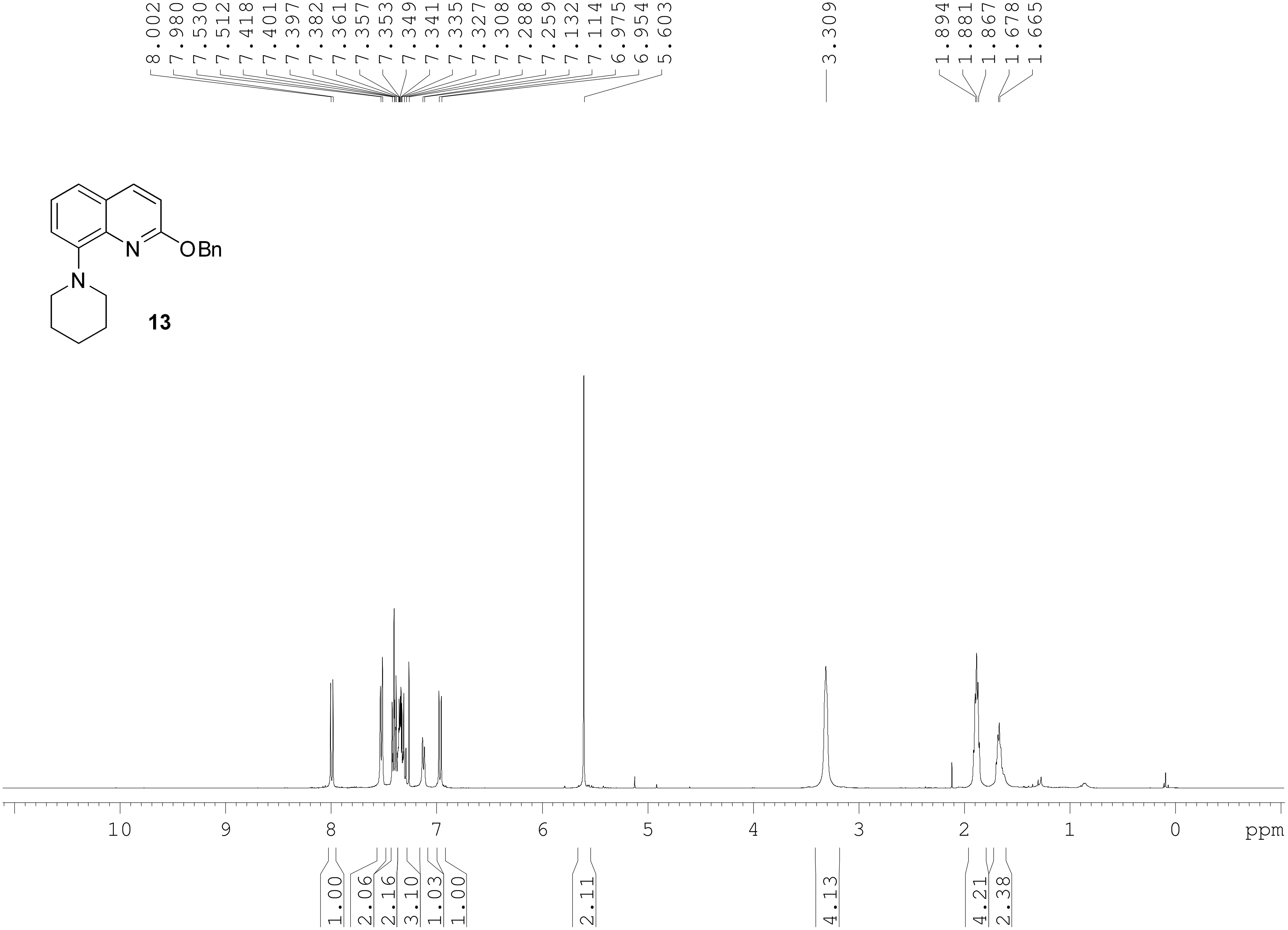
8.002
7.980
7.530
7.512
7.418
7.401
7.397
7.382
7.361
7.357
7.353
7.349
7.341
7.335
7.327
7.308
7.288
7.259
7.132
7.114
6.975
6.954
5.603
3.309
1.894
1.881
1.867
1.678
1.665
N
OBn
N
13
10
9
8
7
6
5
4
3
2
1
0
ppm
1.00
2.06
2.16
3.10
1.03
1.00
2.11
4.13
4.21
2.38

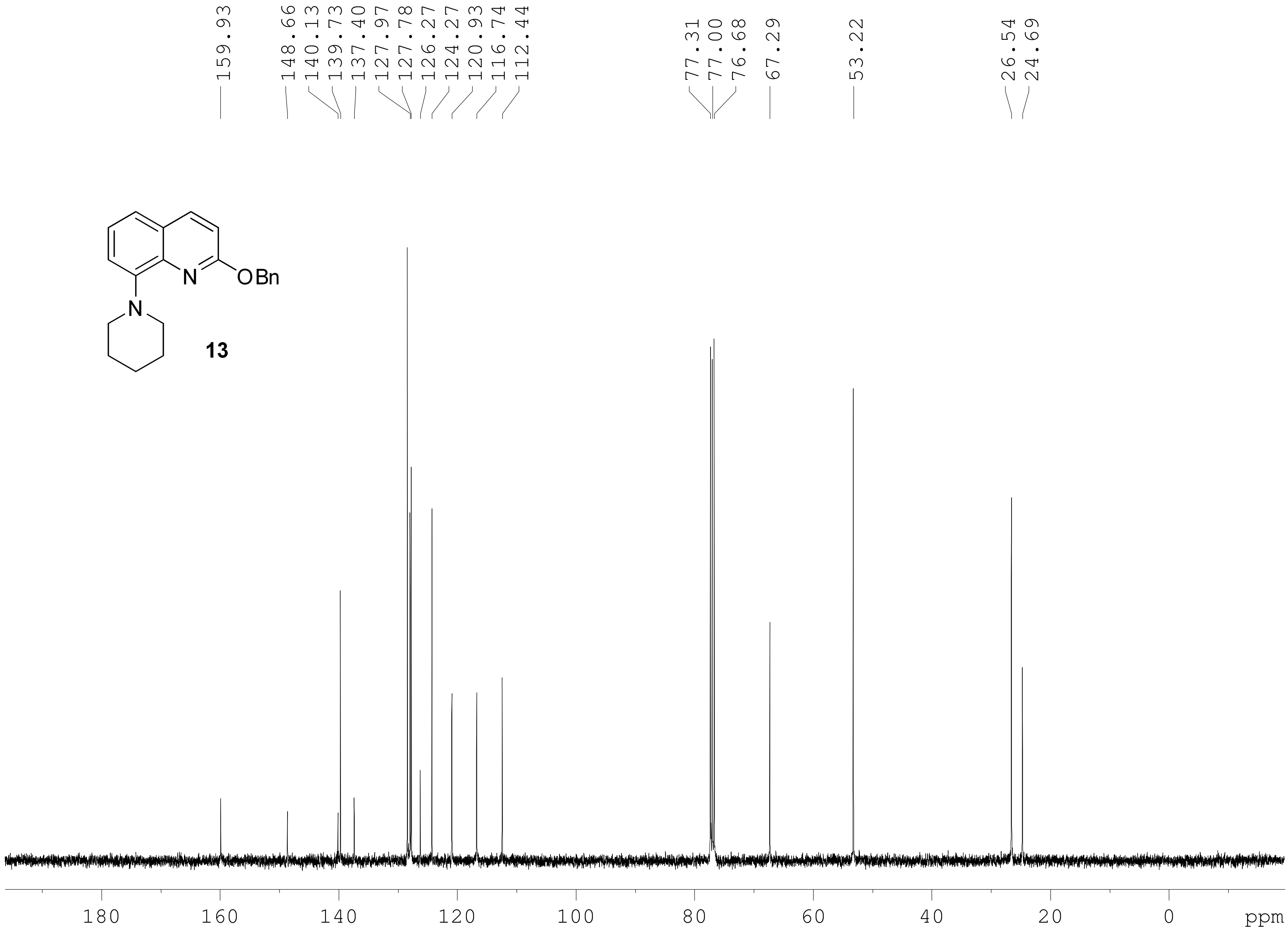

159.93
148.66
140.13
139.73
137.40
127.97
127.78
126.27
124.27
120.93
116.74
112.44
77.31
77.00
76.68
67.29
53.22
26.54
24.69
N
OBn
N
13
180
160
140
120
100
80
60
40
20
0
ppm

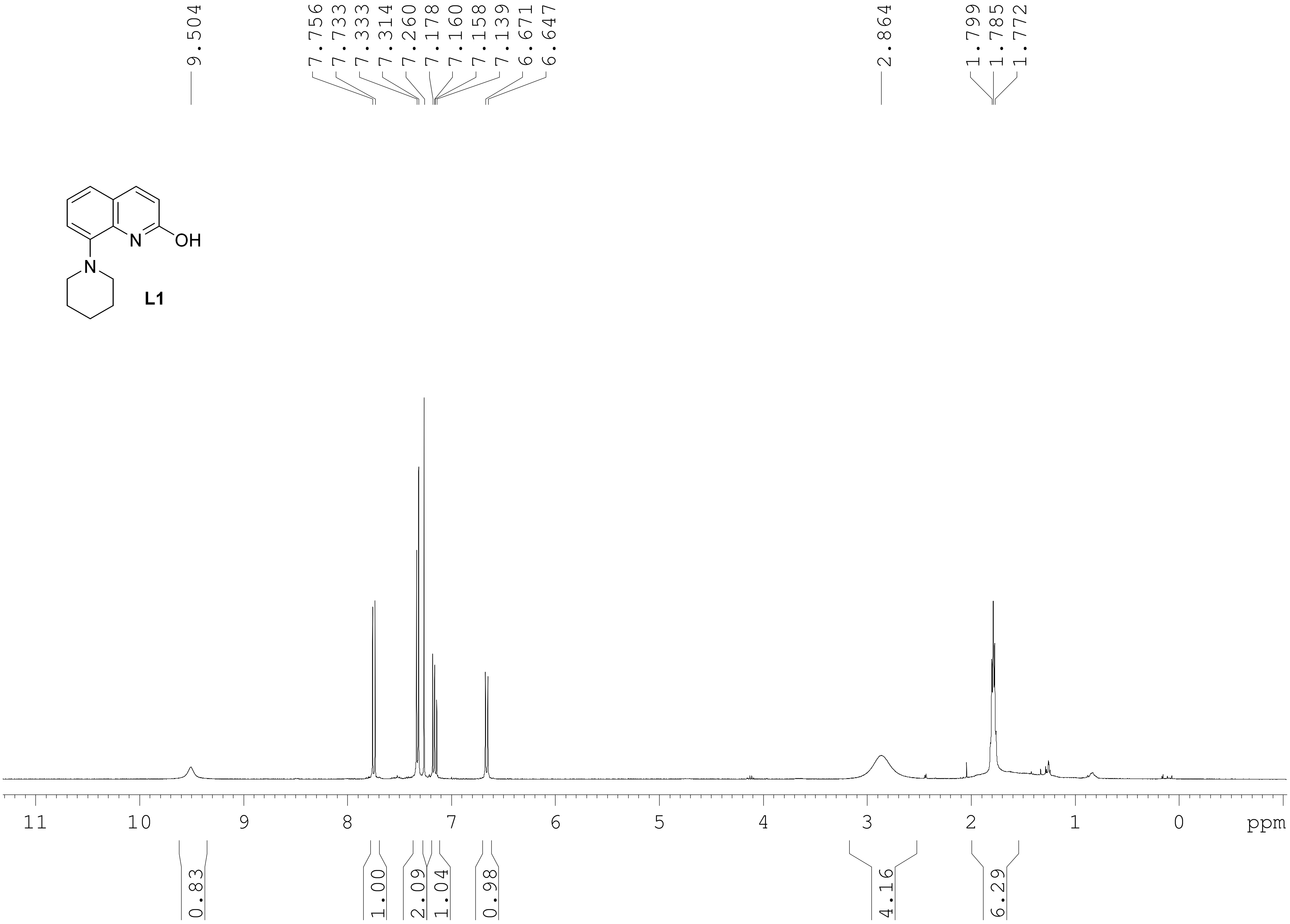
N
OH
L1
9.504
7.756
7.733
7.333
7.314
7.260
7.178
7.160
7.158
7.139
6.671
6.647
2.864
1.799
1.785
1.772
11
10
9
8
7
6
5
4
3
2
1
0
ppm
0.83
1.00
2.09
1.04
0.98
4.16
6.29

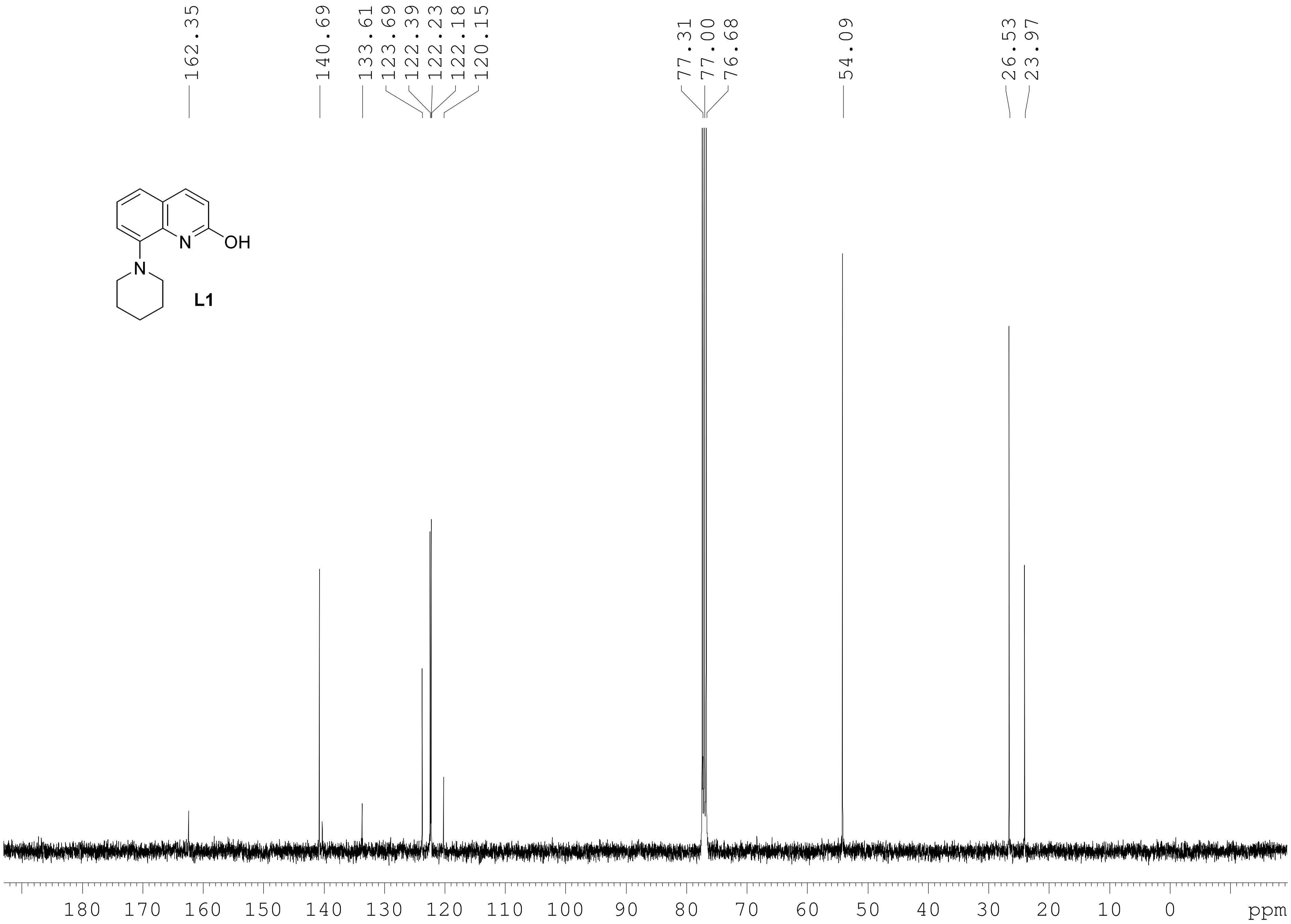
162.35
140.69
133.61
123.69
122.39
122.23
122.18
120.15
77.31
77.00
76.68
54.09
26.53
23.97
L1
180 170 160 150 140 130 120 110 100 90 80 70 60 50 40 30 20 10 0 ppm

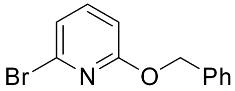
Br
N
O
Ph

15

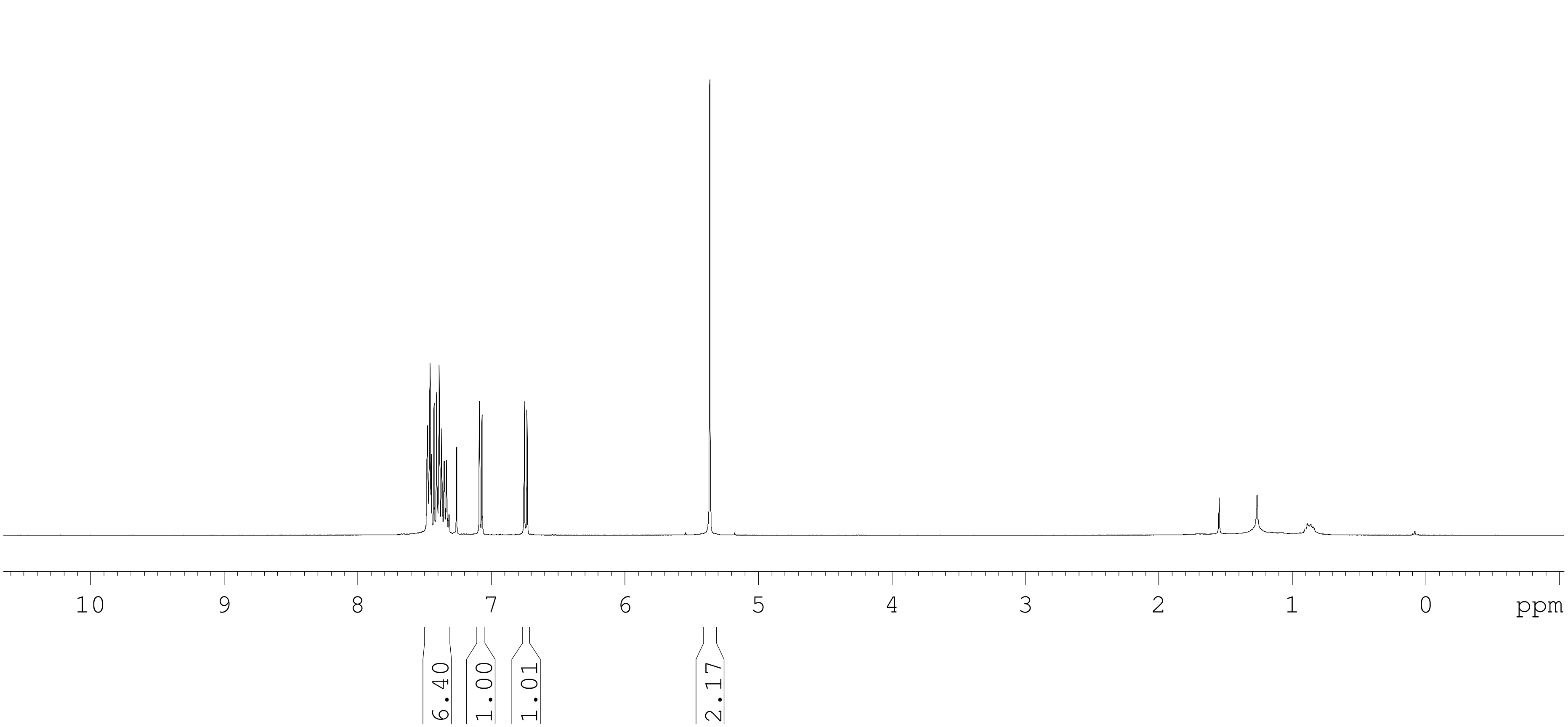
7.502
7.484
7.473
7.454
7.434
7.415
7.396
7.285
7.113
7.095
6.778
6.757
5.388
10
9
8
7
6
5
4
3
2
1
0
ppm
6.40
1.00
1.01
2.17

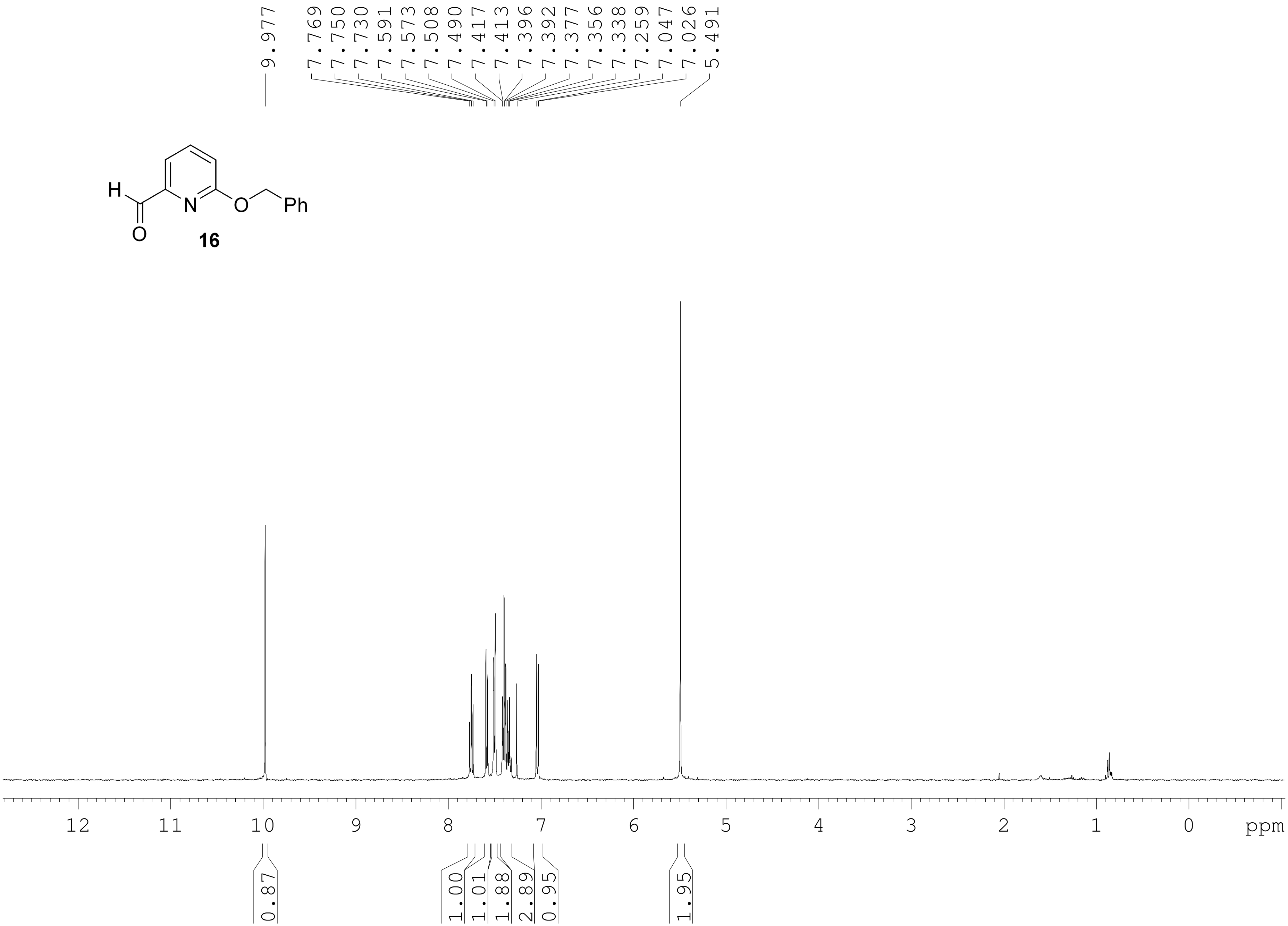
9.977
7.769
7.750
7.730
7.591
7.573
7.508
7.490
7.417
7.413
7.396
7.392
7.377
7.356
7.338
7.259
7.047
7.026
5.491
16
12
11
10
9
8
7
6
5
4
3
2
1
0
ppm
0.87
1.00
1.01
1.88
2.89
0.95
1.95

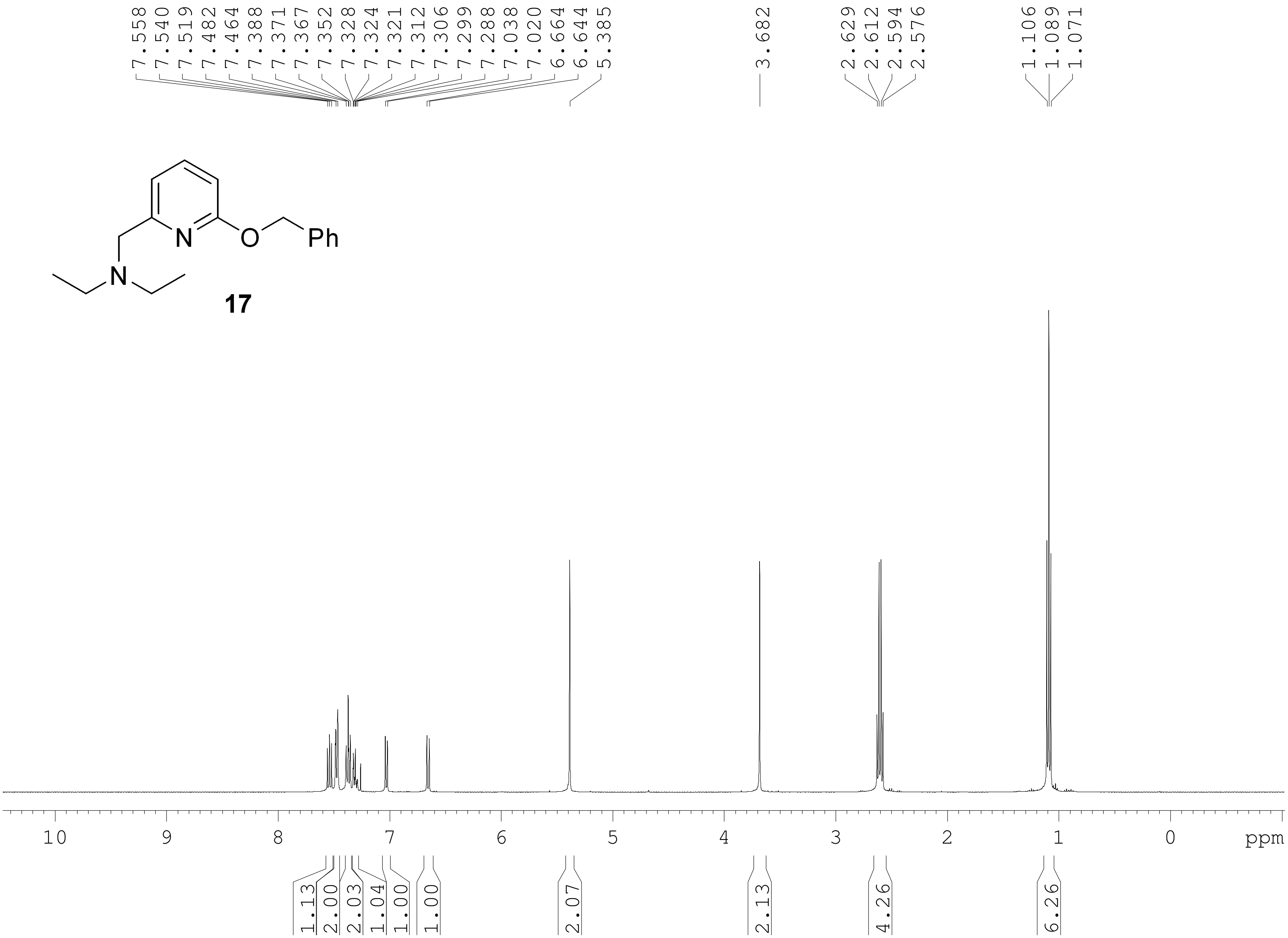
7.558
7.540
7.519
7.482
7.464
7.388
7.371
7.367
7.352
7.328
7.324
7.321
7.312
7.306
7.299
7.288
7.038
7.020
6.664
6.644
5.385
3.682
2.629
2.612
2.594
2.576
1.106
1.089
1.071
N
O
Ph
N
17
10
9
8
7
6
5
4
3
2
1
0
ppm
1.13
2.00
2.03
1.04
1.00
1.00
2.07
2.13
4.26
6.26

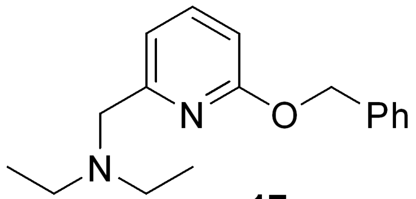
N
O
Ph
N

17

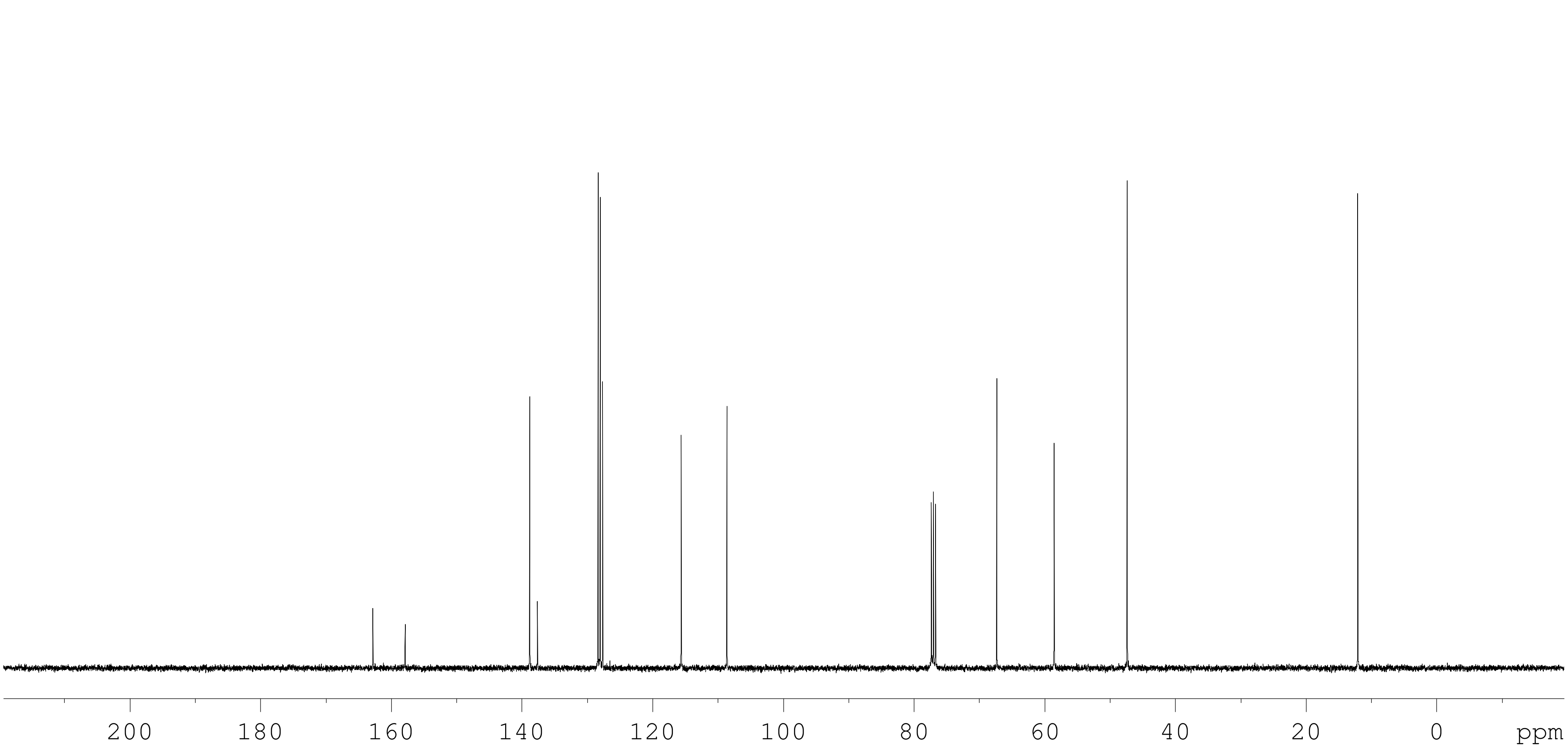
162.82
157.86
138.77
137.64
128.33
128.01
127.64
126.53
115.62
108.61
77.32
77.00
76.68
67.29
58.53
47.33
12.05
200
180
160
140
120
100
80
60
40
20
0
ppm

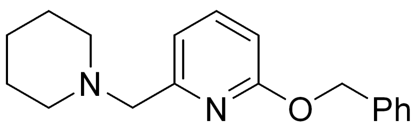

18

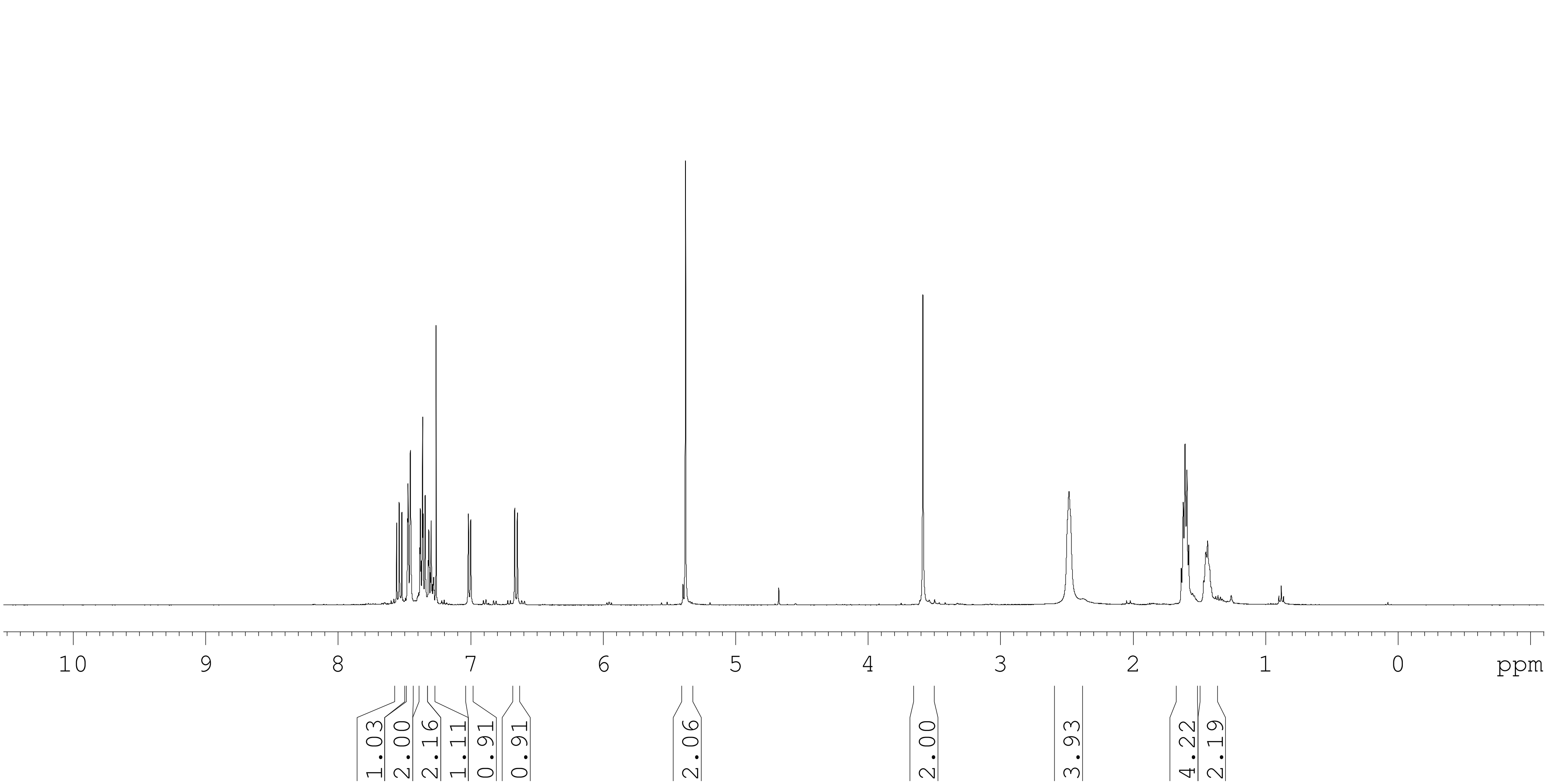

7.557
7.538
7.536
7.518
7.475
7.472
7.455
7.453
7.382
7.378
7.361
7.357
7.342
7.318
7.314
7.311
7.302
7.296
7.290
7.282
7.278
7.260
7.016
6.998
6.666
6.646
5.377
3.585
2.482
1.634
1.620
1.606
1.592
1.578
1.449
1.436
10
9
8
7
6
5
4
3
2
1
0
ppm
1.03
2.00
2.16
1.11
0.91
0.91
2.06
2.00
3.93
4.22
2.19

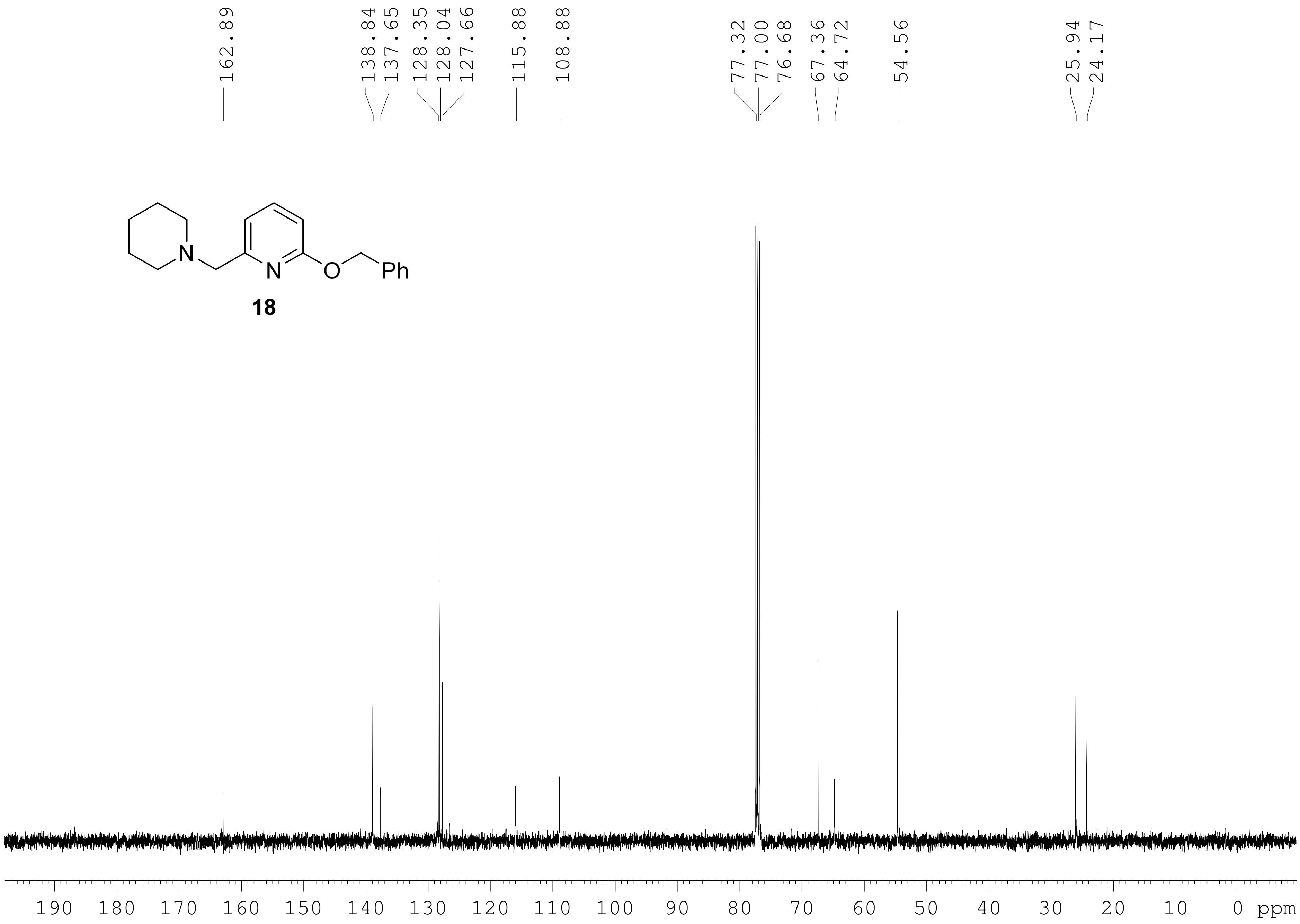
162.89
138.84
137.65
128.35
128.04
127.66
115.88
108.88
77.32
77.00
76.68
67.36
64.72
54.56
25.94
24.17
N
N
O
Ph
18
190
180
170
160
150
140
130
120
110
100
90
80
70
60
50
40
30
20
10
0 ppm

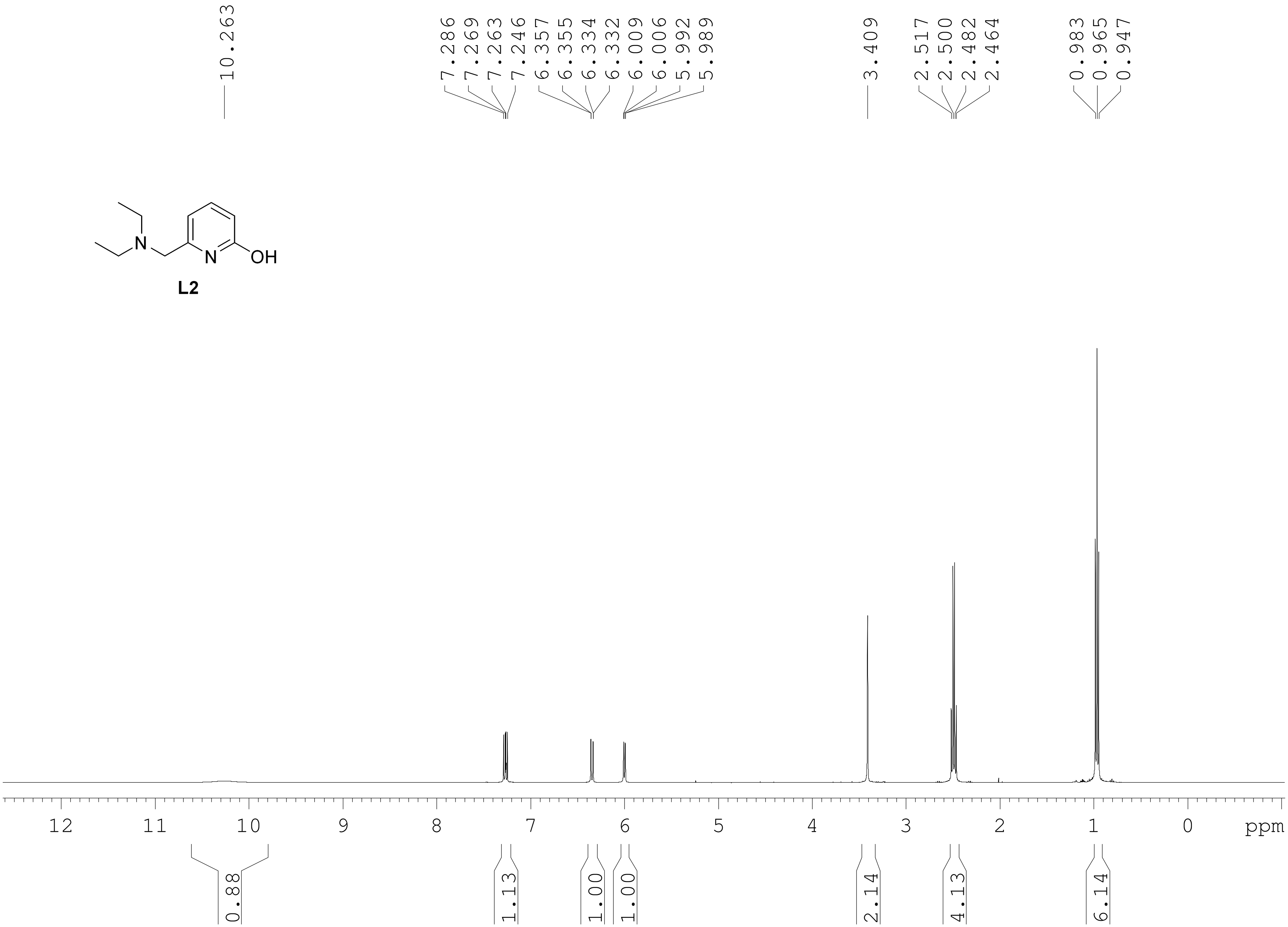

10.263
7.286
7.269
7.263
7.246
6.357
6.355
6.334
6.332
6.009
6.006
5.992
5.989
3.409
2.517
2.500
2.482
2.464
0.983
0.965
0.947
N
OH
L2
12
11
10
9
8
7
6
5
4
3
2
1
0
ppm
0.88
1.13
1.00
1.00
2.14
4.13
6.14

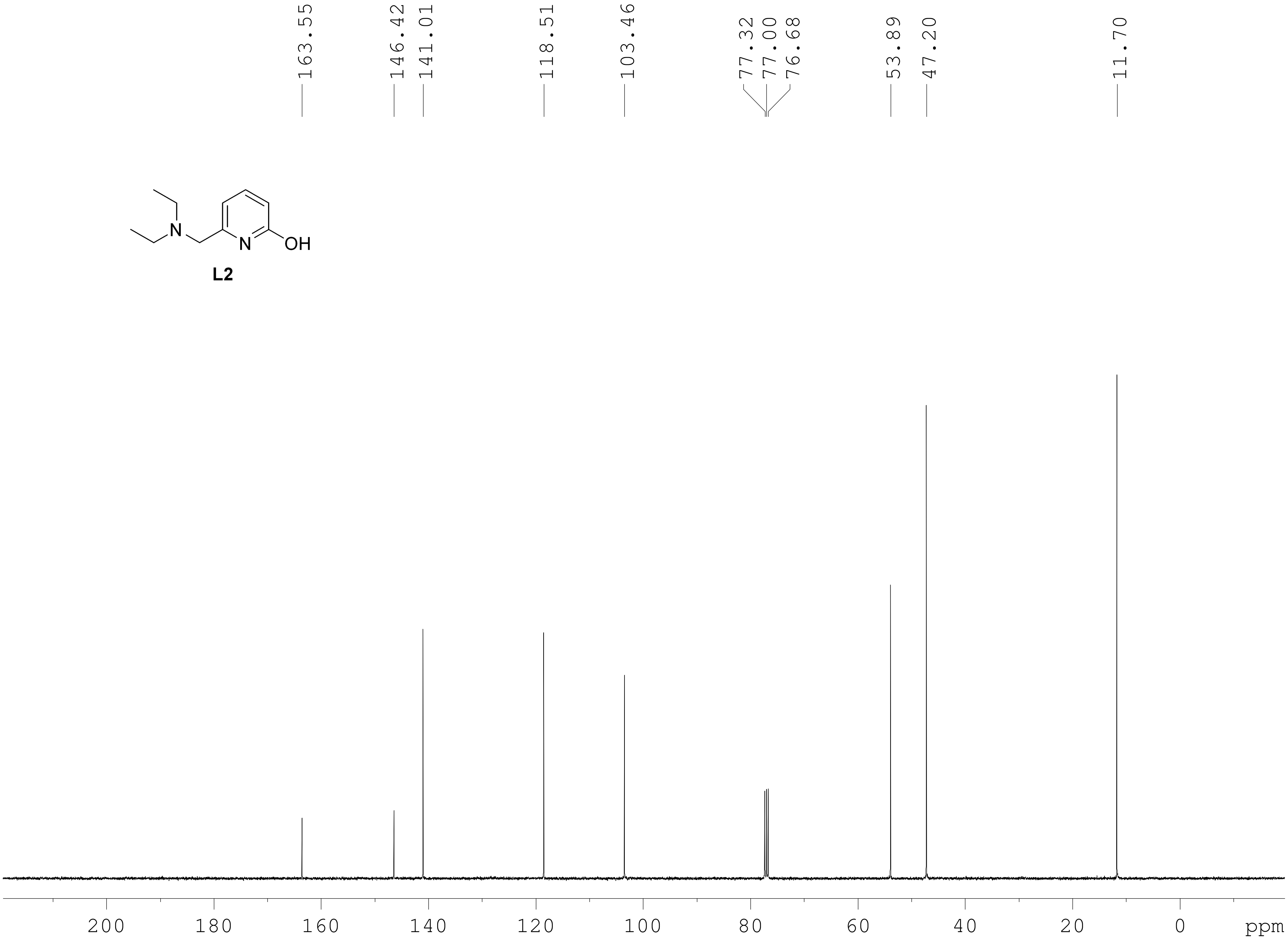
163.55
146.42
141.01
118.51
103.46
77.32
77.00
76.68
53.89
47.20
11.70
200
180
160
140
120
100
80
60
40
20
0
ppm
N
N
OH

L2

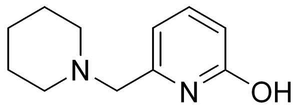

L3

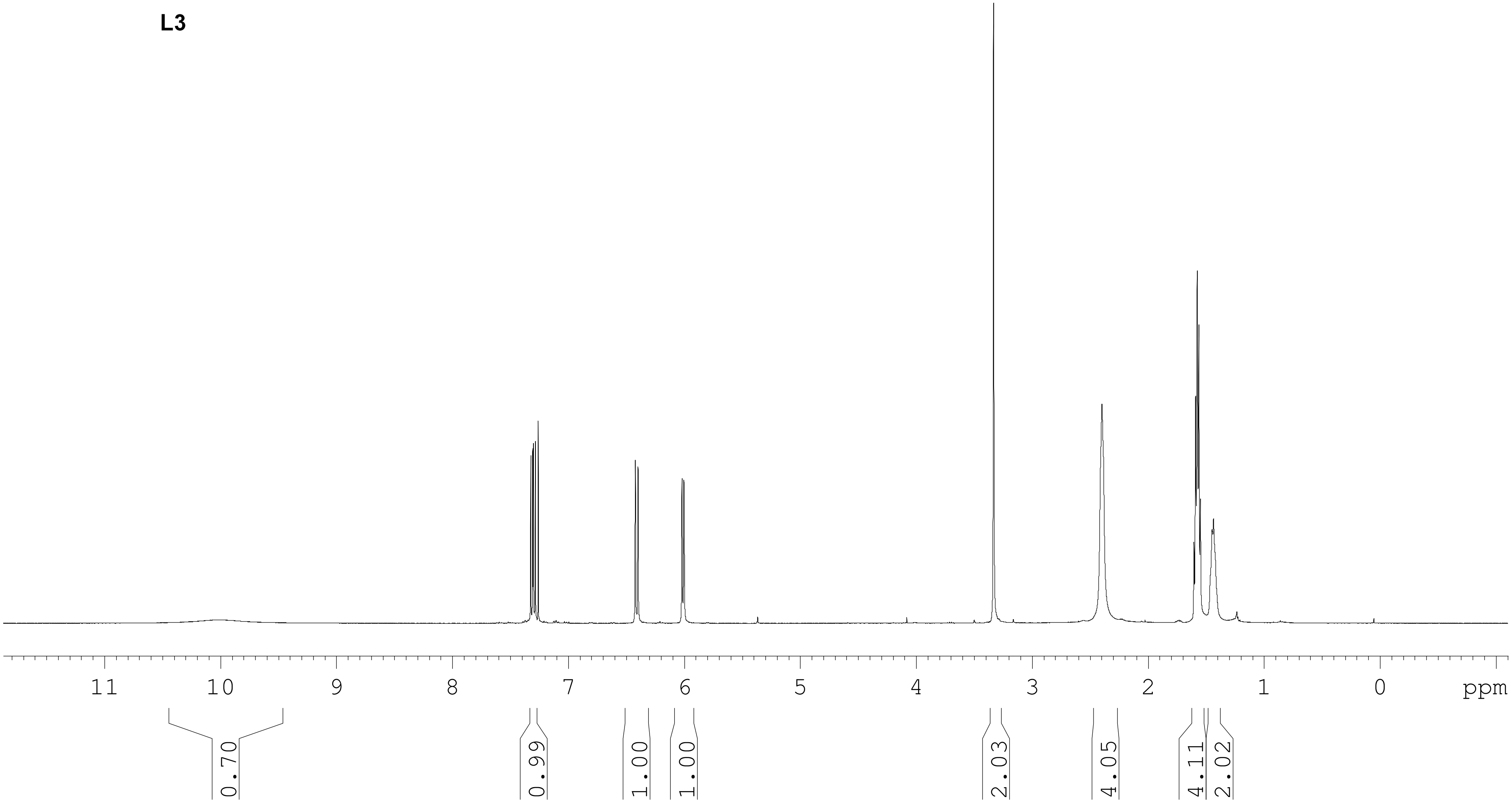

10.017
7.324
7.308
7.301
7.285
7.260
6.422
6.399
6.020
6.018
6.003
6.001
3.332
2.397
1.603
1.589
1.575
1.561
1.547
1.447
1.434
11
10
9
8
7
6
5
4
3
2
1
0
ppm
0.70
0.99
1.00
1.00
2.03
4.05
4.11
2.02

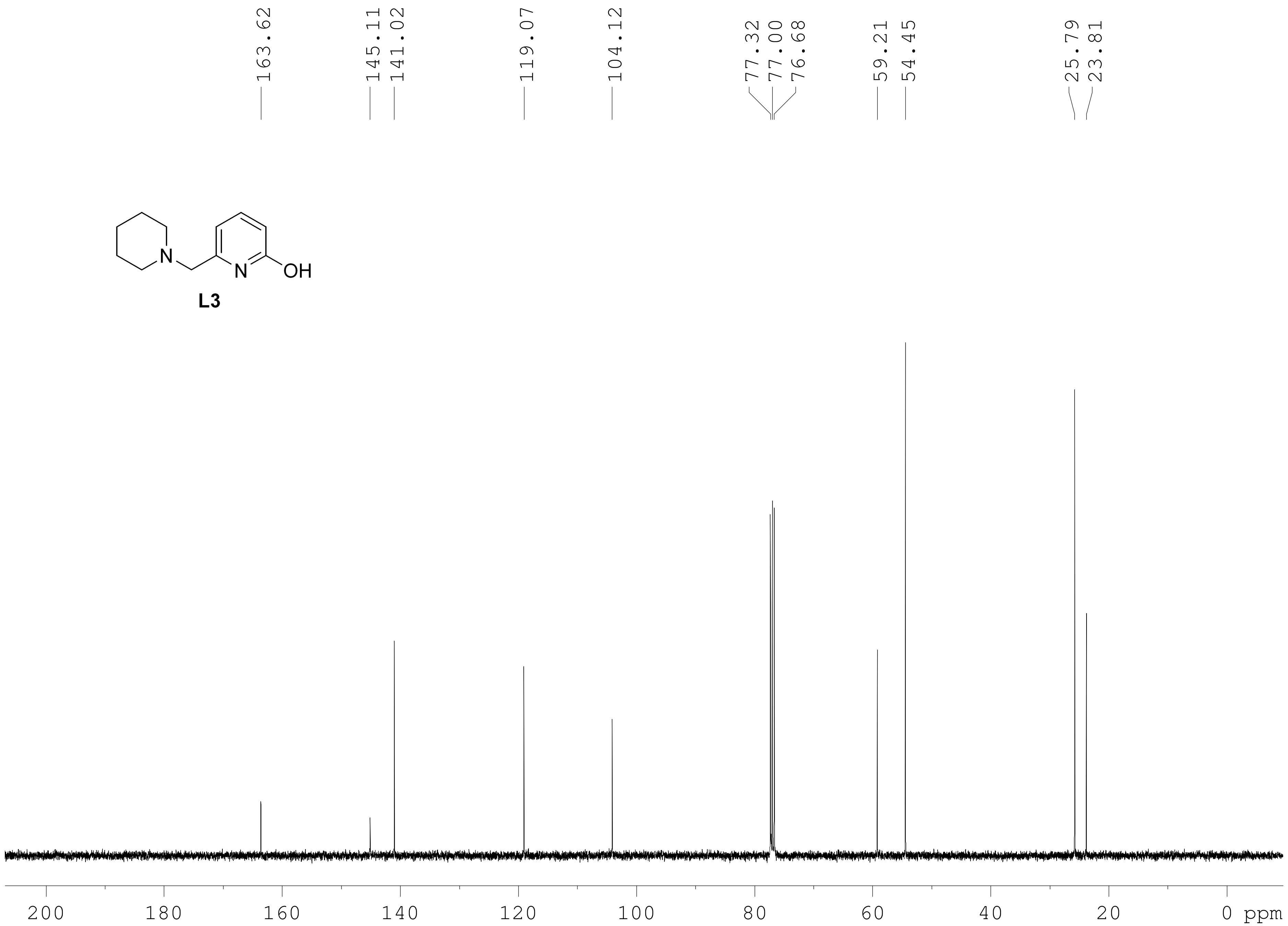
163.62
145.11
141.02
119.07
104.12
77.32
77.00
76.68
59.21
54.45
25.79
23.81
N
N
OH
L3
200
180
160
140
120
100
80
60
40
20
0 ppm

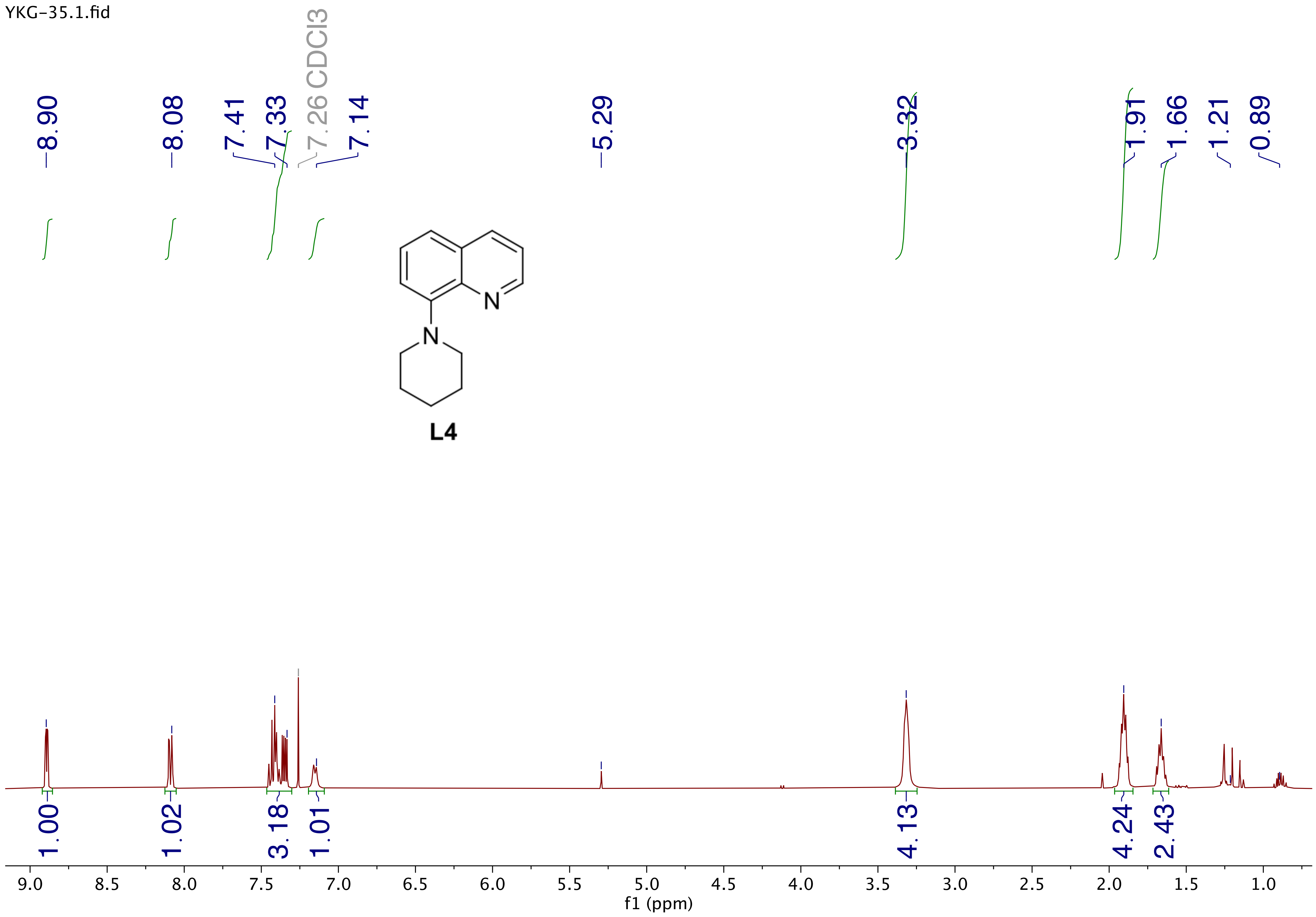

YKG-35.1.fid
8.90
8.08
7.41
7.33
7.26 CDCl3
7.14
5.29
3.32
1.91
1.66
1.21
0.89
N
N
L4
1.00
1.02
3.18
1.01
4.13
4.24
2.43
9.0
8.5
8.0
7.5
7.0
6.5
6.0
5.5
5.0
4.5
4.0
3.5
3.0
2.5
2.0
1.5
1.0
f1 (ppm)
